# HFrEF subphenotypes based on 4210 repeatedly measured circulating proteins are driven by different biological mechanisms

**DOI:** 10.1016/j.ebiom.2023.104655

**Published:** 2023-06-14

**Authors:** Teun B. Petersen, Marie de Bakker, Folkert W. Asselbergs, Magdalena Harakalova, K. Martijn Akkerhuis, Jasper J. Brugts, Jan van Ramshorst, R. Thomas Lumbers, Rachel M. Ostroff, Peter D. Katsikis, Peter J. van der Spek, Victor A. Umans, Eric Boersma, Dimitris Rizopoulos, Isabella Kardys

**Affiliations:** a Department of Cardiology, Erasmus MC, University Medical Center Rotterdam, Doctor Molewaterplein 40, Rotterdam, the Netherlands; b Department of Biostatistics, Erasmus MC, University Medical Center Rotterdam, Doctor Molewaterplein 40, Rotterdam, the Netherlands; c Amsterdam University Medical Centers, Department of Cardiology, University of Amsterdam, Meibergdreef 9, Amsterdam, the Netherlands; d Health Data Research UK and Institute of Health Informatics, University College London, Gower St, London, United Kingdom; e Department of Cardiology, Division Heart and Lungs, University Medical Center Utrecht, University of Utrecht, Heidelberglaan 100, Utrecht, the Netherlands; f Regenerative Medicine Center Utrecht, University Medical Center Utrecht, University of Utrecht, Heidelberglaan 100, Utrecht, the Netherlands; g Department of Cardiology, Northwest Clinics, Wilhelminalaan 12, Alkmaar, the Netherlands; h British Heart Foundation Research Accelerator, University College London, Gower St, London, UK; i Institute of Health Informatics, University College London, Gower St, London, UK; j Health Data Research UK London, University College London, Gower St, London, UK; k SomaLogic, Inc., 2945 Wilderness Pl, Boulder, United States; l Department of Immunology, Erasmus MC, University Medical Center Rotterdam, Doctor Molewaterplein 40, Rotterdam, the Netherlands; m Department of Pathology, Erasmus MC, University Medical Center Rotterdam, Doctor Molewaterplein 40, Rotterdam, the Netherlands

**Keywords:** Proteomics, Biomarkers, Phenotypes, Unsupervised machine learning, Heart Failure

## Abstract

**Background:**

HFrEF is a heterogenous condition with high mortality. We used serial assessments of 4210 circulating proteins to identify distinct novel protein-based HFrEF subphenotypes and to investigate underlying dynamic biological mechanisms. Herewith we aimed to gain pathophysiological insights and fuel opportunities for personalised treatment.

**Methods:**

In 382 patients, we performed trimonthly blood sampling during a median follow-up of 2.1 [IQR:1.1–2.6] years. We selected all baseline samples and two samples closest to the primary endpoint (PEP; composite of cardiovascular mortality, HF hospitalization, LVAD implantation, and heart transplantation) or censoring, and applied an aptamer-based multiplex proteomic approach. Using unsupervised machine learning methods, we derived clusters from 4210 repeatedly measured proteomic biomarkers. Sets of proteins that drove cluster allocation were analysed via an enrichment analysis. Differences in clinical characteristics and PEP occurrence were evaluated.

**Findings:**

We identified four subphenotypes with different protein profiles, prognosis and clinical characteristics, including age (median [IQR] for subphenotypes 1–4, respectively:70 [64, 76], 68 [60, 79], 57 [47, 65], 59 [56, 66]years), EF (30 [26, 36], 26 [20, 38], 26 [22, 32], 33 [28, 37]%), and chronic renal failure (45%, 65%, 36%, 37%). Subphenotype allocation was driven by subsets of proteins associated with various biological functions, such as oxidative stress, inflammation and extracellular matrix organisation. Clinical characteristics of the subphenotypes were aligned with these associations. Subphenotypes 2 and 3 had the worst prognosis compared to subphenotype 1 (adjHR (95%CI):3.43 (1.76–6.69), and 2.88 (1.37–6.03), respectively).

**Interpretation:**

Four circulating-protein based subphenotypes are present in HFrEF, which are driven by varying combinations of protein subsets, and have different clinical characteristics and prognosis.

**Clinical Trial Registration:**

ClinicalTrials.gov Identifier: NCT01851538https://clinicaltrials.gov/ct2/show/NCT01851538.

**Funding:**

EU/10.13039/100013322EFPIA IMI2JU BigData@Heart grant n°116074, Jaap Schouten Foundation and Noordwest Academie.

**Research in context**

**Evidence before this study**

HFrEF (Heart Failure with reduced Ejection Fraction), as a heterogeneous condition with a high mortality rate, can benefit greatly from subphenotyping efforts. Proteomic blood biomarkers carry large potential for phenotyping studies in the cardiovascular domain, as shown in studies in HFpEF (Heart Failure with preserved Ejection Fraction) patients.

**Added value of this study**

In this study, we identify four distinct HFrEF phenotypes based on 4210 repeatedly measured proteomic biomarkers. These subphenotypes show differing protein profiles, clinical characteristics and prognosis. The subphenotypes are driven by subsets of proteins that are associated with various pathophysiological pathways. Novel aspects of this study include the repeated assessments of the proteomic panel, in order to capture dynamic biological mechanisms; and the extensive set of proteins used. Our study demonstrates that repeated proteomics measurements improve the accuracy of subphenotypes in relation to prognosis compared to single baseline measurements. Moreover, the large protein panel provides a comprehensive assessment of the processes involved in heart failure, and gives us the opportunity to associate specific biological pathways and processes with the subphenotypes.

**Implications of all the available evidence**

Repeated assessment of extensive biomarker panels can contribute pathophysiological insights for complex diagnoses like HFrEF. Future studies should further investigate the role of these biological mechanisms in HFrEF and further explore prospects of personalized treatment decisions based on subphenotypes.

## Introduction

Heart Failure (HF) entails high mortality rates, and its prevalence is projected to increase in Western countries, among others, because of the ageing of the population.^1^^,^^2^ The causes and factors contributing to HF show substantial heterogeneity between patients.^3^ Yet, our understanding of this underlying pathophysiology, which could enhance current treatments, remains incomplete.^4^ This has spurred an interest in identifying subphenotypes of HF based on clinical, echocardiographic or hemodynamic characteristics.^5^, ^6^, ^7^, ^8^, ^9^ Such subphenotypes may provide further insights into underlying factors and mechanisms.

Recently, promising advancements have been made by identifying HF subphenotypes based on blood biomarkers, such as circulating proteins. Circulating proteins like NT-proBNP, ST2 and GDF-15 have shown to carry value for the prediction of cardiovascular events in HF patients.^10^ Using such biomarkers for subphenotyping has the advantage that these biomarkers may contain information about the patient's disease state that is not yet clinically apparent. Accordingly, patients may show different treatment response based on their biomarker profile.^11^ Recent studies on this topic in HF patients with preserved ejection fraction (HFpEF) have identified subphenotypes with varying clinical characteristics and prognosis, using 415^12^ and 363^13^ proteomic biomarkers. Studies like these in patients with heart failure with reduced ejection fraction (HFrEF) are scarce and have focused on clinical characteristics or a limited number of biomarkers.^3^^,^^11^

While the results of these previous studies are promising, several aspects warrant further investigation. First, incorporating larger numbers of circulating proteins could provide more comprehensive insights into the underlying mechanisms of HF, incremental to those provided by the biomarkers commonly investigated in cardiovascular studies. Second, by incorporating repeated proteomic measurements, temporal trends of the biomarker levels could be taken into account, which may allow for a better representation of the dynamic nature of HF.

Therefore, in this study, we considered repeated measurements of 4210 proteomic biomarkers in 382 HFrEF patients and conducted a cluster analysis on temporal proteomic patterns to identify HFrEF subphenotypes. Subsequently, we examined clinical characteristics of these subphenotypes, as well as their associations with adverse clinical events. Moreover, we used pathway analysis to further explore the proteomic profiles of the subphenotypes and the underlying disease mechanisms.

## Methods

### Study population and study design

The investigation was performed within the Serial Biomarker Measurements and New Echocardiographic Techniques in Chronic Heart Failure Patients Result in Tailored Prediction of Prognosis (Bio-SHiFT) study, conducted in the Erasmus MC, Rotterdam, and Northwest Clinics, Alkmaar, Netherlands. This was a prospective cohort study of stable patients suffering from chronic heart failure (CHF).^14^ Patients were recruited during regular outpatient visits and included if they were 18 years or older, able to understand and sign the informed consent form, if they were diagnosed with CHF ≥3 months ago according to the guidelines of the European Society of Cardiology,^15^^,^^16^ and if they had not been hospitalized for HF less than 3 months prior to inclusion. Information about the patients was recorded at baseline and at predefined follow-up visits, which were scheduled every 3 (±1) months. These visits included short medical examinations, collection of blood samples, and documentation of adverse cardiovascular events since the last visit. A description of the sample size estimation for the Bio-SHiFT study is provided in the supplemental material. A total of 398 CHF patients were enrolled between August 2011 and January 2018. This investigation concerns the 382 patients with HFrEF.

### Ethics statement

The study was approved by the medical ethics committee of the Erasmus Medical Center in Rotterdam (MEC-2011-029) and complied with the Declaration of Helsinki. All included patients provided written informed consent. The first and last authors had full access to the data of this study and take responsibility for its integrity and analysis.

### Clinical assessment at baseline

Every patient was evaluated at baseline by a research physician or research nurse, who performed a physical examination and recorded HF-related symptoms and NYHA class. Medical history, HF aetiology, left ventricular ejection fraction (EF), cardiovascular risk factors, and medication use were retrieved from hospital records.

### Outcome definitions

The study endpoints were determined by a clinical event committee consisting of three physicians, according to pre-defined event definitions; endpoints were determined based on hospital records and discharge letters, and without knowledge of the proteomic measurements. Any ambiguities were discussed by the physicians until consensus was reached. In patients with multiple endpoints, only the first one was considered for analysis. The primary endpoint (PEP) was pre-defined as the composite of cardiovascular death, heart transplantation, LVAD implantation, and hospitalization for acute or worsened HF. Patients were considered hospitalized for acute or worsened HF when hospitalized for an exacerbation of HF symptoms, together with two of the following conditions: BNP or NT-proBNP exceeding three times the normal upper limit, signs of worsening HF, like pulmonary rales, raised jugular venous pressure or peripheral edema, increased dose or intravenous administration of diuretics, or administration of positive inotropic agents.

### Proteomic measurements

Blood samples were collected at baseline and at trimonthly intervals thereafter. Blood samples used for this investigation included samples drawn at baseline, as well as the last two samples drawn before a PEP or the last two samples before censoring. Previous investigations in this cohort using all trimonthly samples have shown that the levels of several plasma and urine biomarkers change in the months before adverse events occur.^14^^,^^17^ By using the first and the last two samples, we capture these biomarker changes while minimizing the number of measurements. Blood samples were processed within 2 h after collection, after which they were stored at −80 °C.

We performed the proteomic analyses using 55 μL of EDTA plasma, using the aptamer-based proteomic SOMAscan platform.^18^ In total, 5284 aptamers were applied in the samples, of which 300 aptamers with non-human or not validated targets were subsequently excluded. Furthermore, when aptamers targeted the same protein, those with the highest binding affinity were kept, while the rest were excluded. This procedure left us with 4210 aptamers corresponding to an equal number of proteomic biomarkers. Additional information is provided in the Supplemental methods and Supplemental Table S1.

### Statistical analysis

Subphenotypes in the patient population were explored using a cluster analysis. We consecutively performed the cluster analyses on the baseline proteomics measurements and intercept and slope coefficients from linear mixed effect models (LME), representing the full trajectories of the proteins. Full details on the LME models and cluster analysis are provided in the supplement.

The biomarkers were log-transformed and standardized to Z-scores. Next, the dimensions of the biomarker profiles were reduced via the Uniform Manifold Approximation and Projection (UMAP) method. The optimal number of clusters was assessed by applying UMAP and the NbClust R-package, which uses an ensemble of measures to select the optimal number of clusters, on 100 bootstrap resampled datasets. The most common numbers of clusters were evaluated on cluster stability and clinical significance. Finally, the patients were assigned to different clusters via the k-means algorithm. Internal validity was assessed by investigating the stability of the cluster allocation under the addition of random noise.

Differences in proteins between the subphenotypes were illustrated via heatmaps and quantified using Kruskal–Wallis tests. Differences in the clinical characteristics were investigated using Kruskal–Wallis tests, chi-squared tests, or Fisher exact tests where appropriate. Differences in the hazard of PEP were investigated via Cox proportional hazard regressions, using four levels of correction: 1: univariate, 2: corrected for age, sex and eGFR (CKD-EPI), 3: corrected for all available clinical variables showing significant associations with the PEP according to Kruskal–Wallis tests after correction for multiple testing, with age, sex and eGFR (CKD-EPI) forced in the model, and 4: corrected for the variables from model 3 and NT-proBNP levels. Model 4 was included to make inferences on the added value of our subphenotypes over NT-proBNP, which is used in clinical practice, however caution is required when interpreting its results as clearly, subphenotypes were based on the proteomics measurements, which also included NT-proBNP. We therefore base our main inferences around the results of Model 3. C-indexes were internally validated using Harrell's bias correction.

The proteins were subsequently assigned into subsets containing similar proteins using k-means, with the optimal number of clusters chosen by NbClust. This was done separately for the slopes and intercepts. Protein subsets of interest were analysed using the ToppGene Suite^19^ with an enrichment analysis to identify associated diseases and biological processes, using the complete set of 4210 proteins as the reference. The results from this enrichment analysis were visualised using REVIGO.^20^

**Fig. 1:**
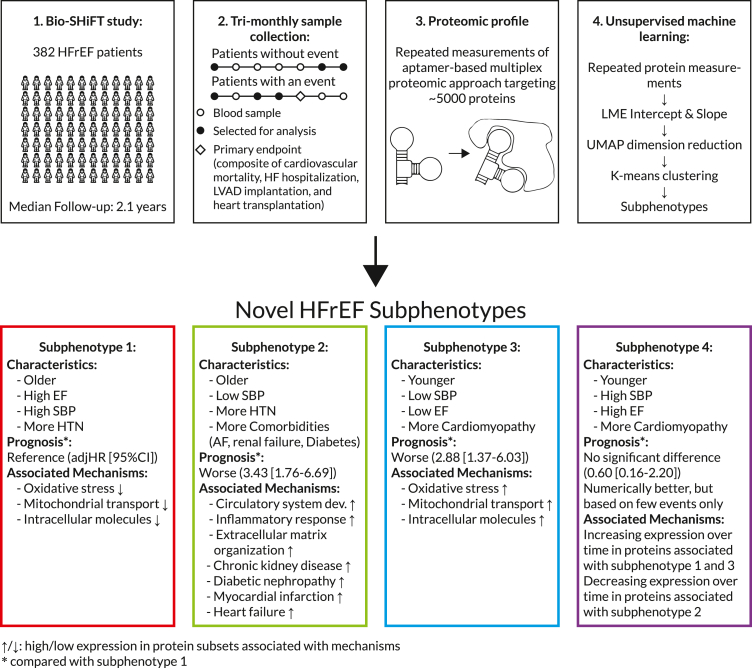
**Overview of methods and the subphenotypes that were obtained**. HFrEF = Heart Failure with reduced Ejection fraction, LME = Linear Mixed Effects, UMAP = Uniform Manifold Approximation and Projection, EF = Ejection Fraction, SBP = Systolic Blood Pressure, HTN= Hypertension, AF = Atrial fibrillation.

R version 4.0.3 was used for all analyses, and two-sided p-values <0.05 were considered statistically significant. An overview of the full methodology is displayed in Fig. 1.

### Role of the funding source

Funders were not involved in study design; in the collection, analysis and interpretation of data; in the writing of the report; or in the decision to submit the article for publication.

## Results

### Baseline characteristics

Baseline characteristics are shown in Table 1. The median (IQR) age was 64 (56–72), 72.8% were male and 72.3% of the patients were in NYHA class I or II. The median (IQR) duration of HF at inclusion was 4.2 (0.9–4.9) days. Measured at baseline, the median (IQR) NT-proBNP was 133 (46–274) pmol/L, median (IQR) hs-troponin T was 18 (9–33) ng/L and median (IQR) CRP was 2.2 (0.9–4.9) mg/L.

**Table 1:** Clinical characteristics.

Characteristic	Overall, N = 382^a^	1, N = 90^a^	2, N = 121^a^	3, N = 128^a^	4, N = 43^a^	p-value^b^
**Demographics**						
Age at baseline visit (years)	64 (56, 72)	70 (64, 76)	68 (60, 79)	57 (47, 65)	59 (56, 66)	**<0.001**
Men	278 (73%)	65 (72%)	88 (73%)	91 (71%)	34 (79%)	0.8
Ethnicity: Caucasian	351 (93%)	88 (99%)	110 (92%)	112 (88%)	41 (98%)	**0.005**
**Features of HF**						
Duration (years)	4.2 (1.6, 9.5)	2.7 (0.8, 6.5)	5.6 (2.8, 10.8)	4.2 (1.4, 10.3)	3.4 (1.8, 6.5)	**0.005**
NYHA class I or II	276 (73%)	68 (76%)	71 (59%)	101 (80%)	36 (84%)	**<0.001**
Systolic ejection fraction (%)	30 (23, 36)	30 (26, 36)	26 (20, 38)	26 (22, 32)	33 (28, 37)	**0.009**
**Clinical characteristics**						
BMI (kg/m2)	26.5 (24.0, 30.1)	26.0 (24.3, 28.6)	25.9 (23.4, 29.3)	26.9 (24.3, 30.6)	28.9 (24.8, 31.3)	0.1
eGFR CKD-EPI (mL/min/1.73m2)	58 (42, 77)	56 (44, 73)	53 (38, 73)	68 (55, 85)	74 (54, 87)	**0.002**
Systolic blood pressure (mmHg)	114 (100, 130)	124 (108, 138)	112 (100, 128)	110 (94, 120)	120 (110, 129)	**<0.001**
Diastolic blood pressure (mmHg)	70 (60, 78)	74 (68, 81)	68 (60, 76)	70 (60, 75)	70 (65, 80)	**<0.001**
**Biomarker level**						
Nt-proBNP (pmol/L)	133 (46, 274)	94 (46, 204)	252 (156, 458)	71 (20, 169)	81 (27, 163)	**<0.001**
Hs-Troponin T (ng/L)	18 (9, 33)	16 (10, 26)	28 (17, 44)	10 (7, 23)	10 (8, 15)	**<0.001**
CRP (mg/L)	2.2 (0.9, 4.9)	1.8 (0.6, 3.4)	3.1 (1.3, 6.7)	3.0 (1.3, 5.5)	1.3 (0.7, 2.2)	**<0.001**
**Aetiology of HF**						
Ischaemic heart disease	166 (43%)	44 (49%)	52 (43%)	47 (37%)	23 (53%)	0.2
Hypertension	33 (8.6%)	13 (14%)	18 (15%)	2 (1.6%)	0 (0%)	**<0.001**
Secondary to valvular heart disease	12 (3.1%)	3 (3.3%)	6 (5.0%)	3 (2.3%)	0 (0%)	0.5
Cardiomyopathy	122 (32%)	15 (17%)	37 (31%)	57 (45%)	13 (30%)	**<0.001**
Hypertrophic (HCM)	15 (3.9%)	1 (1.1%)	9 (7.4%)	5 (3.9%)	0 (0%)	0.077
Dilated (DCM)	97 (25%)	13 (14%)	24 (20%)	49 (38%)	11 (26%)	**<0.001**
Restrictive	0 (0%)	0 (0%)	0 (0%)	0 (0%)	0 (0%)	>0.9
Arrhytmogenic right ventricular (ARVC)	1 (0.3%)	0 (0%)	1 (0.8%)	0 (0%)	0 (0%)	0.7
Non compaction cardiomyopathy	4 (1.0%)	0 (0%)	1 (0.8%)	2 (1.6%)	1 (2.3%)	0.6
Unclassified	7 (1.8%)	1 (1.1%)	2 (1.7%)	3 (2.3%)	1 (2.3%)	>0.9
Unknown	27 (7.1%)	12 (13%)	5 (4.1%)	8 (6.2%)	2 (4.7%)	0.081
**Medical history**						
Myocardial Infarction	145 (38%)	34 (39%)	42 (35%)	49 (38%)	20 (47%)	0.6
PCI	126 (33%)	26 (29%)	40 (33%)	39 (30%)	21 (49%)	0.11
CABG	54 (14%)	15 (17%)	21 (17%)	10 (7.8%)	8 (19%)	0.092
Atrial fibrillation	137 (36%)	34 (39%)	62 (52%)	26 (20%)	15 (35%)	**<0.001**
Other arrhythmia	151 (40%)	17 (19%)	49 (41%)	64 (50%)	21 (49%)	**<0.001**
pacemaker implantation	85 (23%)	8 (9.5%)	27 (24%)	35 (27%)	15 (35%)	**0.004**
ICD implantation	254 (66%)	52 (58%)	76 (63%)	97 (76%)	29 (67%)	**0.032**
CRT	113 (30%)	26 (29%)	35 (29%)	40 (31%)	12 (28%)	>0.9
Stroke (CVA/TIA)	48 (13%)	17 (19%)	16 (14%)	12 (9.4%)	3 (7.0%)	0.12
Chronic renal failure	181 (48%)	40 (45%)	79 (65%)	46 (36%)	16 (37%)	**<0.001**
Diabetes Mellitus	98 (26%)	15 (17%)	50 (41%)	24 (19%)	9 (21%)	**<0.001**
Known hypercholesterolemia	160 (43%)	28 (32%)	52 (44%)	55 (44%)	25 (60%)	**0.026**
Hypertension	166 (44%)	32 (37%)	58 (48%)	53 (42%)	23 (53%)	0.2
**Intoxication**						
Smoking: Ever	271 (71%)	68 (76%)	81 (67%)	86 (68%)	36 (84%)	0.13
Smoking: Current	343 (90%)	80 (89%)	111 (92%)	113 (90%)	39 (91%)	0.9
**Medication**						
Ace Inhibitor	258 (68%)	58 (65%)	74 (61%)	91 (71%)	35 (81%)	0.072
Angiotensin II receptor blockers	107 (28%)	29 (32%)	38 (31%)	33 (26%)	7 (16%)	0.2
Aldosteron antagonists	293 (77%)	72 (80%)	90 (74%)	106 (83%)	25 (58%)	**0.008**
Diuretics other	5 (1.3%)	0 (0%)	1 (0.8%)	2 (1.6%)	2 (4.7%)	0.2
Beta blockers	350 (92%)	79 (89%)	107 (88%)	122 (95%)	42 (98%)	0.069
Aspirin	77 (20%)	13 (15%)	24 (20%)	23 (18%)	17 (40%)	**0.007**

p-values <0.05 are indicated in bold. BMI = Body mass index, CABG = Coronary artery bypass surgery, CRT = Cardiac resynchronisation therapy, ICD = implantable cardioverter-defibrillator, PCI = Percutaneous coronary intervention.

a Median (IQR); n (%).

b Kruskal–Wallis rank sum test; Pearson's Chi-squared test; Fisher's exact test.

### Cluster analysis

**Fig. 2:**
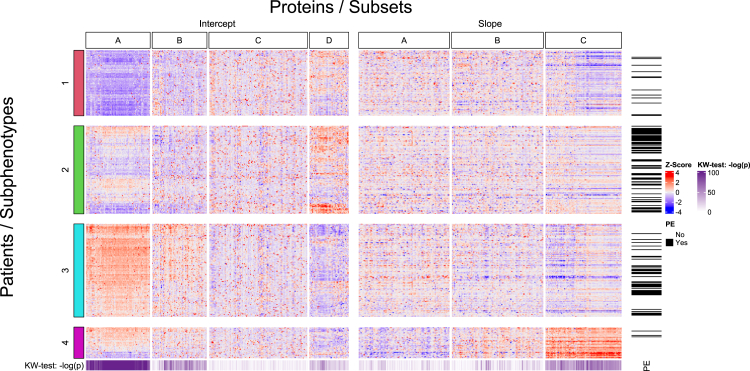
**Heatmap displaying intercept/slope levels across subphenotypes and protein subsets**. Patients and subphenotypes are on the Y-axis, while proteins and protein subsets are on the X-axis. The purple heatmap at the bottom displays the –log (p-value) of Kruskal–Wallis tests per protein, and the black lines on the side indicate the occurrence of an endpoint.

In the main text, we focus on the cluster analysis performed using the estimated intercept and slope coefficients. Results of cluster analyses on baseline, second and final proteomics measurements are provided in the supplementary material. Aggregated results from our bootstrap analysis to find the optimal number of clusters can be found in Supplemental Fig. S1. Either two, three or four clusters seemed to fit the data best. Fitting three clusters resulted in suboptimal cluster stability. Fitting four clusters led to subphenotypes that showed more diversity in clinical characteristics and biomarker profiles than fitting two clusters. Hence, four clusters was chosen as the best solution for our study. The Jaccard similarity values from the stability analysis showed that all four clusters were stable (cluster 1: 0.83, 2: 0.77, 3: 0.92, 4: 0.89). Fig. 2 illustrates the distribution of protein values across the clusters using a heatmap.

### Cluster characteristics

Baseline characteristics per cluster are presented in Table 1. Patients with subphenotypes 1 and 2 were the two oldest groups and more often showed hypertensive aetiology of HF, while patients with subphenotypes 3 and 4 were youngest and more frequently showed (dilated) cardiomyopathy. Subphenotype 2 had lower systolic blood pressure (mean subphenotype 1 - mean subphenotype 2 (95%CI): SBP1-SBP2: 9.91 (4.09–15.73) mmHg) and subphenotype 3 lower systolic blood pressure and EF than subphenotype 1 (SBP1-SBP3: 15.73 (10.09–21.36) mmHg; EF1-EF3: 4.60 (1.54–7.66)%). Subphenotype 2 showed the highest median levels of NT-proBNP, hs-troponin T and CRP at baseline, the lowest percentage of patients with NYHA class I or II, the longest median duration of HF and these patients most often had a history of chronic renal failure or diabetes mellitus. Subphenotype 4 had the lowest median levels of CRP and the highest percentage of patients with NYHA class I or II.

### Associations between clusters and clinical outcome

**Fig. 3:**
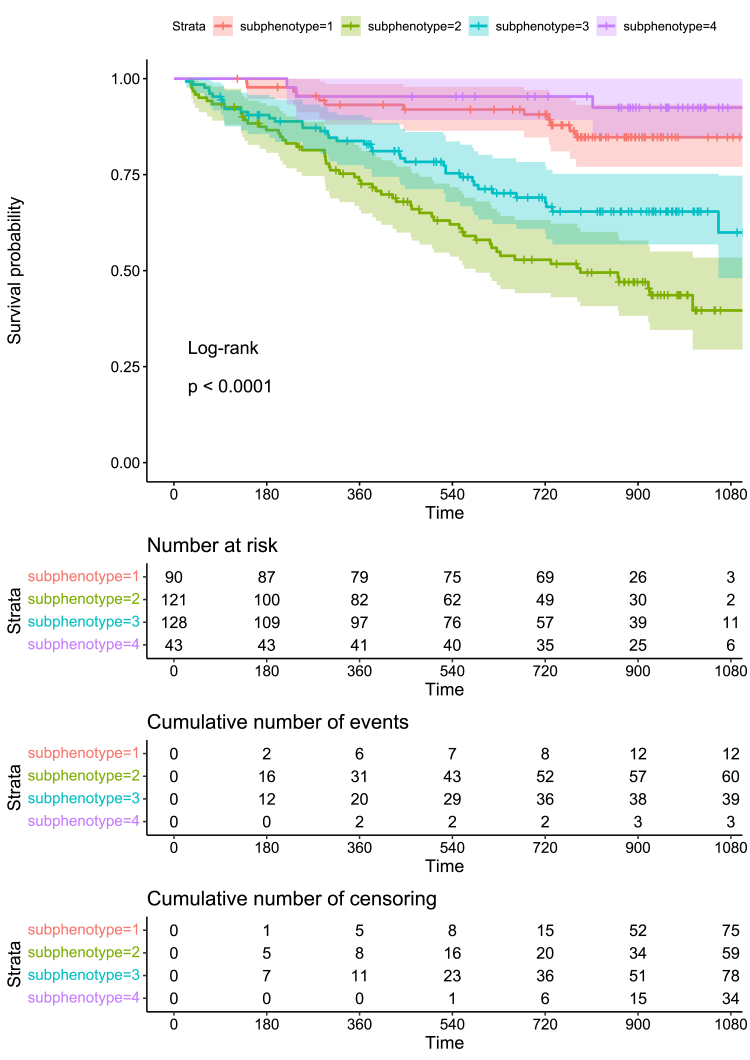
**Kaplan–Meier plot illustrating the differences in prognosis for the subphenotypes**. Endpoints are the composite of cardiovascular mortality, HF hospitalization, LVAD implantation and heart transplantation. The survival curves differ significantly from each other with a log-rank p-value <0.0001.

**Fig. 4:**
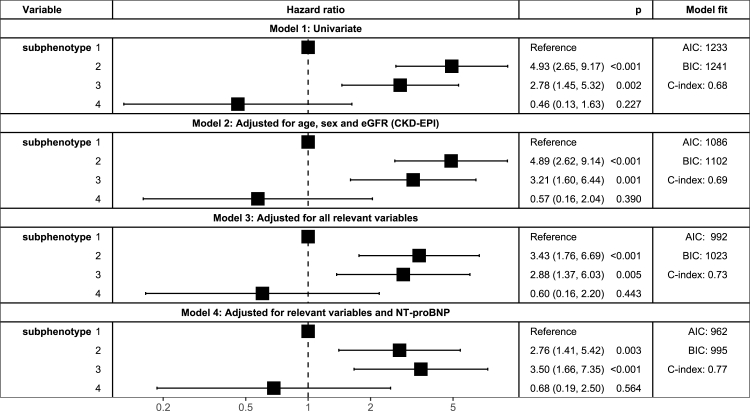
**Forest plot illustrating the differences in prognosis for the subphenotypes**. Forest plot illustrating prognosis of the subphenotypes using Cox proportional hazard models with four levels of adjustment: 1. Univariate, 2. Adjusted for age, sex and eGFR (CKD-EPI), 3. Adjusted for age, sex, eGFR (CKD-EPI), systolic blood pressure, duration of HF, NYHA class, history of atrial fibrillation, other arrhythmia and chronic renal failure 4. Adjusted for the variables of model 3 and baseline NT-proBNP. C-indexes are optimism corrected via Harrell's bias correction. AIC = Akaike information criterion, BIC= Bayesian information criterion, p = p-value.

Median follow-up was 2.06 (IQR: [1.10.2.57]) years. In total, 114 patients experienced a PEP. Supplemental Table S2 displays the clinical characteristics of patients with and without a PEP. Age, sex, SBP, duration of HF, NYHA class, history of atrial fibrillation, other arrhythmia, and chronic renal failure differed significantly between patients with and without a PEP. Fig. 3 shows a Kaplan–Meier plot illustrating the differences in prognosis for the subphenotypes, and Fig. 4 displays the results of the univariate and adjusted Cox proportional hazard models. Adjusted for aforementioned variables, compared to subphenotype 1, subphenotypes 2 and 3 had the worst outcomes (HR (95%CI): cluster 2: 3.43 (1.76–6.69), cluster 3: 2.88 (1.37–6.03)). Subphenotype 4 did not show a significant association with outcome compared to subphenotype 1. Of note is that this subphenotype 4 consisted of only 43 patients, with only three events. The optimism corrected C-statistic for this model was 0.73.

### Proteins associated with cluster membership

The distribution of protein values suggested that cluster allocation was driven by highly correlated groups of biomarkers. Using a combination of k-means and NbClust to identify relevant subsets of biomarkers, separately for the intercept and slope parameters, resulted in the partitioning for the intercept and slope values of the proteins as displayed in Fig. 2.

For the intercepts, four subsets were deemed optimal; they are denoted with the letters A, B, C and D. Subset A seemed most important for cluster allocation with 99.7% (1051/1054) of proteins significantly associated with the estimated subphenotypes after correction for multiple testing. This was followed by subsets B and D, with respectively 68.6% (615/897) and 57.8% (372/643), and finally subset C with 19.6% (317/1615). The top 10 proteins most associated with the subphenotypes per subset are displayed in Supplemental Table S3. Protein levels in subsets A and B were highest in subphenotypes 3 and 4 and lowest in subphenotype 1. Protein levels in subset D were highest in subphenotype 2.

Subset D included many proteins that are implicated in various pathways in heart failure: myocardial stretch/stress and fibrosis (NT-proBNP, ANP, ST2, GDF15, and FGF23), extracellular matrix remodelling (MMP2 and TIMP1), myocyte injury (troponin T, heart-type fatty acid-binding protein and sFAS), inflammation (prolactinon, adiponectin, soluble endoglin, FAS (APO-1), osteoprotegerin and CA-125), neurohumoral activation (adrenomedullin, chromogranin A and B) and renal dysfunction (cystatin C and kidney injury molecule-1). Subsets A and B included proteins associated with extracellular matrix remodelling (A: IL-6, B: MMP9), myocyte injury (B: CK-MB), and inflammation (A: IL-6). Subset C included proteins associated with extracellular matrix remodelling (MMP8, and galectin-3), oxidative stress (MPO), inflammation (CRP, TNF-alpha, LP-PLA2, TWEAK, Serine protease PR3, S100A8/A9 complex), and neurohumoral activation (angiotensin II and endothelin-1, 2 and 3).

**Fig. 5:**
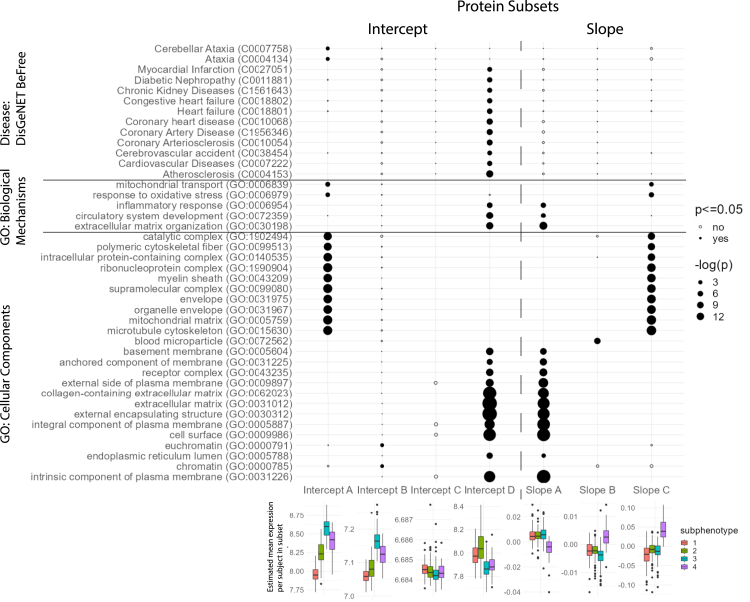
**Associations between protein subsets and, biological conditions and mechanisms**. Summary of Benjamini-Hochberg corrected p-values obtained from an enrichment analysis using the Toppgene Suite on the Gene Ontology Cellular Component and Biological Mechanisms datasets and the DisGeNet BeFree disease dataset.^21^, ^22^ Terms are on the y-axis and the various subsets are on the x-axis. The size of the dots is proportional to –log(p), B–H p-values equal to 1 are omitted. Boxplots illustrate the mean intercept/slope values of all proteins in each of the protein subsets per patient (estimated via a random intercepts model), per subphenotype. For GO: Cellular Component, the top 10 significant terms with the lowest B–H p-value were selected to be displayed. For the diseases, the top 10 significant terms with the lowest B–H p-value per subset were selected to be displayed, disregarding conditions related to cancer, dermatology or the digestive system. For the GO: Biological Mechanisms, terms were selected based on significance and relevance for the PEP.

Fig. 5 displays the main results from our enrichment analysis. Associations of the protein subsets with ‘biological processes’ and ‘cellular components’ from the Gene Ontology database^22^ and ‘diseases’ from the DisGeNET BeFree database^21^ are highlighted based on the magnitude of their Benjamini–Hochberg corrected p-values. Notable associations with biological processes and diseases for subset A included response to oxidative stress (B–H pval:7.87E-03), mitochondrial transport (3.73E-03) and ataxia (3.10E-02). Subset D was associated with circulatory system development (3.79E-06), inflammatory response (3.90E-04), extracellular matrix organization (2.10E-06), chronic kidney disease (2.49E-03), diabetic nephropathy (1.23E-03), atherosclerosis (3.25E-6), and conditions related to the PEP such as heart failure (5.79E-04) and myocardial infarction (3.59E-03). In the dot plot for the cellular components, associations for subset A included intracellular terms (Term:B–H pval; mitochondrial matrix:5.62E-10, microtubule cytoskeleton:6.43E-10), while cellular component associations for subset D were of a more extracellular nature (extracellular matrix:2.89E-25, external encapsulating structure:2.89E-25).

Supplemental Tables S4–S6 provide full overviews of the significant results of the enrichment analysis, respectively for ‘diseases’, ‘biological processes’, and ‘cellular components’. We found the following top 3 associated ‘biological processes’ per intercept subset, for subset A: intracellular transport (B–H pval: 8.79E-15), protein translation (1.43E-14) and peptide biosynthetic process (1.43E-14); for subset B, regulation of chromosome segregation (1.94E-02) and regulation of sister chromatid segregation (4.32E-02); for subset C, adaptive immune response (1.59E-02), humoral immune response mediated by circulating immunoglobulin (1.59E-02), immune effector process (1.59E-02); and for subset D, cell adhesion (2.38E-18), locomotion (2.12E-13) and taxis (6.25E-13). Biological processes that were previously found to be highly associated with HFrEF in other studies making use of the Gene Ontology database,^23^^,^^24^ were also reflected in our subsets. Peptidyl-serine phosphorylation (1.19E-02) was associated with subset A, and plasma membrane bounded cell projection morphogenesis (1.70E-07), ERK1 and ERK2 cascade (1.92E-04), response to wounding (1.75E-03), mesenchyme development (2.94E-02), and MAPK cascade (3.18E-02) were associated with subset D.

**Fig. 6:**
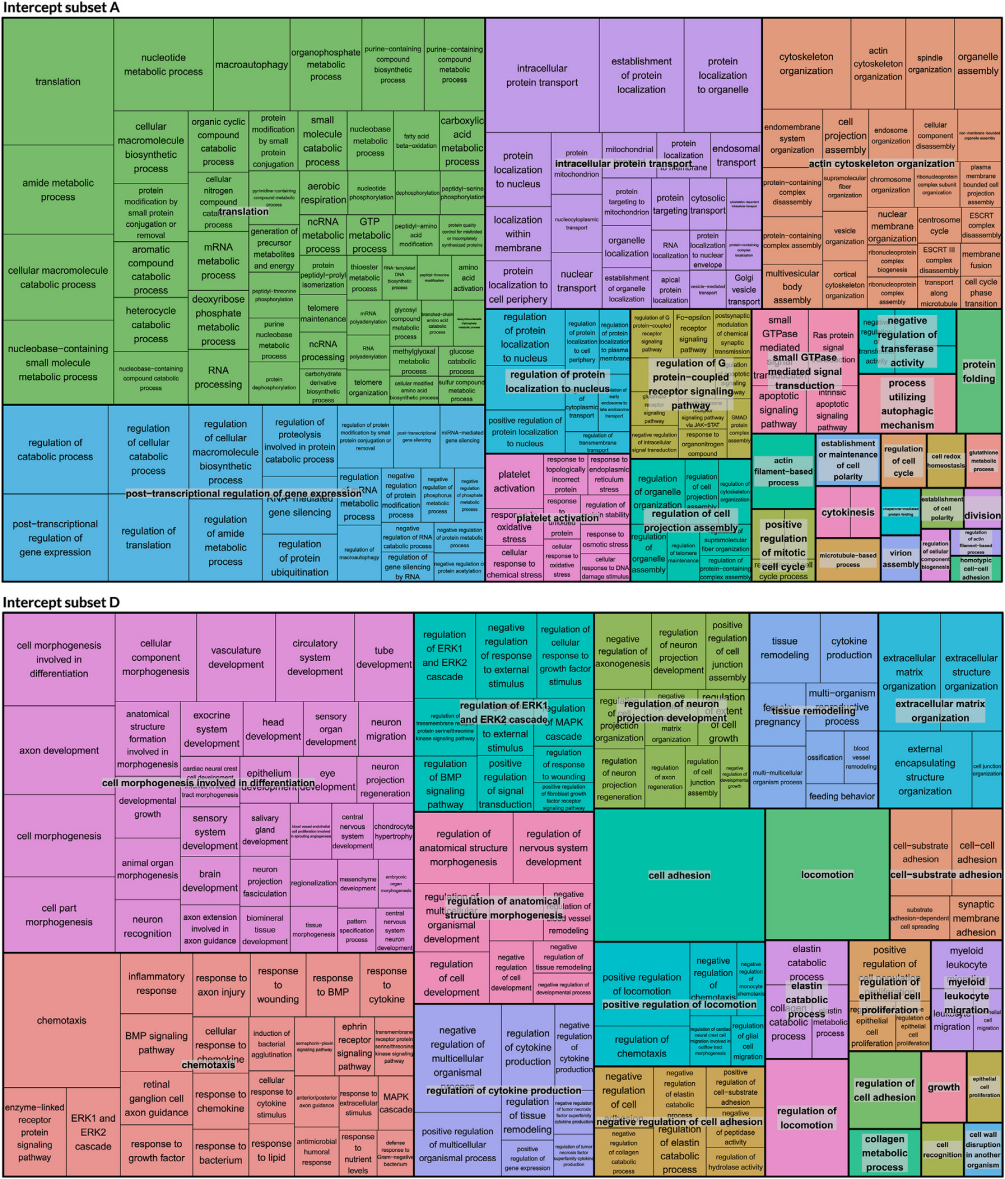
**Summary of Gene Ontology biological processes associated with intercept subset A and D**. Treemaps of Gene Ontology (GO) biological processes associated with Intercept subset A and D, generated using REVIGO, a visualization tool that groups closely related GO terms together based on network analysis.^20^ Groups of closely related GO terms are plotted together in the same colour. Representative GO terms, selected based on p-value, are superimposed over all groups. Size of the GO term blocks is proportional to the –log (B–H p-value) of their association with the subset. Not all significant biological processes are represented as REVIGO removes some redundant terms that are very similar to other terms.

Visual summaries of biological processes associated with intercept subsets A and D are provided in Fig. 6. For subset A, the summary highlights translational processes, as well as processes involved in intracellular protein transport, actin cytoskeleton organization and post-transcriptional regulation of gene expression. Conversely, for subset D, the summary highlights processes involved in cell morphogenesis and differentiation, and those involved in chemotaxis.

A similar analysis of protein subsets was conducted for the slope coefficients. Here, three subsets were deemed optimal, as illustrated in Fig. 2. The findings were in strong agreement with the intercept analysis: 98% of proteins in intercept subset A were present in slope subset C, and 91% of the proteins in intercept subset D were present in slope subset A. Furthermore, in Fig. 5 we see aligning associations between intercept subset A and slope subset C, and intercept subset D and Slope subset C.

### Clusters based on single protein measurements

In the analysis using baseline protein measurements only, three stable clusters were found (Jaccard Similarity cluster 1: 0.94, 2: 0.98, 3: 0.96) after bootstrapping NbClust (Supplemental Fig. S2). Supplemental Fig. S3 displays the allocation of the patients over the clusters via a heatmap. Subphenotype 1 was oldest, had the highest median systolic blood pressure and EF, and the lowest median CRP. Subphenotype 2 had the highest NT-proBNP. Subphenotype 3 was the youngest, had the lowest median NT-proBNP and hs-troponin T, and these patients were least often diagnosed with ischaemic heart disease and hypertension, and most often with cardiomyopathy (Supplemental Table S7). Subphenotypes 2 and 3 showed numerically higher rates of the PEP compared to subphenotype 1, although the associations were less outspoken compared to the repeated-measurements based subphenotypes, and did not reach statistical significance (Supplemental Fig. S4; Supplemental Table S8).

In the analysis using second measurements, three stable clusters were found (Jaccard Similarity cluster 1: 0.97, 2: 0.97, 3: 0.93) after bootstrapping NbClust (Supplemental Fig. S5). The allocation of patients over the clusters is illustrated in Supplemental Fig. S6. Clinical characteristics are provided in Supplemental Table S9. Subphenotype 1 was oldest, had the lowest prevalence of cardiomyopathy, and lowest history of pacemaker implantation. Subphenotype 2 had the highest prevalence of diabetes mellitus and chronic renal failure. While subphenotype 3 was youngest, patients had highest eGFR (CKD-EPI), lowest systolic blood pressure, lowest prevalence of hypertension, highest prevalence of cardiomyopathy and pacemaker implantation, and lowest history of chronic renal failure. The prognosis of subphenotype 2 was significantly worse than that of subphenotype 1, after adjusting for relevant clinical variables (Supplemental Fig. S7, Supplemental Table S10). The 25 patients with only one measurement (for example, because the PEP occurred early during follow-up) were included in this analysis.

Similar patterns can be seen in the analysis using the last available biomarker measurements before PEP or censoring. Three stable clusters were found (Jaccard Similarity cluster 1: 0.92, 2: 0.82, 3: 0.94) after bootstrapping NbClust (Supplemental Fig. S8). Supplemental Fig. S9 displays the allocation of the patients over the clusters via a heatmap. Subphenotype 1 had the highest median systolic blood pressure and EF, and lowest prevalence of cardiomyopathy. Subphenotype 2 had the lowest median EF and most often a history of atrial fibrillation, chronic renal failure and diabetes mellitus. Patients with subphenotype 3 were least often diagnosed with hypertension, most often with cardiomyopathy and younger compared to the other subphenotypes (Supplemental Table S11). The prognosis of subphenotype 2 was significantly worse compared to subphenotype 1, when adjusting for relevant clinical variables (Supplemental Fig. S10, Supplemental Table S12).

Overall, both the baseline protein-based subphenotypes, second measurement protein-based subphenotypes and the last available measurement protein-based subphenotypes showed similarities to the first three subphenotypes of the analysis using intercept and slope values, both concerning clinical characteristics and proteins patterns.

## Discussion

In this observational study of 382 HFrEF patients, the application of unsupervised clustering techniques on 4210 repeatedly measured circulating cardiovascular proteins, identified four distinct HFrEF subphenotypes that showed differences in baseline characteristics and clinical outcomes. Baseline characteristics that were significantly associated with the subphenotypes included age, systolic blood pressure, NYHA class, EF, diabetes mellitus, and chronic renal failure. Certain protein subsets seemed to drive the cluster allocation, and an enrichment analysis showed that these subsets were related to different biological mechanisms which are associated with heart failure^25^ such as inflammation, oxidative stress, renal dysfunction and extracellular matrix remodelling.

Phenotyping studies are still scarce in the domain of HFrEF. Previously, Ahmad et al.,^3^ examined 1619 patients with chronic HFrEF and constructed phenotypes based on 45 clinical characteristics. Tromp et al.,^11^ included 1802 patients with HFrEF and used 92 cardiovascular biomarkers measured once at baseline for phenotyping. In the current investigation, we expand this previous evidence in two ways. First, we utilize a large panel of biomarkers. HF is a complex condition that is linked with many biological processes.^2^ Previous studies focused on relatively narrow sets of cardiovascular proteins or clinical characteristics. Incorporating a diverse set of biomarkers enables us to gain a broader perspective on the mechanisms involved in HF. The SOMAscan assays have shown promising results in the field of biomarker discovery, and were found to be consistent with protein quantitative trait loci (pQTLs). Second, we use repeated protein measurements, which allows us to account for protein changes over time via the estimated intercept and slope coefficients. This is important as previous studies have observed that changes in biomarker levels provide valuable information, especially in the period before adverse clinical events occur.^14^^,^^17^ Recently, a preliminary study has been performed using data from the Bio-SHiFT study, wherein 92 circulating proteins were repeatedly measured in a subset of 263 patients from the first inclusion round of the same cohort and derived HFrEF subphenotypes. Here three subphenotypes were identified, with varying clinical characteristics and prognosis. In contrast with the current study, no clear protein subsets characterizing individual phenotypes were found; cluster 1 showed lower values of all biomarkers, cluster 2 showed increasing levels over time of most biomarkers, while in cluster 3, there were elevated baseline levels, and increasing levels over time of the remaining biomarkers. These findings may have resulted from the fact that the investigated 92-biomarker panel contained proteins which had previously been linked to cardiovascular disease. A broader panel, as used here, might be better able to identify cluster-specific protein subsets.

We identified four subphenotypes. In brief, subphenotype 1 consisted of relatively old patients with high SBP and high prevalence of hypertension, but with higher EF and better clinical outcomes. The biomarker profile of subphenotype 1 was characterized by low expression at baseline of proteins in subsets A and B. Subset A was related to oxidative stress, ataxia and intracellular components. Overall, prominent underlying biological processes included those related to translational processes, as well as those involved in intracellular protein transport, actin cytoskeleton organization and post-transcriptional regulation of gene expression. Specifically, top associated biological processes for subset A were related to intracellular transport and protein translation, which can also be linked to cardiovascular diseases via oxidative stress.^26^ Other notable biological processes included platelet activation, which is associated with complications in acute HF,^27^ and the G-protein-coupled receptor signalling pathway. G-protein-coupled receptors play a central role in cardiac function and disease, and are major drug targets for a variety of cardiovascular diseases such as HF, coronary artery disease and hypertension.^28^ Subset B did not display any strong associations with diseases, biological processes or cellular components based on the expression analysis. Subphenotype 2 also consisted of older patients, but with lower SBP, more comorbidities such as diabetes mellitus and chronic renal failure, very high NT-proBNP and hs-troponin, and worse clinical outcomes. The biomarker profile of subphenotype 2 was in line with these characteristics, as it showed high expressions of proteins in subset D, which was associated with inflammation, extracellular matrix remodelling, chronic renal failure, diabetic nephropathy, heart failure and myocardial infarction. Overall, prominent underlying biological processes in subset D included those related to cell morphogenesis and differentiation, and those related to chemotaxis. Specifically, top associated biological processes for subset D were related to cell adhesion and cell migration (chemotaxis, locomotion), both of which can be linked to adverse prognosis in heart failure via inflammation.^29^^,^^30^ Other biological processes included ERK1/2 (extracellular signal-regulated kinase) signalling pathway and BMP (bone morphogenetic protein) signalling pathway. ERK1/2 mediates cardiac hypertrophy and is associated with protection from cell death and ischaemic injury.^31^ BMP plays a fundamental role in the homeostasis of the heart, and perturbations of its signalling pathway are associated with various cardiovascular diseases such as atherosclerosis, vascular inflammation, and pulmonary hypertension.^32^ Conversely, subphenotype 3 consisted of younger patients with lower SBP, low EF, and higher prevalence of, in particular dilated, cardiomyopathy. These patients also had worse clinical outcomes. Subphenotype 3 had elevated levels of proteins in subset A and B, with over-representation of processes as described above. Subphenotype 4 was small and thus more difficult to assess. It differed from subphenotype 3 by having a higher SBP and higher EF; clinical outcomes seemed better. Biomarkers of subphenotype 4 were characterized by elevated levels for the slope coefficients in slope subset C and low levels for slope coefficients in subset A, which respectively contain many proteins found in intercept subsets D and A.

Subphenotype 2 found in the current study shows similarities to cluster 1 described by Ahmad et al.^3^ and Endotype 4 described by Tromp et al.,^11^ which also showed worse clinical outcomes, high rates of atrial fibrillation, and high levels of NT-proBNP. Our subphenotype 2 was characterized by elevated protein levels in subset D, which includes many known HF biomarkers, including NT-proBNP, ST2, troponin T, and GDF15. Tromp et al.'s^11^ Endotype 4 also showed similarities on SBP and EF. Subphenotypes similar to our subphenotypes 1, 3, and 4 were not found in either previous study. The fact that overlap between the clusters from different studies is limited may in part be because Ahmad et al.^3^ and Tromp et al.^11^ used different variables to define their clusters (respectively clinical variables and a smaller set of proteomic biomarkers, measured at baseline only), and because of differences in the CHF cohorts used. Using clinical variables as the basis of the cluster analysis process, as Ahmad et al. did, will by definition lead to large differences in these variables between the clusters. This hampers comparison with our subphenotypes, which are solely based on proteomic measurements. When subphenotyping is driven fully by proteomic profiles, any differences in clinical characteristics in fact reflect differences in underlying biological processes. As for differences between cohorts, the BIOSTAT-CHF cohort used by Tromp et al.,^11^ though similar in demographics, included HFrEF patients that had a larger number of comorbidities and higher baseline NT-proBNP compared to the current study, and might therefore describe additional subphenotypes that emerged as the condition grew more severe.

When comparing our main analysis, which used the LME model coefficients (based on repeated measurements), with our additional analyses that used measurements from specific (baseline, second, last available) time points, we see overlapping results. Subphenotypes 1, 2, and 3 of all analyses are generally similar to each other regarding clinical characteristics. However, the survival model clearly shows a better fit for the survival model when using the mixed model subphenotypes, with a higher optimism corrected c-index (repeated measurements: 0.73, baseline measurement: 0.68, second measurement: 0.69, final measurement: 0.71) and a lower Bayesian information criterion (BIC; repeated measurements: 1023, baseline measurement: 1040, second measurement: 1037, final measurement: 1039). These results suggest that repeatedly measured biomarkers lead to more distinct subphenotypes regarding prognosis of HFrEF, and may therefore carry potential to provide more accurate pathophysiological insights.

The enhanced knowledge on HFrEF taxonomy as obtained in this study via dynamic proteomics-based deep phenotyping carries several potential clinical implications. It may contribute to further elucidation of biological mechanisms at work in HFrEF via retro-translation, enabling further tailored therapeutic approaches. An ensuing deeper understanding of the biological mechanisms of HFrEF could identify proteins that could serve as specific targets for therapy. Furthermore, knowledge on the proteomic and/or clinical characteristics that are most prominently associated with relevant subphenotypes, could enable clinical trial designs targeting therapeutics to more homogeneous groups, to improve probability of clinical benefit.

Limitations of this study include the limited number of women (27%) and patients with a non-Caucasian ethnicity (7%). Furthermore, compared with some other chronic HF cohorts, the proportion of HF patients in NYHA classes I and II were high in our study (73%), which may have obscured subphenotypes that may have more clearly emerged in patients with more severe illness. Additionally, the sample size of this study was somewhat limited compared to other phenotyping literature,^3^^,^^11^ however these previous studies did not use elaborate proteomics, and moreover we included much more information about the patients via the repeated measurements design (in total 1066 blood samples were used). This greatly enhances statistical power, thus obviating sample size concerns. Our study, as any, could have benefitted from external validation, however due to its unique serial blood sampling design, and repeated application of a large aptamer-based assay, this was challenging. Instead, we conducted extensive internal validation, incorporating the full clustering analysis workflow including the dimension reduction step, which showed that our subphenotypes are stable. The methodology used for protein measurement also warrants some consideration. The SOMAmer reagents were selected against proteins in their native folded conformations. Therefore, proteins that are unfolded or denatured are not detected. Furthermore, the SOMAscan assay returns RFUs instead of absolute concentrations. These values can be used to compare patients and changes over time within a patient. However, they are not recommended for use in clinical applications that require absolute concentration to inform treatment decisions.

In conclusion, in this study, we found four circulating-protein based HFrEF subphenotypes which were driven by different combinations of biomarker subsets that relate to varying biological mechanisms. These four subphenotypes had different clinical characteristics and different prognosis. Our findings suggest that repeated assessment of extensive biomarker panels may contribute to the further understanding of complex diagnoses like HF through dynamic phenotyping methods.

## Contributors

All authors have read and approved the manuscript. The first and corresponding authors have directly accessed and verified the data. The contributions of the authors are the following:

T.B.P. – conception and design of the work, analysis and interpretation of data, writing the manuscript; M.B. –the acquisition, analysis and interpretation of data, revising the manuscript; F.W.A. – conception and design of the work, interpretation of data, revising the manuscript, handling funding; M.H. – analysis and interpretation of data, revising the manuscript; K.M.A. – conception and design of the work, the acquisition and interpretation of data, revising the manuscript; J.J.B – the acquisition and interpretation of data, revising the manuscript; J.R. – the acquisition and interpretation of data, revising the manuscript; R.T.L –interpretation of data, revising the manuscript; R.O. – interpretation of data, revising the manuscript; P.D.K. – conception and design of the work, interpretation of data, revising the manuscript; P.J.S. – conception and design of the work, interpretation of data, revising the manuscript; V.A.U. – conception and design of the work, the acquisition and interpretation of data, revising the manuscript; E.B. – conception and design of the work, interpretation of data, revising the manuscript, handling funding; D.R. – conception and design of the work, analysis and interpretation of data, revising the manuscript, supervision; I.K. – conception and design of the work, the acquisition, analysis and interpretation of data, revising the manuscript, handling funding and supervision.

## Data sharing statement

Anonymized data that support the findings of this study will be made available to other researchers for the purposes of reproducing the results upon reasonable request and in accordance with a data-sharing agreement.

## Declaration of interests

Rachel Ostroff is an employee of Somalogic Inc. with stock options. Jasper J. Brugts has received speaker fees or honoraria for advisory boards from Abbott, Novartis, Astra Zeneca, Vifor and Bayer in the past three years. The other authors have no conflicts of interest to report.
