# Supplementary material for: HFrEF subphenotypes based on 4210 repeatedly measured circulating proteins are driven by different biological mechanisms
Source: eBioMedicine. 2023 Jun 14;93:104655. doi: 10.1016/j.ebiom.2023.104655 (PMC10279550; doi:10.1016/j.ebiom.2023.104655)
Supplement: Supplementary material [file mmc1.docx]

**Supplemental Material:
HFrEF subphenotypes based on 4210 repeatedly measured circulating proteins are driven by different biological mechanisms**

Teun B. Petersen, MSc^a,b^; Marie de Bakker, MSc^a^; Folkert W. Asselbergs, MD, PhD^c,d^; Magdalena Harakalova, MD, PhD^e,f^; K. Martijn Akkerhuis, MD, PhD^a^; Jasper J. Brugts, MD, PhD^a^; Jan van Ramshorst, MD, PhD^g^; R. Thomas Lumbers, MD, PhD^h^; Rachel M. Ostroff, PhD^i^; Peter D. Katsikis, MD, PhD^j^; Peter J. van der Spek, PhD^k^; Victor A. Umans, MD, PhD^g^; Eric Boersma, PhD^a^; Dimitris Rizopoulos, PhD^b^*; Isabella Kardys, MD, PhD^a^*

^a^ Department of Cardiology, Erasmus MC, University Medical Center Rotterdam, Doctor Molewaterplein 40, Rotterdam, the Netherlands.

^b^ Department of Biostatistics, Erasmus MC, University Medical Center Rotterdam, Doctor Molewaterplein 40, Rotterdam, the Netherlands.

^c^ Amsterdam University Medical Centers, Department of Cardiology, University of Amsterdam, Meibergdreef 9, Amsterdam, The Netherlands

^d^ Health Data Research UK and Institute of Health Informatics, University College London, Gower St, London, United Kingdom

^e^ Department of Cardiology, Division Heart and Lungs, University Medical Center Utrecht, University of Utrecht, Heidelberglaan 100, Utrecht, the Netherlands.

^f^ Regenerative Medicine Center Utrecht, University Medical Center Utrecht, University of Utrecht, Heidelberglaan 100, Utrecht, the Netherlands

^g^ Department of Cardiology, Northwest Clinics, Wilhelminalaan 12, Alkmaar, the Netherlands.

^h^ British Heart Foundation Research Accelerator, University College London, Gower St, London, UK; Institute of Health Informatics, University College London, Gower St, London, UK; Health Data Research UK London, University College London, Gower St, London, UK.

^i^ SomaLogic, Inc., 2945 Wilderness Pl, Boulder, United States.

^j^ Department of Immunology, Erasmus MC, University Medical Center Rotterdam, Doctor Molewaterplein 40, Rotterdam, the Netherlands.

^k^ Department of Pathology, Erasmus MC, University Medical Center Rotterdam, Doctor Molewaterplein 40, Rotterdam, the Netherlands.

* These authors contributed equally

Contents

[**Detailed methods** 5](#_Toc131180713)

[**Proteomic measurements** 5](#_Toc131180714)

[**Sample size estimation** 5](#_Toc131180715)

[**Estimation of Linear mixed effect coefficients** 5](#_Toc131180716)

[**UMAP Parameters** 5](#_Toc131180717)

[**Choice of Clustering method** 6](#_Toc131180718)

[**Selecting the number of clusters** 6](#_Toc131180719)

[**Cluster stability / Internal validation** 6](#_Toc131180720)

[**Supplemental Figures and Figure Legends** 7](#_Toc131180721)

[**Supplemental Figure 1: Histogram with the results of the bootstrap analysis of the NBclust package for the analysis using intercept and slope values** 7](#_Toc131180722)

[**Supplemental Figure 2: Histogram with the results of the bootstrap analysis of the NBclust package for the analysis using baseline values** 8](#_Toc131180723)

[**Supplemental Figure 3: Heatmap displaying protein levels across subphenotypes for the clusters found using Baseline values** 9](#_Toc131180724)

[**Supplemental Figure 4: Kaplan-Meier plot illustrating the differences in prognosis for the subphenotypes found using baseline values** 10](#_Toc131180725)

[**Supplemental Figure 5: Histogram with the results of the bootstrap analysis of the NBclust package for the analysis using second measurements** 11](#_Toc131180726)

[**Supplemental Figure 6: Heatmap displaying protein levels across subphenotypes for the clusters found using second measurements** 12](#_Toc131180727)

[**Supplemental Figure 7: Kaplan-Meier plot illustrating the differences in prognosis for the subphenotypes found using second measurements** 13](#_Toc131180728)

[**Supplemental Figure 8: Histogram with the results of the bootstrap analysis of the NBclust package for the analysis using last measurements before PEP or censoring** 14](#_Toc131180729)

[**Supplemental Figure 9: Heatmap displaying protein levels across subphenotypes for the clusters found using using last measurements before PEP or censoring** 15](#_Toc131180730)

[**Supplemental Figure 10: Kaplan-Meier plot illustrating the differences in prognosis for the subphenotypes found using last measurements before PEP or censoring** 16](#_Toc131180731)

[**Supplemental Tables and supporting information** 17](#_Toc131180732)

[**Supplemental Table 1: Analytic performance of the SOMAscan assay** 17](#_Toc131180733)

[**Supplemental Table 2 Associations between clinical characteristics and the primary endpoint** 18](#_Toc131180734)

[**Supplemental Table 3: Top 10 proteins most associated with subphenotypes per protein subset with B-H corrected p-values of Kruskal-Wallis tests** 19](#_Toc131180735)

[**Supplemental Table 4: B-H p-values of disease associations with protein subsets** 20](#_Toc131180736)

[**Supplemental Table 5: B-H p-values of biological process associations with protein subsets** 22](#_Toc131180737)

[**Supplemental Table 6: B-H p-values of cellular component associations with protein subsets** 43](#_Toc131180738)

[**Supplemental Table 7: Baseline characteristics according to clusters based on baseline values** 49](#_Toc131180739)

[**Supplemental Table 8: Survival analysis using clusters based on the baseline values** 50](#_Toc131180740)

[**Supplemental Table 9: Baseline characteristics according to clusters based on second measurements** 51](#_Toc131180741)

[**Supplemental Table 10: Survival analysis using clusters based on second measurements** 52](#_Toc131180742)

[**Supplemental Table 11: Baseline characteristics according to clusters based the last measurements before PEP or censoring** 53](#_Toc131180743)

[**Supplemental Table 12: Survival analysis using clusters based on the last measurements before PEP or censoring** 54](#_Toc131180744)

[**Supplemental references** 55](#_Toc131180745)

## **Detailed methods**

### **Proteomic measurements**

Plasma protein concentration levels were measured using the aptamer-based proteomic SOMAscan platform.^1^ SOMAscan uses single stranded DNA-based protein affinity reagents called SOMAmers (Slow Off-rate Modified Aptamers). SOMAmers bind to proteins with high specificity and affinity, and slow dissociation rates, minimizing nonspecific binding interactions. The readout of the SOMAscan assay is given in normalized relative fluorescent units (RFUs). These RFUs are directly proportional to the amount of target protein in the initial sample. Previous studies have reported high assay reproducibility and low technical variability of SOMAscan.^2, 3^

The standard processes for normalization, calibration, and quality control (QC) were followed as previously described in Williams et al.^4^ The following normalization and calibration factors were considered acceptable: hybridization control, intraplate median signal normalization and plate scale factors were expected to be between 0·4 and 2·5; the distribution of QC sample ratios was expected to fall for 85% of individual SOMAmer reagents in the total array between 0·8 and 1·2. SOMAmers outside these ranges were omitted in this study. Moreover, SOMAmers with non-human and/or not-validated targets were excluded from further analyses. Whenever multiple SOMAmer versions were present, those with the highest binding affinity were used. This selection resulted in 4210 out of the total 5284 modified aptamers being used in the current analyses. Individual sample quality was evaluated by comparing normalized median signal relative to the external reference standard, with an acceptable normalization scaling range of 0·4 - 2·5. In total, 1066 samples passed the quality-control criteria. Analytic performance of the Somalogic panel is provided in supplemental table 1

### **Sample size estimation**

Sample size calculations for the Bio-SHiFT study (total n=398) were based on the expected associations between repeated circulating protein biomarkers and the primary endpoint. The current investigation comprised 382 HFrEF patients, of whom 114 reached the primary end point. For baseline measurements, these numbers are sufficient to detect odds ratios around 2 for the upper quintile of a biomarker associated with the end point (α error .05, power of 80%) when comparing incident cases with noncases. For repeated measurements, power is further enhanced. Based on input parameters derived from the benchmark blood biomarker NT-proBNP, and using 500 simulations, we calculated that using 3 measurements per person, a difference in change of NT-proBNP level over time of 10 pmol/L per month can be demonstrated between cases and non-cases (Bonferroni corrected α-error: (0.05/4210), power: 87%). This difference is very small in clinical terms, demonstrating that the study has high statistical power.

### **Estimation of Linear mixed effect coefficients**

The trajectory of every protein over time for every patient was estimated using linear mixed effect (LME) models with the log-transformed protein levels as the dependent variables and time of measurement as the sole fixed and random regressor next to the intercept. For every such model, a likelihood ratio test was used to determine whether the use of an exponential covariance serial correlation structure improved the model fit. This resulted in two coefficients per patient for every protein: one intercept, which can be interpreted as a measurement error-free estimate of the protein level at baseline, and one slope parameter, which describes the magnitude and direction of change in protein level over time.

### **UMAP Parameters**

The dimensions of the biomarker profiles were reduced via Uniform Manifold Approximation and Projection (UMAP). UMAP is a non-linear dimension reduction, which can improve the accuracy of cluster analysis over more conventional dimension reduction techniques like principal component analysis (PCA).^5^ UMAP has some important parameters which can heavily influence the resulting projections. These need to be specified with care. Firstly, the number of neighbors that are taken into account when finding an optimal lower dimensional projection. This parameter can be seen as one balancing the bias and variance of the projection. Less neighbors allows for more flexibility and leads to more details to remain at the cost of more noise, while more neighbors emphasizes the global structures while smoothing out details. Secondly, the minimal distance that observations can be apart, and finally the number of components on which to project the data.

As recommended by the documentation of the main UMAP package for clustering purposes we have chosen number of neighbors as 30, which is on the high end, to emphasize global clusters over local details.^6^ Next, we set the minimum distance as close to zero as possible, this allows groups of similar patients to be as close to each other as possible. This is advantageous for our purposes, as densely packing observations is makes it easier for the k-means algorithm to find distinct clusters. We chose to retain 30 components, as this results in more than 10 observations per component. Changing any of these parameters did not alter our results.

### **Choice of Clustering method**

We chose to use k-means as our clustering method over other more flexible clustering methods (such as Gaussian mixture models or density based methods), to ensure more stable and generalizable clusters. K-means can be seen as a special case of Gaussian mixture models with equal variance in a diagonal covariance matrix. Imposing this restriction reduces the risk of overfitting and improves reproducibility, at a cost of possibly slightly less detailed clusters. On top of this, k-means offers computational advantages over other methods, which was useful in in our bootstrapped high dimensional analysis. Additionally, k-means currently has a greater representation in heart failure phenotyping literature, and this alignment makes comparison of results between studies easier.

### **Selecting the number of clusters**

To account for the stochastic nature of UMAP and the variability of the dataset, NbClust and the preceding dimension reduction were bootstrapped. The following steps were repeated 100 times: sample 382 observations from the full dataset with replacement, log-transform and scale all biomarkers, estimate a lower dimensional representation of the data using the UMAP package and finally run the NBClust package on this lower dimensional projection. Every NBClust-run calculated 26 indices designating an optimal number of clusters.

### **Cluster stability / Internal validation**

Internal validity was assessed by investigating the stability of the cluster allocation under the addition of noise. This was done by repeating the cluster analysis, including the dimension reduction, on datasets where 5% of patients are replaced by random noise and calculating the Jaccard similarity between these cluster allocations and the allocation using the full dataset.

## **Supplemental Figures and Figure Legends**

### **Supplemental Figure 1: Histogram with the results of the bootstrap analysis of the NBclust package for the analysis using intercept and slope values**


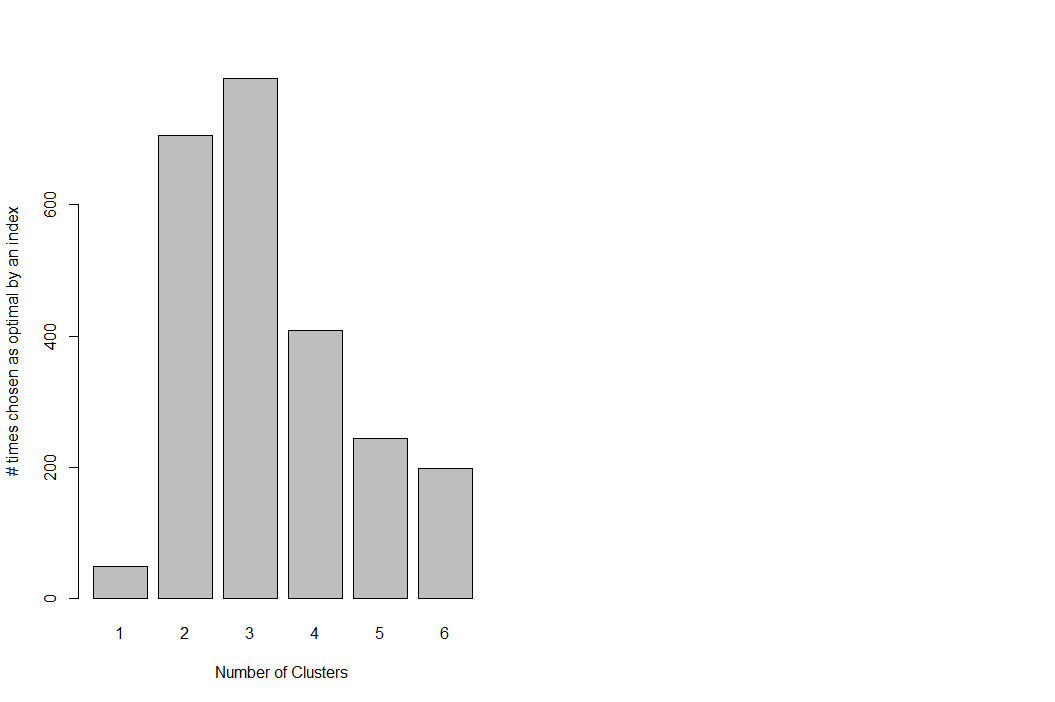


X-axis indicates number of clusters, the y-axis indicates the number of times a measure chose this number as optimal. Three clusters was chosen most often, followed by two and then four. Fitting three clusters resulted in suboptimal cluster stability. Fitting four clusters led to subphenotypes that showed more diversity in clinical characteristics and biomarker profiles than fitting just two clusters. Hence, four clusters was chosen as the best solution for our study. This approach led to stable and clinically relevant subphenotypes.

### **Supplemental Figure 2: Histogram with the results of the bootstrap analysis of the NBclust package for the analysis using baseline values**


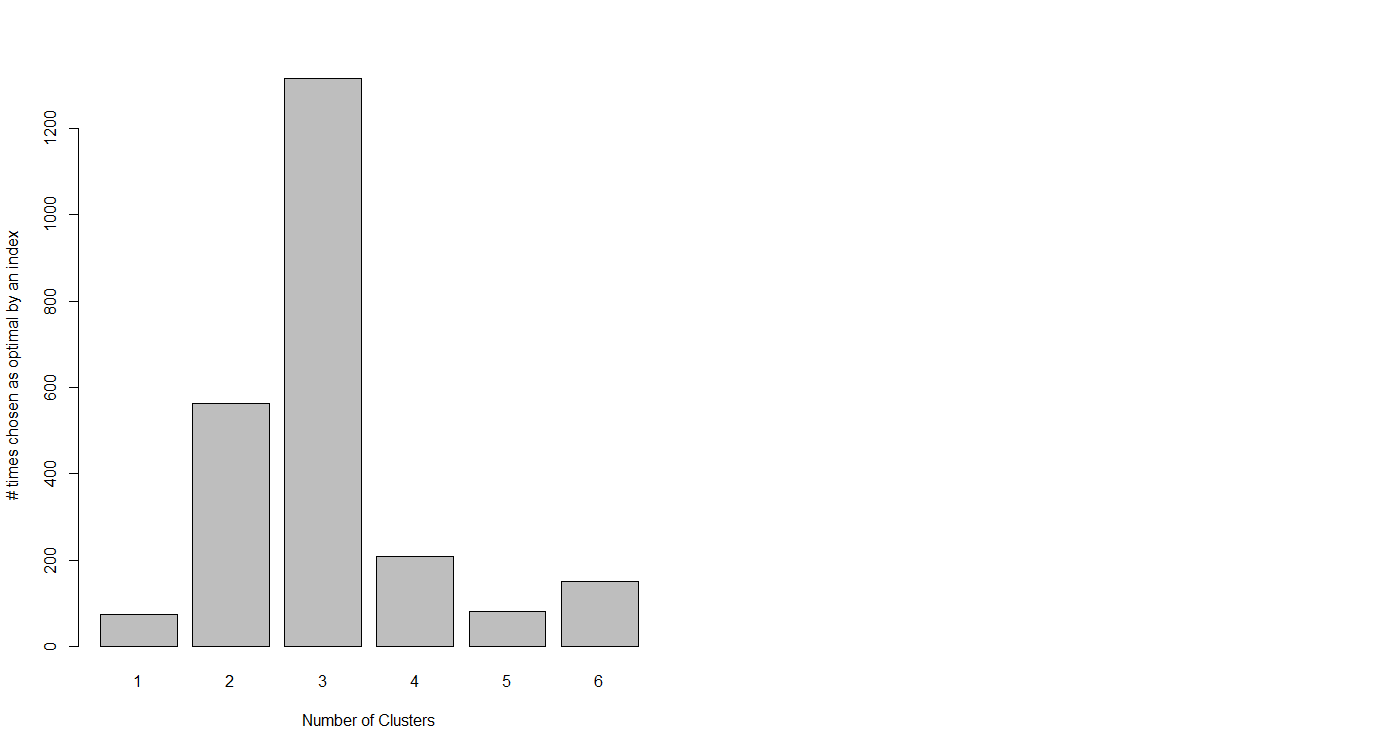


X-axis indicates number of clusters, the y-axis indicates the number of times a measure chose this number as optimal.

### **Supplemental Figure 3: Heatmap displaying protein levels across subphenotypes for the clusters found using Baseline values**


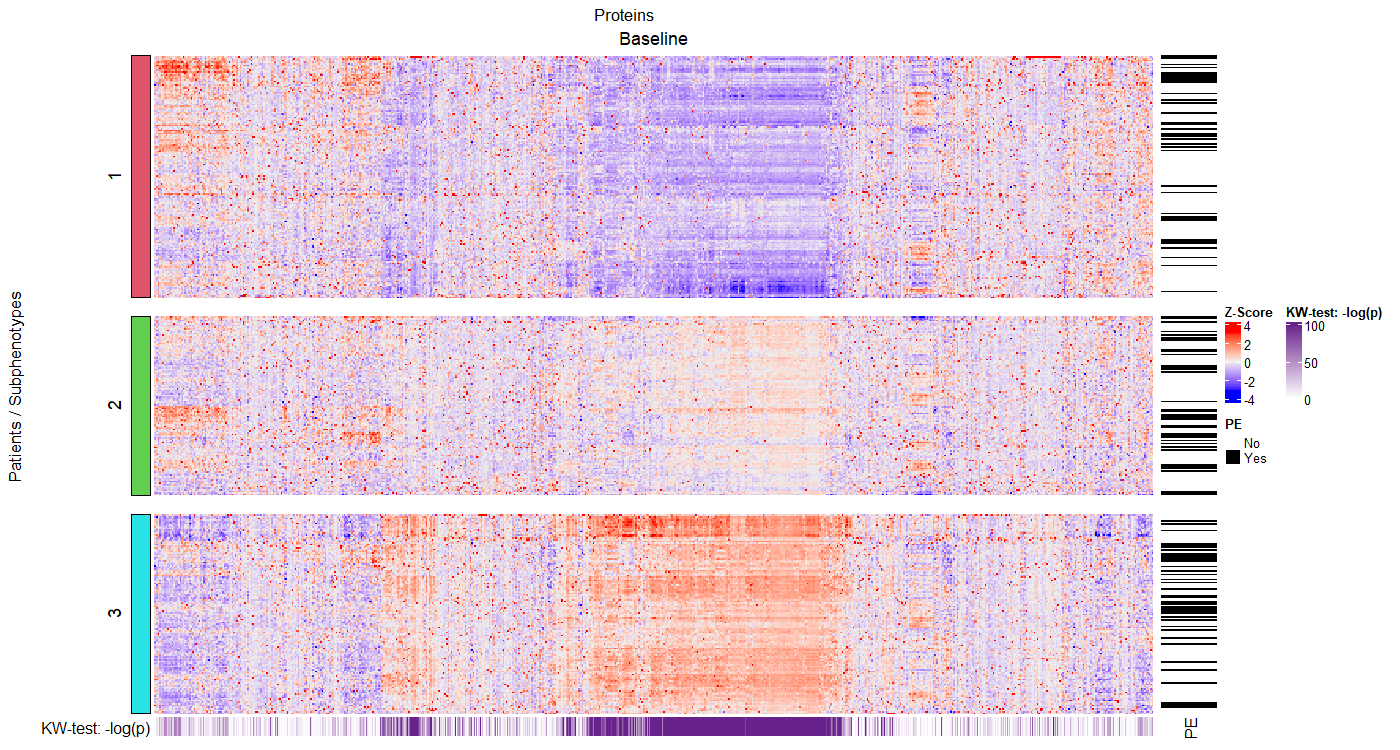


### **Supplemental Figure 4: Kaplan-Meier plot illustrating the differences in prognosis for the subphenotypes found using baseline values**


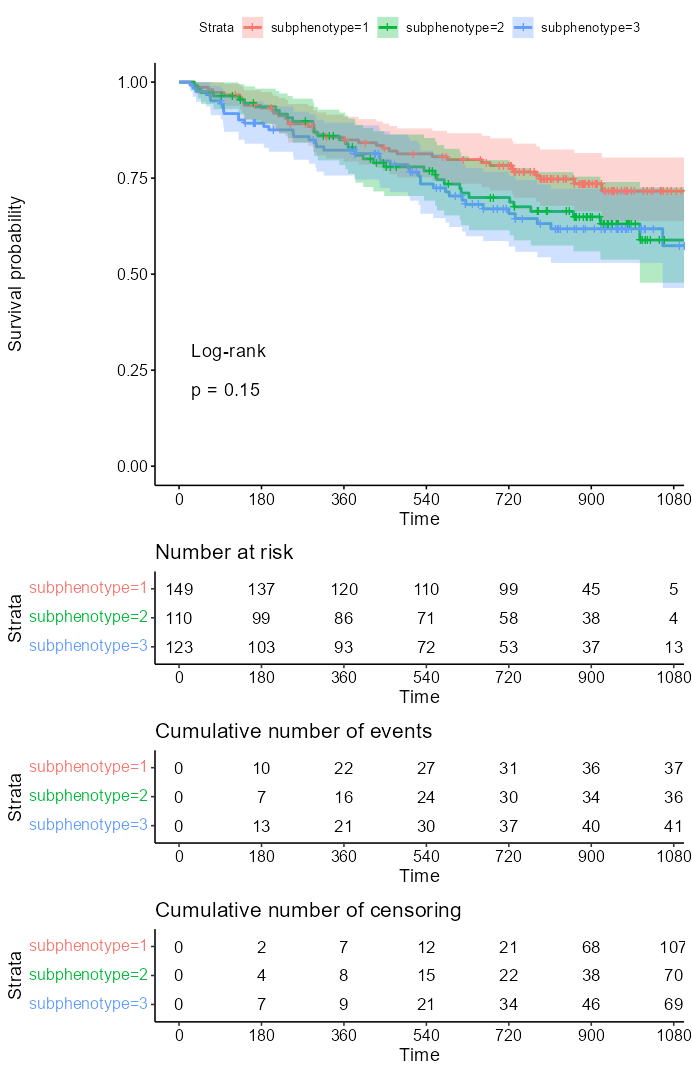


The primary endpoint is the composite of cardiovascular mortality, HF hospitalization, LVAD implantation and heart transplantation. The survival curves differ significantly from each other with a log-rank p-value of 0·042.

### **Supplemental Figure 5: Histogram with the results of the bootstrap analysis of the NBclust package for the analysis using second measurements**


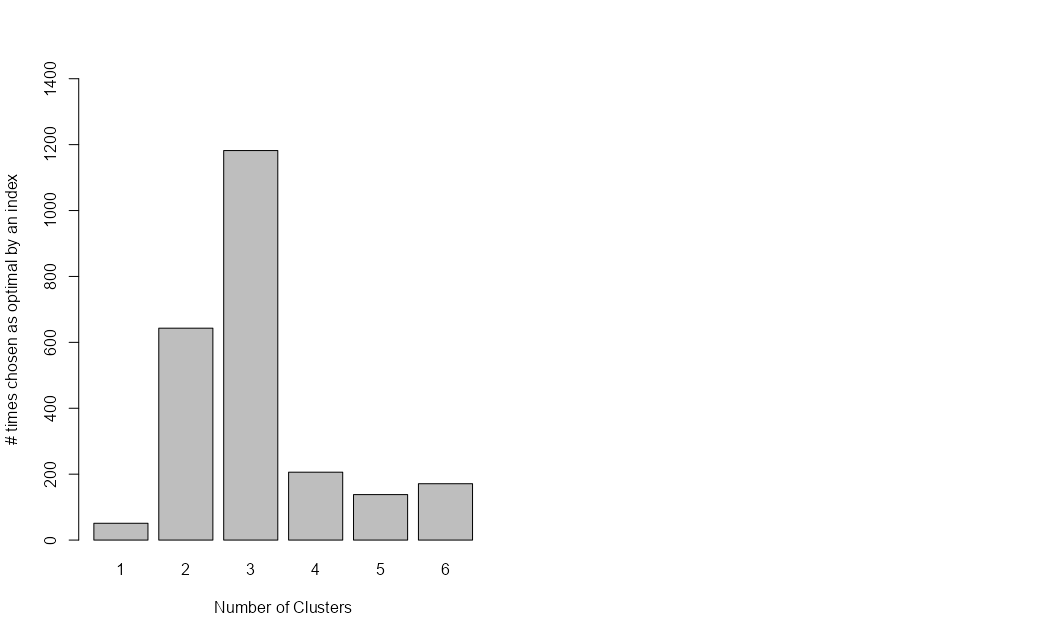


### **Supplemental Figure 6: Heatmap displaying protein levels across subphenotypes for the clusters found using second measurements**


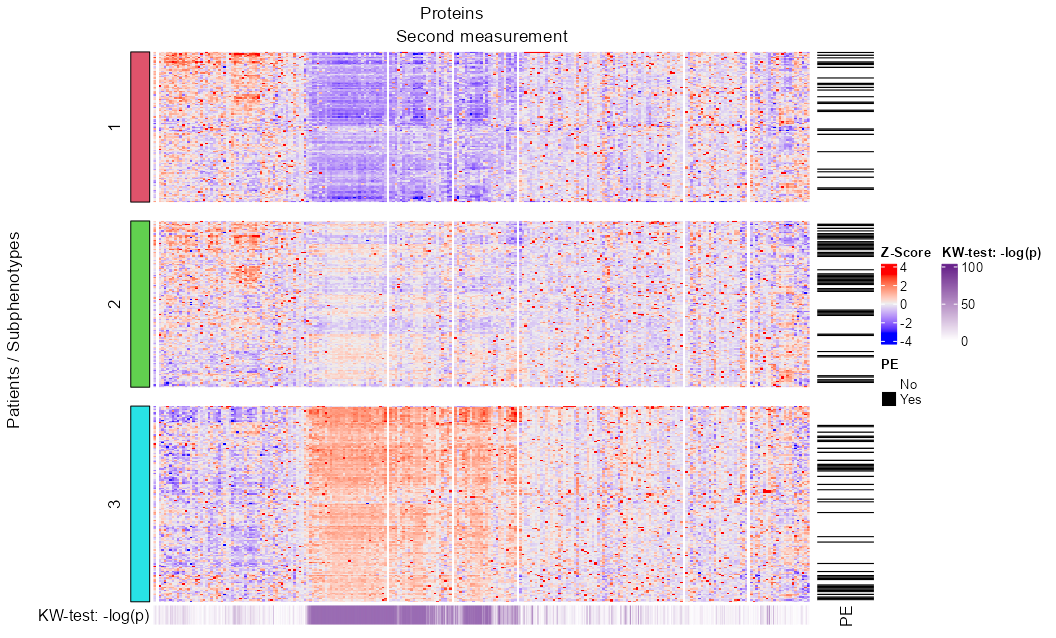


### **Supplemental Figure 7: Kaplan-Meier plot illustrating the differences in prognosis for the subphenotypes found using second measurements**


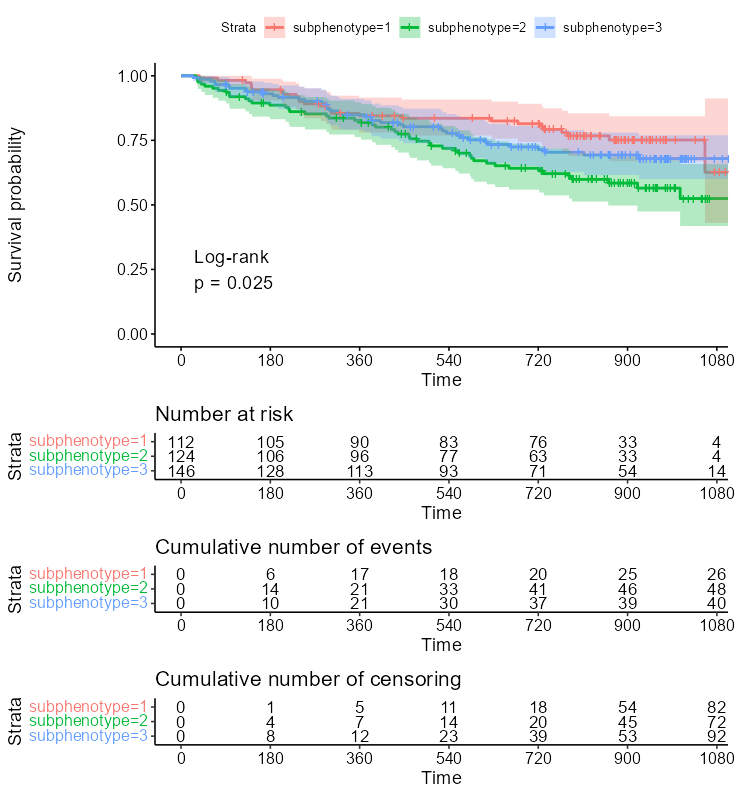


### **Supplemental Figure 8: Histogram with the results of the bootstrap analysis of the NBclust package for the analysis using last measurements before PEP or censoring**


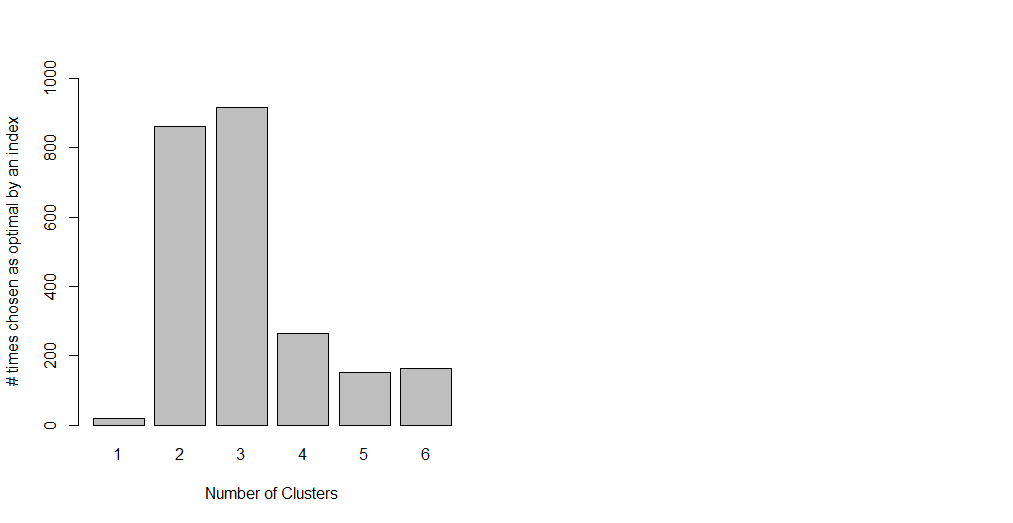


X-axis indicates number of clusters, the y-axis indicates the number of times a measure chose this number as optimal.

### **Supplemental Figure 9: Heatmap displaying protein levels across subphenotypes for the clusters found using using last measurements before PEP or censoring**


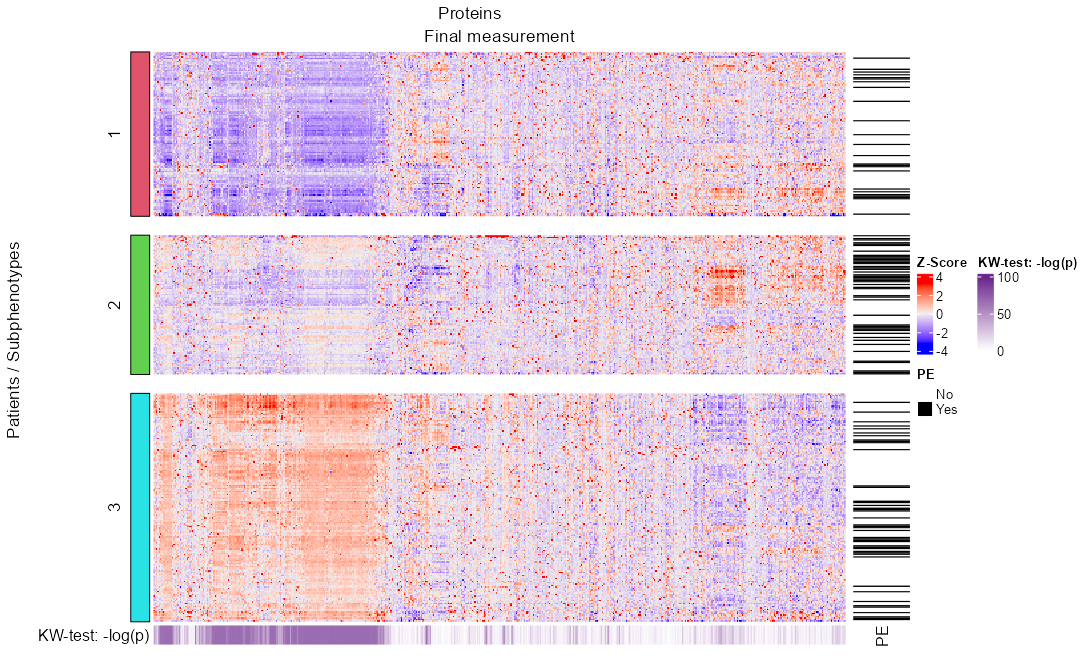


### **Supplemental Figure 10: Kaplan-Meier plot illustrating the differences in prognosis for the subphenotypes found using last measurements before PEP or censoring**


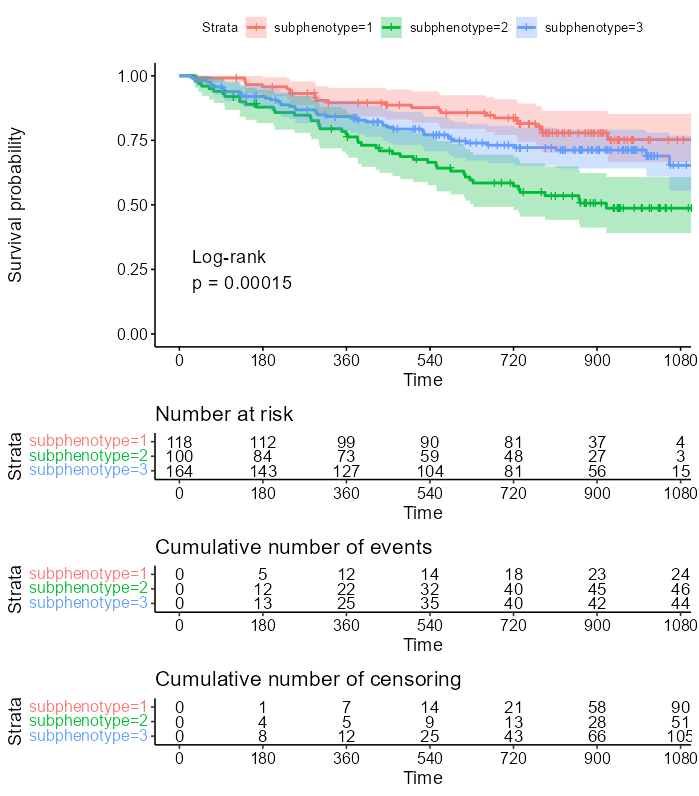


## **Supplemental Tables and supporting information**

### **Supplemental Table 1: Analytic performance of the SOMAscan assay**

| **Attribute** | **Metric** | **Criterion** | **Result** |
| --- | --- | --- | --- |
| Precision (Total CV) | Median %  % in Tail (above 90^th^ %) | ≤7.5%  <15% | 5.0%  11.8% |
| Accuracy | Median QC ratio  % in Tail | N/A  <15% | 1.0  2.4% |
| Signal to Noise | Median | ≥2.5 | 10.5 |
| Limit of Detection (LoD) | Median | ≤100 fM | 90fM |
| Spike and recovery | 25^th^-75^th^ percentile | N/A | 85%-121% |
| Median dynamic range per SOMAmer reagent buffer | Range | N/A | 4.7 logs |
| Interfering Substances | Non-Interfering | Pass | Pass |

### **Supplemental Table 2 Associations between clinical characteristics and the primary endpoint**

| Characteristic | Overall, N = 382^1^ | No PE, N = 268^1^ | PE, N = 114^1^ | p-value^2^ | q-value^3^ |
| --- | --- | --- | --- | --- | --- |
| **Demographics** |  |  |  |  |  |
| Age at baseline visit (years) | 64 (56, 72) | 64 (56, 72) | 64 (56, 75) | 0.5 | >0.9 |
| Gender: Male | 278 (73%) | 187 (70%) | 91 (80%) | **0.043** | >0.9 |
| Ethnicity: Caucasian | 351 (93%) | 246 (93%) | 105 (92%) | 0.8 | >0.9 |
| **Features of HF** |  |  |  |  |  |
| Duration (years) | 4.2 (1.6, 9.5) | 3.7 (1.2, 7.8) | 5.7 (2.6, 13.0) | **<0.001** | **0.011** |
| NYHA class I or II | 276 (73%) | 214 (80%) | 62 (55%) | **<0.001** | **<0.001** |
| Systolic ejection fraction (%) | 30 (23, 36) | 30 (23, 37) | 25 (20, 32) | **0.012** | 0.5 |
| **Clinical characteristics** |  |  |  |  |  |
| BMI (kg/m2) | 26.5 (24.0, 30.1) | 26.6 (24.2, 30.3) | 26.0 (23.3, 29.3) | 0.2 | >0.9 |
| eGFR CKD-EPI (mL/min/1.73m2) | 58 (42, 77) | 60 (44, 78) | 53 (39, 73) | 0.2 | >0.9 |
| Systolic blood pressure (mmHg) | 114 (100, 130) | 118 (104, 130) | 110 (93, 120) | **<0.001** | **0.003** |
| Diastolic blood pressure (mmHg) | 70 (60, 78) | 70 (61, 80) | 68 (60, 72) | **0.004** | 0.2 |
| **Biomarker level** |  |  |  |  |  |
| Nt-proBNP (pmol/L) | 133 (46, 274) | 94 (29, 201) | 297 (178, 519) | **<0.001** | **<0.001** |
| Hs-Troponin T (ng/L) | 18 (9, 33) | 14 (8, 27) | 32 (20, 48) | **<0.001** | **<0.001** |
| CRP (mg/L) | 2.2 (0.9, 4.9) | 1.7 (0.7, 4.2) | 2.8 (1.4, 5.2) | **0.015** | 0.7 |
| **Etiology of HF** |  |  |  |  |  |
| Ischemic heart disease | 166 (43%) | 113 (42%) | 53 (46%) | 0.4 | >0.9 |
| Hypertension | 33 (8.6%) | 25 (9.3%) | 8 (7.0%) | 0.5 | >0.9 |
| Secondary to valvular heart disease | 12 (3.1%) | 6 (2.2%) | 6 (5.3%) | 0.2 | >0.9 |
| Cardiomyopathy | 122 (32%) | 82 (31%) | 40 (35%) | 0.4 | >0.9 |
| Hypertrophic (HCM) | 15 (3.9%) | 6 (2.2%) | 9 (7.9%) | **0.017** | 0.8 |
| Dilated (DCM) | 97 (25%) | 68 (25%) | 29 (25%) | >0.9 | >0.9 |
| Restrictive | 0 (0%) | 0 (0%) | 0 (0%) | >0.9 | >0.9 |
| Arrhytmogenic right ventricular (ARVC) | 1 (0.3%) | 0 (0%) | 1 (0.9%) | 0.3 | >0.9 |
| Non compaction cardiomyopathy | 4 (1.0%) | 4 (1.5%) | 0 (0%) | 0.3 | >0.9 |
| Unclassified | 7 (1.8%) | 4 (1.5%) | 3 (2.6%) | 0.4 | >0.9 |
| Unknown | 27 (7.1%) | 24 (9.0%) | 3 (2.6%) | **0.027** | >0.9 |
| **Medical history** |  |  |  |  |  |
| Myocardial Infarction | 145 (38%) | 96 (37%) | 49 (43%) | 0.2 | >0.9 |
| PCI | 126 (33%) | 88 (33%) | 38 (33%) | >0.9 | >0.9 |
| CABG | 54 (14%) | 35 (13%) | 19 (17%) | 0.4 | >0.9 |
| atrial fibrillation | 137 (36%) | 80 (30%) | 57 (50%) | **<0.001** | **0.009** |
| other arrhythmia | 151 (40%) | 91 (34%) | 60 (53%) | **<0.001** | **0.027** |
| pacemaker implantation | 85 (23%) | 54 (21%) | 31 (29%) | 0.10 | >0.9 |
| ICD implantation | 254 (66%) | 166 (62%) | 88 (77%) | **0.004** | 0.2 |
| CRT | 113 (30%) | 74 (28%) | 39 (35%) | 0.2 | >0.9 |
| stroke (CVA/TIA) | 48 (13%) | 31 (12%) | 17 (15%) | 0.3 | >0.9 |
| chronic renal failure | 181 (48%) | 112 (42%) | 69 (61%) | **<0.001** | **0.029** |
| Diabetes Mellitus | 98 (26%) | 61 (23%) | 37 (32%) | **0.047** | >0.9 |
| Known hypercholesterolemia | 160 (43%) | 107 (41%) | 53 (47%) | 0.3 | >0.9 |
| Hypertension | 166 (44%) | 111 (42%) | 55 (48%) | 0.3 | >0.9 |
| **Intoxication** |  |  |  |  |  |
| Smoking: Ever | 271 (71%) | 186 (70%) | 85 (75%) | 0.3 | >0.9 |
| Smoking: Current | 343 (90%) | 239 (90%) | 104 (92%) | 0.4 | >0.9 |
| **Medication** |  |  |  |  |  |
| Ace Inhibitor | 258 (68%) | 187 (70%) | 71 (62%) | 0.14 | >0.9 |
| Angiotensin II receptor blockers | 107 (28%) | 75 (28%) | 32 (28%) | >0.9 | >0.9 |
| Aldosteron antagonists | 293 (77%) | 199 (74%) | 94 (82%) | 0.083 | >0.9 |
| Diuretics other | 5 (1.3%) | 4 (1.5%) | 1 (0.9%) | >0.9 | >0.9 |
| Beta blockers | 350 (92%) | 249 (93%) | 101 (89%) | 0.13 | >0.9 |
| Aspirin | 77 (20%) | 59 (22%) | 18 (16%) | 0.2 | >0.9 |
| ^1^Median (IQR); n (%) | | | | |  |
| ^2^Wilcoxon rank sum test; Pearson's Chi-squared test; Fisher's exact test  ^3^Bonferroni correction for multiple testing  BMI= Body mass index, PCI= Percutaneous coronary intervention, CABG= Coronary artery bypass surgery, ICD= implantable cardioverter-defibrillator, CRT= Cardiac resynchronisation therapy | | | | |  |

### **Supplemental Table 3: Top 10 proteins most associated with subphenotypes per protein subset with B-H corrected p-values of Kruskal-Wallis tests**

| **Intercept A** | **p-value** | **Intercept B** | **p-value** |
| --- | --- | --- | --- |
| Peptidyl-prolyl cis-trans isomerase F, mitochondrial | 7.01E-49 | Lysophospholipase-like protein 1 | 4.76E-39 |
| Tyrosine-protein kinase BTK | 6.08E-48 | Ubiquitin thioesterase OTUB2 | 5.41E-36 |
| Cyclin-dependent kinase 1:G2/mitotic-specific cyclin-B1 complex | 2.08E-45 | Bromodomain testis-specific protein | 1.85E-33 |
| Serine/threonine-protein kinase Nek7 | 2.73E-45 | Thymidine kinase 2, mitochondrial | 2.45E-32 |
| Thiamin pyrophosphokinase 1 | 3.83E-45 | Sphingosine kinase 2 | 1.49E-31 |
| Mucosal addressin cell adhesion molecule 1 | 5.78E-45 | Tyrosyl-DNA phosphodiesterase 1 | 2.64E-31 |
| Pleckstrin | 1.46E-43 | Coxsackievirus and adenovirus receptor | 6.44E-31 |
| Signal transducer and activator of transcription 3 | 9.49E-43 | DNA-directed DNA/RNA polymerase mu | 2.73E-30 |
| CUB and sushi domain-containing protein 1 | 9.83E-43 | Oxysterols receptor LXR-beta | 2.80E-30 |
| Hydroxyacyl-coenzyme A dehydrogenase, mitochondrial | 1.09E-42 | Relaxin-3 | 4.93E-30 |
| **Intercept C** | **p-value** | **Intercept D** | **p-value** |
| Zinc finger protein 334 | 9.92E-25 | Inactive tyrosine-protein kinase transmembrane receptor ROR1 | 4.38E-25 |
| UPF0577 protein KIAA1324 | 2.57E-17 | C1q-related factor | 2.31E-21 |
| C-type lectin domain family 2 member A | 6.77E-17 | Leucine-rich repeat transmembrane protein FLRT2 | 7.61E-21 |
| Kunitz-type protease inhibitor 1 | 7.66E-17 | ADAMTS-like protein 1 | 1.19E-20 |
| Papilin | 9.13E-16 | CXADR-like membrane protein | 1.51E-20 |
| VPS10 domain-containing receptor SorCS3 | 1.21E-15 | Tyrosine-protein kinase transmembrane receptor ROR2 | 6.41E-20 |
| Lactadherin | 1.80E-15 | Alpha-(1,3)-fucosyltransferase 5 | 2.22E-19 |
| Stabilin-1 | 3.90E-15 | Tryptase beta-1 | 1.62E-18 |
| Receptor tyrosine-protein kinase erbB-3 | 7.72E-15 | Leucine-rich repeat transmembrane neuronal protein 2 | 1.70E-18 |
| Ubiquitin-conjugating enzyme E2 R1 | 1.05E-14 | Collagen alpha-2(VI) chain | 2.42E-18 |
|  |  |  |  |
| **Slope A** | **p-value** | **Slope B** | **p-value** |
| EGF-like repeat and discoidin I-like domain-containing protein 3 | 2.44E-32 | Thiamin pyrophosphokinase 1 | 2.96E-46 |
| Lactadherin | 3.90E-32 | Relaxin-3 | 5.01E-45 |
| Inactive tyrosine-protein kinase transmembrane receptor ROR1 | 2.67E-30 | Cyclin-dependent kinase 1:G2/mitotic-specific cyclin-B1 complex | 8.32E-45 |
| 14-3-3 protein epsilon | 3.78E-30 | Bromodomain-containing protein 4 | 2.15E-44 |
| Growth arrest-specific protein 1 | 4.04E-29 | Arf-GAP with coiled-coil, ANK repeat and PH domain-containing protein 2 | 2.90E-44 |
| Neurexin-3-beta | 4.36E-29 | Cell cycle checkpoint protein RAD1 | 6.70E-44 |
| Neurexin-3 | 1.43E-28 | Succinate dehydrogenase assembly factor 2, mitochondrial | 3.02E-43 |
| Leucine-rich repeat transmembrane protein FLRT2 | 2.12E-28 | Tyrosyl-DNA phosphodiesterase 1 | 9.23E-43 |
| ADAMTS-like protein 1 | 4.70E-28 | Sphingosine kinase 2 | 1.74E-42 |
| Interleukin-18-binding protein | 7.13E-28 | Ubiquitin-conjugating enzyme E2 D2 | 2.13E-42 |
| **Slope C** | **p-value** |  |  |
| Protein disulfide-isomerase A6 | 2.69E-59 |  |  |
| Protein disulfide-isomerase A3 | 2.69E-59 |  |  |
| Ras-related protein Rab-1B | 2.69E-59 |  |  |
| Mitogen-activated protein kinase 14 | 2.69E-59 |  |  |
| Translationally-controlled tumor protein | 2.69E-59 |  |  |
| Hsp90alpha | 2.69E-59 |  |  |
| Ras-related protein Ral-A | 2.69E-59 |  |  |
| Alcohol dehydrogenase 6 | 2.69E-59 |  |  |
| Alpha-actinin-1 | 3.32E-59 |  |  |
| GTP-binding protein SAR1a | 3.32E-59 |  |  |

### **Supplemental Table 4: B-H p-values of disease associations with protein subsets**

| ID | Name | Intercept A | Intercept B | Intercept C | Intercept D | Slope A | Slope B | Slope C |
| --- | --- | --- | --- | --- | --- | --- | --- | --- |
| C0004134 | Ataxia | 3.10E-02 | 6.38E-01 | 9.99E-01 | 1.00E+00 | 9.40E-01 | 9.73E-01 | 2.35E-01 |
| C0007758 | Cerebellar Ataxia | 3.51E-02 | 9.39E-01 | 9.99E-01 | 1.00E+00 | 1.00E+00 | 9.75E-01 | 4.56E-01 |
| C0263630 | Hypertrophic disorder of skin, unspecified | 3.51E-02 | 9.75E-01 | 1.00E+00 | 9.70E-01 | 9.95E-01 | 1.00E+00 | 1.22E-01 |
| C0029408 | Degenerative polyarthritis | 9.17E-01 | 8.28E-01 | 9.99E-01 | 3.51E-04 | 1.73E-01 | 9.98E-01 | 9.16E-01 |
| C0011615 | Dermatitis, Atopic | 9.17E-01 | 7.37E-01 | 9.99E-01 | 4.79E-04 | 1.73E-01 | 9.73E-01 | 9.16E-01 |
| C0018801 | Heart failure | 9.17E-01 | 8.56E-01 | 9.99E-01 | 5.79E-04 | 9.02E-01 | 9.84E-01 | 9.16E-01 |
| C0011881 | Diabetic Nephropathy | 9.17E-01 | 6.38E-01 | 9.99E-01 | 1.23E-03 | 9.02E-01 | 9.73E-01 | 9.16E-01 |
| C0018802 | Congestive heart failure | 9.17E-01 | 8.56E-01 | 9.99E-01 | 1.25E-03 | 9.02E-01 | 9.84E-01 | 9.16E-01 |
| C0153676 | Secondary malignant neoplasm of lung | 9.17E-01 | 9.53E-01 | 1.00E+00 | 6.16E-03 | 9.02E-01 | 1.00E+00 | 9.16E-01 |
| C0024117 | Chronic Obstructive Airway Disease | 9.17E-01 | 6.38E-01 | 1.00E+00 | 6.16E-03 | 9.02E-01 | 9.73E-01 | 9.16E-01 |
| C0004096 | Asthma | 9.17E-01 | 6.38E-01 | 9.99E-01 | 9.51E-03 | 1.73E-01 | 9.85E-01 | 9.16E-01 |
| C0023890 | Liver Cirrhosis | 9.17E-01 | 8.87E-01 | 9.99E-01 | 1.27E-02 | 3.91E-01 | 9.78E-01 | 9.16E-01 |
| C0041296 | Tuberculosis | 9.17E-01 | 7.92E-01 | 9.99E-01 | 1.77E-02 | 9.02E-01 | 9.73E-01 | 9.16E-01 |
| C0036202 | Sarcoidosis | 9.17E-01 | 7.37E-01 | 9.99E-01 | 2.15E-02 | 9.01E-01 | 9.73E-01 | 9.16E-01 |
| C0948089 | Acute Coronary Syndrome | 9.17E-01 | 6.38E-01 | 9.99E-01 | 2.51E-02 | 9.02E-01 | 9.73E-01 | 9.16E-01 |
| C2316810 | Chronic kidney disease stage 5 | 9.17E-01 | 7.35E-01 | 9.99E-01 | 4.31E-02 | 9.02E-01 | 9.73E-01 | 9.16E-01 |
| C0010346 | Crohn Disease | 9.17E-01 | 6.72E-01 | 9.99E-01 | 4.59E-02 | 9.02E-01 | 9.73E-01 | 9.16E-01 |
| C0019829 | Hodgkin Disease | 9.17E-01 | 6.72E-01 | 9.99E-01 | 4.75E-02 | 8.45E-01 | 9.73E-01 | 9.16E-01 |
| C0036690 | Septicemia | 9.46E-01 | 8.13E-01 | 9.99E-01 | 2.39E-02 | 9.02E-01 | 9.73E-01 | 9.41E-01 |
| C0009319 | Colitis | 9.69E-01 | 8.74E-01 | 9.99E-01 | 4.97E-03 | 7.26E-01 | 9.73E-01 | 9.69E-01 |
| C1623038 | Cirrhosis | 9.69E-01 | 6.72E-01 | 9.99E-01 | 9.51E-03 | 3.91E-01 | 9.77E-01 | 9.16E-01 |
| C0206754 | Neuroendocrine Tumors | 9.69E-01 | 9.02E-01 | 9.99E-01 | 4.03E-02 | 6.49E-01 | 9.73E-01 | 9.41E-01 |
| C0038454 | Cerebrovascular accident | 9.75E-01 | 8.82E-01 | 9.99E-01 | 4.23E-03 | 6.22E-01 | 9.73E-01 | 9.41E-01 |
| C0034069 | Pulmonary Fibrosis | 9.99E-01 | 8.27E-01 | 9.99E-01 | 1.53E-02 | 9.02E-01 | 9.90E-01 | 9.16E-01 |
| C0003864 | Arthritis | 9.99E-01 | 9.42E-01 | 9.99E-01 | 2.60E-02 | 9.02E-01 | 9.73E-01 | 9.69E-01 |
| C0400966 | Non-alcoholic Fatty Liver Disease | 9.99E-01 | 6.38E-01 | 9.99E-01 | 2.65E-02 | 9.02E-01 | 9.73E-01 | 9.41E-01 |
| C1800706 | Idiopathic Pulmonary Fibrosis | 9.99E-01 | 7.92E-01 | 9.99E-01 | 3.30E-02 | 9.02E-01 | 9.73E-01 | 9.41E-01 |
| C0004153 | Atherosclerosis | 1.00E+00 | 6.72E-01 | 9.99E-01 | 3.25E-06 | 5.37E-01 | 9.73E-01 | 9.98E-01 |
| C0003850 | Arteriosclerosis | 1.00E+00 | 7.92E-01 | 9.99E-01 | 4.24E-06 | 6.49E-01 | 9.73E-01 | 1.00E+00 |
| C0036421 | Systemic Scleroderma | 1.00E+00 | 6.38E-01 | 9.99E-01 | 4.24E-06 | 3.91E-01 | 9.73E-01 | 9.41E-01 |
| C0013595 | Eczema | 1.00E+00 | 8.16E-01 | 9.99E-01 | 6.43E-05 | 1.73E-01 | 9.73E-01 | 1.00E+00 |
| C0010068 | Coronary heart disease | 1.00E+00 | 6.72E-01 | 9.99E-01 | 7.22E-05 | 3.78E-01 | 9.73E-01 | 1.00E+00 |
| C0014175 | Endometriosis | 1.00E+00 | 6.38E-01 | 9.99E-01 | 9.17E-05 | 6.69E-01 | 9.73E-01 | 9.69E-01 |
| C1956346 | Coronary Artery Disease | 1.00E+00 | 6.38E-01 | 9.99E-01 | 1.19E-04 | 2.96E-01 | 9.73E-01 | 1.00E+00 |
| C0010054 | Coronary Arteriosclerosis | 1.00E+00 | 6.38E-01 | 9.99E-01 | 2.30E-04 | 4.48E-01 | 9.73E-01 | 1.00E+00 |
| C1519670 | Tumor Angiogenesis | 1.00E+00 | 8.39E-01 | 1.00E+00 | 4.88E-04 | 3.98E-01 | 9.99E-01 | 9.98E-01 |
| C0021390 | Inflammatory Bowel Diseases | 1.00E+00 | 6.72E-01 | 9.99E-01 | 1.02E-03 | 1.87E-01 | 9.78E-01 | 9.16E-01 |
| C0007222 | Cardiovascular Diseases | 1.00E+00 | 6.72E-01 | 9.99E-01 | 1.76E-03 | 7.26E-01 | 9.73E-01 | 1.00E+00 |
| C1561643 | Chronic Kidney Diseases | 1.00E+00 | 6.38E-01 | 9.99E-01 | 2.49E-03 | 5.53E-01 | 9.73E-01 | 9.86E-01 |
| C4048329 | Immunosuppression | 1.00E+00 | 8.62E-01 | 9.99E-01 | 2.76E-03 | 9.02E-01 | 9.73E-01 | 1.00E+00 |
| C1658953 | tumor vasculature | 1.00E+00 | 9.52E-01 | 1.00E+00 | 2.76E-03 | 2.54E-01 | 1.00E+00 | 9.86E-01 |
| C0030193 | Pain | 1.00E+00 | 7.70E-01 | 9.99E-01 | 2.96E-03 | 3.98E-01 | 9.78E-01 | 9.87E-01 |
| C0033860 | Psoriasis | 1.00E+00 | 6.38E-01 | 9.99E-01 | 2.96E-03 | 3.91E-01 | 9.73E-01 | 9.16E-01 |
| C0027051 | Myocardial Infarction | 1.00E+00 | 6.62E-01 | 9.99E-01 | 3.59E-03 | 4.48E-01 | 9.73E-01 | 1.00E+00 |
| C0742343 | Acute Chest Syndrome | 1.00E+00 | 6.38E-01 | 9.99E-01 | 4.97E-03 | 9.01E-01 | 9.73E-01 | 1.00E+00 |
| C0022104 | Irritable Bowel Syndrome | 1.00E+00 | 7.37E-01 | 9.99E-01 | 4.97E-03 | 3.98E-01 | 9.73E-01 | 9.41E-01 |
| C0032285 | Pneumonia | 1.00E+00 | 8.32E-01 | 9.99E-01 | 6.16E-03 | 9.01E-01 | 9.73E-01 | 1.00E+00 |
| C1565489 | Renal Insufficiency | 1.00E+00 | 8.87E-01 | 9.99E-01 | 6.16E-03 | 8.86E-01 | 9.73E-01 | 1.00E+00 |
| C0031090 | Periodontal Diseases | 1.00E+00 | 6.38E-01 | 9.99E-01 | 6.16E-03 | 9.02E-01 | 9.73E-01 | 1.00E+00 |
| C0264714 | Acute heart failure | 1.00E+00 | 9.23E-01 | 9.99E-01 | 6.16E-03 | 9.02E-01 | 9.73E-01 | 1.00E+00 |
| C0022658 | Kidney Diseases | 1.00E+00 | 7.37E-01 | 9.99E-01 | 7.25E-03 | 9.02E-01 | 9.73E-01 | 9.86E-01 |
| C0281361 | Adenocarcinoma of pancreas | 1.00E+00 | 9.53E-01 | 9.99E-01 | 8.88E-03 | 7.68E-01 | 9.98E-01 | 9.86E-01 |
| C1536220 | ST segment elevation myocardial infarction | 1.00E+00 | 8.62E-01 | 9.99E-01 | 8.88E-03 | 3.42E-01 | 9.73E-01 | 1.00E+00 |
| C0014038 | Encephalitis | 1.00E+00 | 7.93E-01 | 9.99E-01 | 1.13E-02 | 9.02E-01 | 9.73E-01 | 1.00E+00 |
| C0162871 | Aortic Aneurysm, Abdominal | 1.00E+00 | 6.38E-01 | 9.99E-01 | 1.38E-02 | 9.02E-01 | 9.73E-01 | 1.00E+00 |
| C0014070 | Encephalomyelitis | 1.00E+00 | 8.18E-01 | 1.00E+00 | 1.49E-02 | 9.02E-01 | 9.89E-01 | 9.86E-01 |
| C2945695 | Limb ischemia | 1.00E+00 | 8.62E-01 | 1.00E+00 | 1.49E-02 | 7.26E-01 | 9.96E-01 | 9.86E-01 |
| C0039103 | Synovitis | 1.00E+00 | 7.64E-01 | 9.99E-01 | 1.77E-02 | 9.02E-01 | 9.73E-01 | 1.00E+00 |
| C0409959 | Osteoarthritis, Knee | 1.00E+00 | 7.92E-01 | 9.99E-01 | 1.80E-02 | 1.73E-01 | 9.73E-01 | 1.00E+00 |
| C0494165 | Secondary malignant neoplasm of liver | 1.00E+00 | 6.38E-01 | 1.00E+00 | 1.98E-02 | 9.02E-01 | 9.78E-01 | 1.00E+00 |
| C0037284 | Skin lesion | 1.00E+00 | 9.69E-01 | 9.99E-01 | 1.98E-02 | 9.02E-01 | 9.84E-01 | 1.00E+00 |
| C1704436 | Peripheral Arterial Diseases | 1.00E+00 | 7.57E-01 | 9.99E-01 | 2.03E-02 | 9.01E-01 | 9.73E-01 | 1.00E+00 |
| C0149925 | Small cell carcinoma of lung | 1.00E+00 | 6.38E-01 | 9.99E-01 | 2.15E-02 | 9.01E-01 | 9.73E-01 | 9.86E-01 |
| C0242383 | Age related macular degeneration | 1.00E+00 | 6.38E-01 | 9.99E-01 | 2.15E-02 | 9.02E-01 | 9.73E-01 | 1.00E+00 |
| C0206062 | Lung Diseases, Interstitial | 1.00E+00 | 6.38E-01 | 1.00E+00 | 2.15E-02 | 9.02E-01 | 9.73E-01 | 9.76E-01 |
| C0026769 | Multiple Sclerosis | 1.00E+00 | 6.38E-01 | 9.99E-01 | 2.39E-02 | 2.47E-01 | 9.73E-01 | 1.00E+00 |
| C0151517 | Complete atrioventricular block | 1.00E+00 | 6.38E-01 | 9.99E-01 | 2.72E-02 | 9.02E-01 | 9.73E-01 | 1.00E+00 |
| C0007785 | Cerebral Infarction | 1.00E+00 | 6.38E-01 | 9.99E-01 | 3.01E-02 | 9.02E-01 | 9.73E-01 | 9.41E-01 |
| C0521170 | Osteoporotic Fractures | 1.00E+00 | 7.37E-01 | 9.99E-01 | 3.08E-02 | 6.17E-01 | 9.73E-01 | 1.00E+00 |
| C4025272 | Peripheral arterial stenosis | 1.00E+00 | 8.40E-01 | 9.99E-01 | 3.21E-02 | 3.13E-01 | 9.90E-01 | 1.00E+00 |
| C0024115 | Lung diseases | 1.00E+00 | 6.38E-01 | 9.99E-01 | 3.40E-02 | 9.02E-01 | 9.73E-01 | 1.00E+00 |
| C3714636 | Pneumonitis | 1.00E+00 | 8.63E-01 | 9.99E-01 | 3.61E-02 | 9.01E-01 | 9.73E-01 | 1.00E+00 |
| C0024143 | Lupus Nephritis | 1.00E+00 | 7.92E-01 | 9.99E-01 | 3.61E-02 | 9.02E-01 | 9.73E-01 | 1.00E+00 |
| C4524092 | Chronic rhinosinusitis with nasal polyps | 1.00E+00 | 6.38E-01 | 9.99E-01 | 3.61E-02 | 7.29E-01 | 9.73E-01 | 1.00E+00 |
| C0162809 | Kallmann Syndrome | 1.00E+00 | 6.38E-01 | 9.99E-01 | 3.61E-02 | 4.51E-01 | 9.73E-01 | 1.00E+00 |
| C1619727 | Decompensated cirrhosis of liver | 1.00E+00 | 9.93E-01 | 9.99E-01 | 3.61E-02 | 2.63E-01 | 9.84E-01 | 1.00E+00 |
| C1262091 | Lymphocytic infiltration | 1.00E+00 | 7.37E-01 | 9.99E-01 | 3.85E-02 | 9.02E-01 | 9.73E-01 | 1.00E+00 |
| C0027796 | Neuralgia | 1.00E+00 | 6.38E-01 | 9.99E-01 | 4.05E-02 | 8.42E-01 | 9.73E-01 | 9.41E-01 |
| C0009404 | Colorectal Neoplasms | 1.00E+00 | 6.38E-01 | 9.99E-01 | 4.05E-02 | 4.48E-01 | 9.73E-01 | 1.00E+00 |
| C0007766 | Intracranial Aneurysm | 1.00E+00 | 9.39E-01 | 9.99E-01 | 4.31E-02 | 4.32E-01 | 9.73E-01 | 1.00E+00 |

### **Supplemental Table 5: B-H p-values of biological process associations with protein subsets**

| ID | Name | Intercept.A | Intercept.B | | Intercept.C | | Intercept.D | | Slope.A | | Slope.B | | Slope.C | |
| --- | --- | --- | --- | --- | --- | --- | --- | --- | --- | --- | --- | --- | --- | --- |
| GO:0046907 | intracellular transport | 8.79E-15 | 9.84E-01 | 1.00E+00 | | 1.00E+00 | | 1.00E+00 | | 1.00E+00 | | 1.50E-12 | |  |
| GO:0006412 | translation | 1.43E-14 | 9.33E-01 | 1.00E+00 | | 1.00E+00 | | 1.00E+00 | | 1.00E+00 | | 1.85E-12 | |  |
| GO:0043043 | peptide biosynthetic process | 1.43E-14 | 9.62E-01 | 1.00E+00 | | 1.00E+00 | | 1.00E+00 | | 1.00E+00 | | 2.02E-12 | |  |
| GO:0006886 | intracellular protein transport | 1.59E-13 | 9.84E-01 | 1.00E+00 | | 1.00E+00 | | 1.00E+00 | | 1.00E+00 | | 1.30E-11 | |  |
| GO:0006518 | peptide metabolic process | 2.31E-11 | 9.39E-01 | 1.00E+00 | | 1.00E+00 | | 1.00E+00 | | 1.00E+00 | | 1.06E-10 | |  |
| GO:0043604 | amide biosynthetic process | 2.70E-11 | 9.29E-01 | 1.00E+00 | | 1.00E+00 | | 1.00E+00 | | 1.00E+00 | | 8.21E-11 | |  |
| GO:0043603 | cellular amide metabolic process | 2.03E-10 | 9.29E-01 | 1.00E+00 | | 1.00E+00 | | 1.00E+00 | | 1.00E+00 | | 1.30E-11 | |  |
| GO:0045184 | establishment of protein localization | 3.06E-10 | 9.90E-01 | 1.00E+00 | | 1.00E+00 | | 1.00E+00 | | 1.00E+00 | | 1.12E-09 | |  |
| GO:0044265 | cellular macromolecule catabolic process | 4.45E-10 | 9.33E-01 | 1.00E+00 | | 1.00E+00 | | 1.00E+00 | | 1.00E+00 | | 3.96E-08 | |  |
| GO:0015031 | protein transport | 4.45E-10 | 9.90E-01 | 1.00E+00 | | 1.00E+00 | | 1.00E+00 | | 1.00E+00 | | 2.48E-09 | |  |
| GO:0055086 | nucleobase-containing small molecule metabolic process | 6.28E-10 | 9.35E-01 | 1.00E+00 | | 1.00E+00 | | 1.00E+00 | | 1.00E+00 | | 2.48E-09 | |  |
| GO:0009894 | regulation of catabolic process | 6.28E-10 | 9.45E-01 | 1.00E+00 | | 1.00E+00 | | 1.00E+00 | | 1.00E+00 | | 4.10E-09 | |  |
| GO:0010608 | post-transcriptional regulation of gene expression | 8.59E-10 | 9.33E-01 | 1.00E+00 | | 1.00E+00 | | 1.00E+00 | | 1.00E+00 | | 1.07E-09 | |  |
| GO:0009117 | nucleotide metabolic process | 8.59E-10 | 9.62E-01 | 1.00E+00 | | 1.00E+00 | | 1.00E+00 | | 1.00E+00 | | 1.31E-08 | |  |
| GO:0033365 | protein localization to organelle | 1.78E-09 | 9.74E-01 | 1.00E+00 | | 1.00E+00 | | 1.00E+00 | | 1.00E+00 | | 1.39E-08 | |  |
| GO:0006753 | nucleoside phosphate metabolic process | 2.82E-09 | 9.39E-01 | 1.00E+00 | | 1.00E+00 | | 1.00E+00 | | 1.00E+00 | | 4.48E-08 | |  |
| GO:0007010 | cytoskeleton organization | 1.13E-08 | 9.29E-01 | 1.00E+00 | | 1.00E+00 | | 1.00E+00 | | 1.00E+00 | | 1.28E-09 | |  |
| GO:0046390 | ribose phosphate biosynthetic process | 4.29E-08 | 9.66E-01 | 1.00E+00 | | 1.00E+00 | | 1.00E+00 | | 1.00E+00 | | 2.24E-06 | |  |
| GO:0019693 | ribose phosphate metabolic process | 6.91E-08 | 9.35E-01 | 1.00E+00 | | 1.00E+00 | | 1.00E+00 | | 1.00E+00 | | 2.92E-06 | |  |
| GO:0031329 | regulation of cellular catabolic process | 9.59E-08 | 9.39E-01 | 1.00E+00 | | 1.00E+00 | | 1.00E+00 | | 1.00E+00 | | 2.83E-07 | |  |
| GO:0016236 | macroautophagy | 1.05E-07 | 9.29E-01 | 1.00E+00 | | 1.00E+00 | | 1.00E+00 | | 1.00E+00 | | 4.59E-09 | |  |
| GO:0051603 | proteolysis involved in protein catabolic process | 1.16E-07 | 9.62E-01 | 1.00E+00 | | 1.00E+00 | | 1.00E+00 | | 1.00E+00 | | 3.90E-05 | |  |
| GO:0006417 | regulation of translation | 1.17E-07 | 9.29E-01 | 1.00E+00 | | 1.00E+00 | | 1.00E+00 | | 1.00E+00 | | 9.50E-08 | |  |
| GO:2000112 | regulation of cellular macromolecule biosynthetic process | 1.75E-07 | 9.29E-01 | 1.00E+00 | | 1.00E+00 | | 1.00E+00 | | 1.00E+00 | | 2.36E-07 | |  |
| GO:0043632 | modification-dependent macromolecule catabolic process | 1.97E-07 | 9.33E-01 | 1.00E+00 | | 1.00E+00 | | 1.00E+00 | | 1.00E+00 | | 3.00E-05 | |  |
| GO:0019941 | modification-dependent protein catabolic process | 2.58E-07 | 9.29E-01 | 1.00E+00 | | 1.00E+00 | | 1.00E+00 | | 1.00E+00 | | 3.36E-05 | |  |
| GO:0019637 | organophosphate metabolic process | 2.91E-07 | 9.29E-01 | 1.00E+00 | | 1.00E+00 | | 1.00E+00 | | 1.00E+00 | | 2.98E-06 | |  |
| GO:0090407 | organophosphate biosynthetic process | 6.22E-07 | 9.29E-01 | 1.00E+00 | | 1.00E+00 | | 1.00E+00 | | 1.00E+00 | | 9.35E-06 | |  |
| GO:0009259 | ribonucleotide metabolic process | 6.22E-07 | 9.39E-01 | 1.00E+00 | | 1.00E+00 | | 1.00E+00 | | 1.00E+00 | | 1.85E-05 | |  |
| GO:0034248 | regulation of cellular amide metabolic process | 1.10E-06 | 9.29E-01 | 1.00E+00 | | 1.00E+00 | | 1.00E+00 | | 1.00E+00 | | 1.42E-06 | |  |
| GO:0006163 | purine nucleotide metabolic process | 1.10E-06 | 9.33E-01 | 1.00E+00 | | 1.00E+00 | | 1.00E+00 | | 1.00E+00 | | 3.00E-05 | |  |
| GO:0009152 | purine ribonucleotide biosynthetic process | 1.10E-06 | 9.62E-01 | 1.00E+00 | | 1.00E+00 | | 1.00E+00 | | 1.00E+00 | | 2.85E-05 | |  |
| GO:0009260 | ribonucleotide biosynthetic process | 1.13E-06 | 9.62E-01 | 1.00E+00 | | 1.00E+00 | | 1.00E+00 | | 1.00E+00 | | 3.09E-05 | |  |
| GO:0009150 | purine ribonucleotide metabolic process | 1.21E-06 | 9.39E-01 | 1.00E+00 | | 1.00E+00 | | 1.00E+00 | | 1.00E+00 | | 2.86E-05 | |  |
| GO:1901293 | nucleoside phosphate biosynthetic process | 1.21E-06 | 9.68E-01 | 1.00E+00 | | 1.00E+00 | | 1.00E+00 | | 1.00E+00 | | 3.40E-05 | |  |
| GO:0009165 | nucleotide biosynthetic process | 1.21E-06 | 9.68E-01 | 1.00E+00 | | 1.00E+00 | | 1.00E+00 | | 1.00E+00 | | 3.40E-05 | |  |
| GO:0006511 | ubiquitin-dependent protein catabolic process | 1.22E-06 | 9.33E-01 | 1.00E+00 | | 1.00E+00 | | 1.00E+00 | | 1.00E+00 | | 1.12E-04 | |  |
| GO:0009057 | macromolecule catabolic process | 1.31E-06 | 9.62E-01 | 1.00E+00 | | 1.00E+00 | | 1.00E+00 | | 1.00E+00 | | 3.36E-05 | |  |
| GO:0006164 | purine nucleotide biosynthetic process | 1.32E-06 | 9.62E-01 | 1.00E+00 | | 1.00E+00 | | 1.00E+00 | | 1.00E+00 | | 3.90E-05 | |  |
| GO:0061919 | process utilizing autophagic mechanism | 1.90E-06 | 9.33E-01 | 1.00E+00 | | 1.00E+00 | | 1.00E+00 | | 1.00E+00 | | 1.42E-06 | |  |
| GO:0006914 | autophagy | 1.90E-06 | 9.33E-01 | 1.00E+00 | | 1.00E+00 | | 1.00E+00 | | 1.00E+00 | | 1.42E-06 | |  |
| GO:0072522 | purine-containing compound biosynthetic process | 2.11E-06 | 9.39E-01 | 1.00E+00 | | 1.00E+00 | | 1.00E+00 | | 1.00E+00 | | 3.00E-05 | |  |
| GO:0072521 | purine-containing compound metabolic process | 2.86E-06 | 9.33E-01 | 1.00E+00 | | 1.00E+00 | | 1.00E+00 | | 1.00E+00 | | 3.00E-05 | |  |
| GO:0006457 | protein folding | 2.86E-06 | 9.94E-01 | 1.00E+00 | | 1.00E+00 | | 1.00E+00 | | 1.00E+00 | | 3.53E-05 | |  |
| GO:0034645 | cellular macromolecule biosynthetic process | 5.09E-06 | 9.35E-01 | 1.00E+00 | | 1.00E+00 | | 1.00E+00 | | 1.00E+00 | | 2.74E-05 | |  |
| GO:1903050 | regulation of proteolysis involved in protein catabolic process | 5.91E-06 | 9.80E-01 | 1.00E+00 | | 1.00E+00 | | 1.00E+00 | | 1.00E+00 | | 5.45E-05 | |  |
| GO:1901565 | organonitrogen compound catabolic process | 6.72E-06 | 9.39E-01 | 1.00E+00 | | 1.00E+00 | | 1.00E+00 | | 1.00E+00 | | 3.00E-05 | |  |
| GO:0002181 | cytoplasmic translation | 7.78E-06 | 9.74E-01 | 1.00E+00 | | 1.00E+00 | | 1.00E+00 | | 1.00E+00 | | 2.86E-05 | |  |
| GO:0030036 | actin cytoskeleton organization | 1.28E-05 | 9.68E-01 | 1.00E+00 | | 9.91E-01 | | 1.00E+00 | | 1.00E+00 | | 5.97E-06 | |  |
| GO:0034504 | protein localization to nucleus | 1.62E-05 | 9.99E-01 | 1.00E+00 | | 1.00E+00 | | 1.00E+00 | | 1.00E+00 | | 3.44E-04 | |  |
| GO:0030029 | actin filament-based process | 1.88E-05 | 9.62E-01 | 1.00E+00 | | 9.72E-01 | | 1.00E+00 | | 1.00E+00 | | 2.86E-05 | |  |
| GO:0009123 | nucleoside monophosphate metabolic process | 1.95E-05 | 9.86E-01 | 1.00E+00 | | 1.00E+00 | | 1.00E+00 | | 1.00E+00 | | 1.16E-04 | |  |
| GO:0006413 | translational initiation | 2.92E-05 | 9.33E-01 | 1.00E+00 | | 1.00E+00 | | 1.00E+00 | | 1.00E+00 | | 3.09E-05 | |  |
| GO:0030163 | protein catabolic process | 3.55E-05 | 9.80E-01 | 1.00E+00 | | 1.00E+00 | | 1.00E+00 | | 1.00E+00 | | 1.16E-03 | |  |
| GO:1900180 | regulation of protein localization to nucleus | 3.79E-05 | 9.90E-01 | 1.00E+00 | | 1.00E+00 | | 1.00E+00 | | 1.00E+00 | | 1.08E-03 | |  |
| GO:0007051 | spindle organization | 4.52E-05 | 9.89E-01 | 1.00E+00 | | 1.00E+00 | | 1.00E+00 | | 1.00E+00 | | 1.04E-03 | |  |
| GO:0072594 | establishment of protein localization to organelle | 5.10E-05 | 9.62E-01 | 1.00E+00 | | 1.00E+00 | | 1.00E+00 | | 1.00E+00 | | 1.12E-04 | |  |
| GO:0007264 | small GTPase mediated signal transduction | 5.46E-05 | 9.33E-01 | 1.00E+00 | | 1.00E+00 | | 1.00E+00 | | 1.00E+00 | | 4.84E-05 | |  |
| GO:0031047 | gene silencing by RNA | 5.69E-05 | 9.84E-01 | 1.00E+00 | | 1.00E+00 | | 1.00E+00 | | 1.00E+00 | | 1.79E-05 | |  |
| GO:0070925 | organelle assembly | 5.72E-05 | 9.39E-01 | 1.00E+00 | | 1.00E+00 | | 1.00E+00 | | 1.00E+00 | | 2.85E-05 | |  |
| GO:0070647 | protein modification by small protein conjugation or removal | 5.86E-05 | 9.29E-01 | 1.00E+00 | | 1.00E+00 | | 1.00E+00 | | 1.00E+00 | | 1.28E-03 | |  |
| GO:0061136 | regulation of proteasomal protein catabolic process | 5.86E-05 | 9.84E-01 | 1.00E+00 | | 1.00E+00 | | 1.00E+00 | | 1.00E+00 | | 8.02E-05 | |  |
| GO:0019439 | aromatic compound catabolic process | 8.92E-05 | 9.29E-01 | 1.00E+00 | | 1.00E+00 | | 1.00E+00 | | 1.00E+00 | | 1.16E-04 | |  |
| GO:0022402 | cell cycle process | 9.69E-05 | 9.28E-01 | 1.00E+00 | | 1.00E+00 | | 1.00E+00 | | 1.00E+00 | | 1.87E-04 | |  |
| GO:0007015 | actin filament organization | 1.15E-04 | 9.62E-01 | 1.00E+00 | | 9.91E-01 | | 1.00E+00 | | 1.00E+00 | | 3.40E-05 | |  |
| GO:0046700 | heterocycle catabolic process | 1.15E-04 | 9.33E-01 | 1.00E+00 | | 1.00E+00 | | 1.00E+00 | | 1.00E+00 | | 1.57E-04 | |  |
| GO:2000058 | regulation of ubiquitin-dependent protein catabolic process | 1.30E-04 | 9.84E-01 | 1.00E+00 | | 1.00E+00 | | 1.00E+00 | | 1.00E+00 | | 1.08E-03 | |  |
| GO:0034655 | nucleobase-containing compound catabolic process | 1.34E-04 | 9.33E-01 | 1.00E+00 | | 1.00E+00 | | 1.00E+00 | | 1.00E+00 | | 7.60E-04 | |  |
| GO:0042176 | regulation of protein catabolic process | 1.46E-04 | 9.98E-01 | 1.00E+00 | | 1.00E+00 | | 1.00E+00 | | 1.00E+00 | | 1.32E-03 | |  |
| GO:1903047 | mitotic cell cycle process | 1.49E-04 | 9.29E-01 | 1.00E+00 | | 1.00E+00 | | 1.00E+00 | | 1.00E+00 | | 1.43E-04 | |  |
| GO:0032880 | regulation of protein localization | 1.51E-04 | 9.62E-01 | 1.00E+00 | | 9.27E-01 | | 1.00E+00 | | 1.00E+00 | | 7.02E-05 | |  |
| GO:0007265 | Ras protein signal transduction | 1.75E-04 | 9.39E-01 | 1.00E+00 | | 1.00E+00 | | 1.00E+00 | | 1.00E+00 | | 4.84E-04 | |  |
| GO:1901361 | organic cyclic compound catabolic process | 2.31E-04 | 9.29E-01 | 1.00E+00 | | 1.00E+00 | | 1.00E+00 | | 1.00E+00 | | 2.98E-04 | |  |
| GO:2000113 | negative regulation of cellular macromolecule biosynthetic process | 2.51E-04 | 9.66E-01 | 1.00E+00 | | 1.00E+00 | | 1.00E+00 | | 1.00E+00 | | 1.98E-03 | |  |
| GO:0044270 | cellular nitrogen compound catabolic process | 3.15E-04 | 9.29E-01 | 1.00E+00 | | 1.00E+00 | | 1.00E+00 | | 1.00E+00 | | 2.27E-04 | |  |
| GO:0016071 | mRNA metabolic process | 3.15E-04 | 9.29E-01 | 1.00E+00 | | 1.00E+00 | | 1.00E+00 | | 1.00E+00 | | 9.70E-04 | |  |
| GO:0019692 | deoxyribose phosphate metabolic process | 3.18E-04 | 9.84E-01 | 1.00E+00 | | 1.00E+00 | | 1.00E+00 | | 1.00E+00 | | 6.49E-04 | |  |
| GO:0009394 | 2'-deoxyribonucleotide metabolic process | 3.18E-04 | 9.84E-01 | 1.00E+00 | | 1.00E+00 | | 1.00E+00 | | 1.00E+00 | | 6.49E-04 | |  |
| GO:0031396 | regulation of protein ubiquitination | 3.23E-04 | 9.33E-01 | 1.00E+00 | | 1.00E+00 | | 1.00E+00 | | 1.00E+00 | | 1.72E-03 | |  |
| GO:0051668 | localization within membrane | 3.23E-04 | 9.35E-01 | 1.00E+00 | | 1.00E+00 | | 1.00E+00 | | 1.00E+00 | | 1.42E-03 | |  |
| GO:0060341 | regulation of cellular localization | 3.28E-04 | 9.74E-01 | 1.00E+00 | | 8.88E-01 | | 1.00E+00 | | 1.00E+00 | | 1.87E-04 | |  |
| GO:0033043 | regulation of organelle organization | 3.28E-04 | 9.29E-01 | 1.00E+00 | | 1.00E+00 | | 1.00E+00 | | 1.00E+00 | | 1.16E-04 | |  |
| GO:0043487 | regulation of RNA stability | 3.32E-04 | 9.62E-01 | 1.00E+00 | | 1.00E+00 | | 1.00E+00 | | 1.00E+00 | | 1.79E-03 | |  |
| GO:1903320 | regulation of protein modification by small protein conjugation or removal | 3.33E-04 | 9.29E-01 | 1.00E+00 | | 1.00E+00 | | 1.00E+00 | | 1.00E+00 | | 2.30E-04 | |  |
| GO:0009126 | purine nucleoside monophosphate metabolic process | 3.41E-04 | 9.90E-01 | 1.00E+00 | | 1.00E+00 | | 1.00E+00 | | 1.00E+00 | | 6.49E-04 | |  |
| GO:0009265 | 2'-deoxyribonucleotide biosynthetic process | 3.43E-04 | 1.00E+00 | 1.00E+00 | | 1.00E+00 | | 1.00E+00 | | 1.00E+00 | | 1.79E-03 | |  |
| GO:0009263 | deoxyribonucleotide biosynthetic process | 3.43E-04 | 1.00E+00 | 1.00E+00 | | 1.00E+00 | | 1.00E+00 | | 1.00E+00 | | 1.79E-03 | |  |
| GO:0046385 | deoxyribose phosphate biosynthetic process | 3.43E-04 | 1.00E+00 | 1.00E+00 | | 1.00E+00 | | 1.00E+00 | | 1.00E+00 | | 1.79E-03 | |  |
| GO:0010256 | endomembrane system organization | 3.49E-04 | 9.86E-01 | 1.00E+00 | | 1.00E+00 | | 1.00E+00 | | 1.00E+00 | | 6.49E-04 | |  |
| GO:0000278 | mitotic cell cycle | 3.53E-04 | 9.29E-01 | 1.00E+00 | | 1.00E+00 | | 1.00E+00 | | 1.00E+00 | | 1.70E-04 | |  |
| GO:0010498 | proteasomal protein catabolic process | 3.53E-04 | 9.57E-01 | 1.00E+00 | | 1.00E+00 | | 1.00E+00 | | 1.00E+00 | | 1.75E-03 | |  |
| GO:1900182 | positive regulation of protein localization to nucleus | 3.74E-04 | 9.91E-01 | 1.00E+00 | | 1.00E+00 | | 1.00E+00 | | 1.00E+00 | | 4.66E-03 | |  |
| GO:0043488 | regulation of mRNA stability | 3.93E-04 | 9.39E-01 | 1.00E+00 | | 1.00E+00 | | 1.00E+00 | | 1.00E+00 | | 1.98E-03 | |  |
| GO:0007163 | establishment or maintenance of cell polarity | 4.60E-04 | 9.99E-01 | 1.00E+00 | | 1.00E+00 | | 1.00E+00 | | 1.00E+00 | | 1.91E-03 | |  |
| GO:0061013 | regulation of mRNA catabolic process | 5.09E-04 | 9.35E-01 | 1.00E+00 | | 1.00E+00 | | 1.00E+00 | | 1.00E+00 | | 2.66E-03 | |  |
| GO:0006396 | RNA processing | 5.27E-04 | 9.29E-01 | 1.00E+00 | | 1.00E+00 | | 1.00E+00 | | 1.00E+00 | | 1.50E-02 | |  |
| GO:0009262 | deoxyribonucleotide metabolic process | 5.87E-04 | 9.68E-01 | 1.00E+00 | | 1.00E+00 | | 1.00E+00 | | 1.00E+00 | | 1.33E-03 | |  |
| GO:0009896 | positive regulation of catabolic process | 6.47E-04 | 9.29E-01 | 1.00E+00 | | 1.00E+00 | | 1.00E+00 | | 1.00E+00 | | 4.54E-04 | |  |
| GO:0006402 | mRNA catabolic process | 6.69E-04 | 9.33E-01 | 1.00E+00 | | 1.00E+00 | | 1.00E+00 | | 1.00E+00 | | 4.80E-03 | |  |
| GO:0030168 | platelet activation | 7.47E-04 | 9.86E-01 | 1.00E+00 | | 9.84E-01 | | 1.00E+00 | | 1.00E+00 | | 1.76E-02 | |  |
| GO:1990778 | protein localization to cell periphery | 7.54E-04 | 9.90E-01 | 1.00E+00 | | 1.00E+00 | | 1.00E+00 | | 1.00E+00 | | 2.50E-03 | |  |
| GO:0009167 | purine ribonucleoside monophosphate metabolic process | 8.11E-04 | 9.90E-01 | 1.00E+00 | | 1.00E+00 | | 1.00E+00 | | 1.00E+00 | | 1.34E-03 | |  |
| GO:0070585 | protein localization to mitochondrion | 8.39E-04 | 9.29E-01 | 1.00E+00 | | 1.00E+00 | | 1.00E+00 | | 1.00E+00 | | 3.99E-04 | |  |
| GO:0009161 | ribonucleoside monophosphate metabolic process | 9.24E-04 | 9.80E-01 | 1.00E+00 | | 1.00E+00 | | 1.00E+00 | | 1.00E+00 | | 2.26E-03 | |  |
| GO:0000226 | microtubule cytoskeleton organization | 9.62E-04 | 9.29E-01 | 1.00E+00 | | 1.00E+00 | | 1.00E+00 | | 1.00E+00 | | 7.91E-04 | |  |
| GO:1902850 | microtubule cytoskeleton organization involved in mitosis | 9.62E-04 | 9.80E-01 | 1.00E+00 | | 1.00E+00 | | 1.00E+00 | | 1.00E+00 | | 1.21E-02 | |  |
| GO:0009142 | nucleoside triphosphate biosynthetic process | 1.08E-03 | 9.62E-01 | 1.00E+00 | | 1.00E+00 | | 1.00E+00 | | 1.00E+00 | | 1.07E-02 | |  |
| GO:0032984 | protein-containing complex disassembly | 1.19E-03 | 9.74E-01 | 1.00E+00 | | 1.00E+00 | | 1.00E+00 | | 1.00E+00 | | 1.28E-03 | |  |
| GO:0006913 | nucleocytoplasmic transport | 1.24E-03 | 9.68E-01 | 1.00E+00 | | 1.00E+00 | | 1.00E+00 | | 1.00E+00 | | 1.21E-02 | |  |
| GO:0043436 | oxoacid metabolic process | 1.25E-03 | 9.29E-01 | 1.00E+00 | | 1.00E+00 | | 1.00E+00 | | 1.00E+00 | | 2.98E-04 | |  |
| GO:0017148 | negative regulation of translation | 1.25E-03 | 9.39E-01 | 1.00E+00 | | 1.00E+00 | | 1.00E+00 | | 1.00E+00 | | 5.99E-03 | |  |
| GO:0032446 | protein modification by small protein conjugation | 1.31E-03 | 9.21E-01 | 1.00E+00 | | 1.00E+00 | | 1.00E+00 | | 1.00E+00 | | 2.33E-02 | |  |
| GO:0044282 | small molecule catabolic process | 1.31E-03 | 9.33E-01 | 1.00E+00 | | 1.00E+00 | | 1.00E+00 | | 1.00E+00 | | 1.29E-03 | |  |
| GO:0016441 | post-transcriptional gene silencing | 1.35E-03 | 9.84E-01 | 1.00E+00 | | 1.00E+00 | | 1.00E+00 | | 1.00E+00 | | 2.54E-03 | |  |
| GO:0065003 | protein-containing complex assembly | 1.55E-03 | 1.45E-01 | 1.00E+00 | | 1.00E+00 | | 1.00E+00 | | 1.00E+00 | | 9.35E-06 | |  |
| GO:0006082 | organic acid metabolic process | 1.55E-03 | 9.29E-01 | 1.00E+00 | | 1.00E+00 | | 1.00E+00 | | 1.00E+00 | | 4.20E-04 | |  |
| GO:0009141 | nucleoside triphosphate metabolic process | 1.55E-03 | 9.33E-01 | 1.00E+00 | | 1.00E+00 | | 1.00E+00 | | 1.00E+00 | | 8.68E-03 | |  |
| GO:0051169 | nuclear transport | 1.55E-03 | 9.74E-01 | 1.00E+00 | | 1.00E+00 | | 1.00E+00 | | 1.00E+00 | | 1.48E-02 | |  |
| GO:0035195 | miRNA-mediated gene silencing | 1.60E-03 | 9.90E-01 | 1.00E+00 | | 1.00E+00 | | 1.00E+00 | | 1.00E+00 | | 2.65E-03 | |  |
| GO:0007017 | microtubule-based process | 1.70E-03 | 9.29E-01 | 1.00E+00 | | 1.00E+00 | | 1.00E+00 | | 1.00E+00 | | 2.16E-03 | |  |
| GO:0009112 | nucleobase metabolic process | 1.92E-03 | 9.86E-01 | 1.00E+00 | | 1.00E+00 | | 1.00E+00 | | 1.00E+00 | | 2.66E-03 | |  |
| GO:0006635 | fatty acid beta-oxidation | 2.02E-03 | 9.94E-01 | 1.00E+00 | | 9.87E-01 | | 1.00E+00 | | 1.00E+00 | | 4.34E-03 | |  |
| GO:0019752 | carboxylic acid metabolic process | 2.12E-03 | 9.29E-01 | 1.00E+00 | | 1.00E+00 | | 1.00E+00 | | 1.00E+00 | | 3.73E-04 | |  |
| GO:0032388 | positive regulation of intracellular transport | 2.15E-03 | 9.29E-01 | 1.00E+00 | | 1.00E+00 | | 1.00E+00 | | 1.00E+00 | | 2.30E-03 | |  |
| GO:0090316 | positive regulation of intracellular protein transport | 2.15E-03 | 9.33E-01 | 1.00E+00 | | 1.00E+00 | | 1.00E+00 | | 1.00E+00 | | 6.84E-03 | |  |
| GO:0072527 | pyrimidine-containing compound metabolic process | 2.15E-03 | 9.86E-01 | 1.00E+00 | | 1.00E+00 | | 1.00E+00 | | 1.00E+00 | | 5.80E-03 | |  |
| GO:0051170 | import into nucleus | 2.15E-03 | 9.91E-01 | 1.00E+00 | | 1.00E+00 | | 1.00E+00 | | 1.00E+00 | | 1.42E-02 | |  |
| GO:0036258 | multivesicular body assembly | 2.19E-03 | 9.90E-01 | 1.00E+00 | | 1.00E+00 | | 1.00E+00 | | 1.00E+00 | | 7.53E-03 | |  |
| GO:0061572 | actin filament bundle organization | 2.31E-03 | 9.33E-01 | 1.00E+00 | | 1.00E+00 | | 1.00E+00 | | 1.00E+00 | | 3.65E-03 | |  |
| GO:0009124 | nucleoside monophosphate biosynthetic process | 2.44E-03 | 9.84E-01 | 1.00E+00 | | 1.00E+00 | | 1.00E+00 | | 1.00E+00 | | 4.73E-03 | |  |
| GO:0009162 | deoxyribonucleoside monophosphate metabolic process | 2.46E-03 | 9.80E-01 | 1.00E+00 | | 1.00E+00 | | 1.00E+00 | | 1.00E+00 | | 1.13E-02 | |  |
| GO:0045727 | positive regulation of translation | 2.55E-03 | 9.29E-01 | 1.00E+00 | | 1.00E+00 | | 1.00E+00 | | 1.00E+00 | | 3.57E-03 | |  |
| GO:0032434 | regulation of proteasomal ubiquitin-dependent protein catabolic process | 2.55E-03 | 9.90E-01 | 1.00E+00 | | 1.00E+00 | | 1.00E+00 | | 1.00E+00 | | 3.57E-03 | |  |
| GO:0097190 | apoptotic signaling pathway | 2.72E-03 | 9.74E-01 | 1.00E+00 | | 1.00E+00 | | 1.00E+00 | | 1.00E+00 | | 1.76E-03 | |  |
| GO:0009127 | purine nucleoside monophosphate biosynthetic process | 2.74E-03 | 9.94E-01 | 1.00E+00 | | 1.00E+00 | | 1.00E+00 | | 1.00E+00 | | 1.79E-03 | |  |
| GO:0046037 | GMP metabolic process | 2.74E-03 | 9.94E-01 | 1.00E+00 | | 1.00E+00 | | 1.00E+00 | | 1.00E+00 | | 1.07E-02 | |  |
| GO:0051017 | actin filament bundle assembly | 2.94E-03 | 9.33E-01 | 1.00E+00 | | 1.00E+00 | | 1.00E+00 | | 1.00E+00 | | 4.32E-03 | |  |
| GO:0035194 | post-transcriptional gene silencing by RNA | 2.94E-03 | 9.80E-01 | 1.00E+00 | | 1.00E+00 | | 1.00E+00 | | 1.00E+00 | | 5.04E-03 | |  |
| GO:0010506 | regulation of autophagy | 3.07E-03 | 9.33E-01 | 1.00E+00 | | 1.00E+00 | | 1.00E+00 | | 1.00E+00 | | 6.74E-04 | |  |
| GO:0072659 | protein localization to plasma membrane | 3.09E-03 | 9.85E-01 | 1.00E+00 | | 9.72E-01 | | 1.00E+00 | | 1.00E+00 | | 8.68E-03 | |  |
| GO:0072655 | establishment of protein localization to mitochondrion | 3.24E-03 | 9.29E-01 | 1.00E+00 | | 1.00E+00 | | 1.00E+00 | | 1.00E+00 | | 1.42E-03 | |  |
| GO:0007052 | mitotic spindle organization | 3.40E-03 | 9.90E-01 | 1.00E+00 | | 1.00E+00 | | 1.00E+00 | | 1.00E+00 | | 5.06E-02 | |  |
| GO:0034249 | negative regulation of cellular amide metabolic process | 3.53E-03 | 9.33E-01 | 1.00E+00 | | 1.00E+00 | | 1.00E+00 | | 1.00E+00 | | 1.93E-02 | |  |
| GO:0097193 | intrinsic apoptotic signaling pathway | 3.57E-03 | 9.39E-01 | 1.00E+00 | | 1.00E+00 | | 1.00E+00 | | 1.00E+00 | | 1.43E-02 | |  |
| GO:0051726 | regulation of cell cycle | 3.60E-03 | 9.29E-01 | 1.00E+00 | | 1.00E+00 | | 1.00E+00 | | 1.00E+00 | | 1.17E-02 | |  |
| GO:1903311 | regulation of mRNA metabolic process | 3.61E-03 | 9.35E-01 | 1.00E+00 | | 1.00E+00 | | 1.00E+00 | | 1.00E+00 | | 8.68E-03 | |  |
| GO:0032386 | regulation of intracellular transport | 3.65E-03 | 9.33E-01 | 1.00E+00 | | 1.00E+00 | | 1.00E+00 | | 1.00E+00 | | 4.34E-03 | |  |
| GO:0016241 | regulation of macroautophagy | 3.73E-03 | 9.29E-01 | 1.00E+00 | | 1.00E+00 | | 1.00E+00 | | 1.00E+00 | | 1.99E-04 | |  |
| GO:0006839 | mitochondrial transport | 3.73E-03 | 9.39E-01 | 1.00E+00 | | 1.00E+00 | | 1.00E+00 | | 1.00E+00 | | 7.60E-03 | |  |
| GO:0072657 | protein localization to membrane | 4.13E-03 | 9.39E-01 | 1.00E+00 | | 1.00E+00 | | 1.00E+00 | | 1.00E+00 | | 1.27E-02 | |  |
| GO:0035196 | miRNA processing | 4.21E-03 | 9.93E-01 | 1.00E+00 | | 9.93E-01 | | 1.00E+00 | | 1.00E+00 | | 5.48E-03 | |  |
| GO:0006220 | pyrimidine nucleotide metabolic process | 4.21E-03 | 9.84E-01 | 1.00E+00 | | 1.00E+00 | | 1.00E+00 | | 1.00E+00 | | 5.48E-03 | |  |
| GO:0030031 | cell projection assembly | 4.30E-03 | 9.84E-01 | 1.00E+00 | | 9.23E-01 | | 1.00E+00 | | 1.00E+00 | | 1.04E-03 | |  |
| GO:0006091 | generation of precursor metabolites and energy | 4.30E-03 | 9.29E-01 | 1.00E+00 | | 1.00E+00 | | 1.00E+00 | | 1.00E+00 | | 1.87E-02 | |  |
| GO:0043161 | proteasome-mediated ubiquitin-dependent protein catabolic process | 4.65E-03 | 9.62E-01 | 1.00E+00 | | 1.00E+00 | | 1.00E+00 | | 1.00E+00 | | 1.43E-02 | |  |
| GO:0033157 | regulation of intracellular protein transport | 4.88E-03 | 9.29E-01 | 1.00E+00 | | 1.00E+00 | | 1.00E+00 | | 1.00E+00 | | 1.42E-02 | |  |
| GO:0016567 | protein ubiquitination | 4.88E-03 | 9.29E-01 | 1.00E+00 | | 1.00E+00 | | 1.00E+00 | | 1.00E+00 | | 9.22E-02 | |  |
| GO:0051348 | negative regulation of transferase activity | 4.89E-03 | 9.80E-01 | 1.00E+00 | | 1.00E+00 | | 1.00E+00 | | 1.00E+00 | | 3.08E-02 | |  |
| GO:0007032 | endosome organization | 5.09E-03 | 9.98E-01 | 1.00E+00 | | 1.00E+00 | | 1.00E+00 | | 1.00E+00 | | 2.57E-02 | |  |
| GO:0022411 | cellular component disassembly | 5.21E-03 | 9.62E-01 | 1.00E+00 | | 7.70E-01 | | 1.00E+00 | | 1.00E+00 | | 5.48E-03 | |  |
| GO:0016197 | endosomal transport | 5.21E-03 | 9.29E-01 | 1.00E+00 | | 1.00E+00 | | 1.00E+00 | | 1.00E+00 | | 8.12E-03 | |  |
| GO:0006183 | GTP biosynthetic process | 5.21E-03 | 1.00E+00 | 1.00E+00 | | 1.00E+00 | | 1.00E+00 | | 1.00E+00 | | 1.49E-02 | |  |
| GO:0032263 | GMP salvage | 5.21E-03 | 1.00E+00 | 1.00E+00 | | 1.00E+00 | | 1.00E+00 | | 1.00E+00 | | 1.49E-02 | |  |
| GO:0006626 | protein targeting to mitochondrion | 5.22E-03 | 9.29E-01 | 1.00E+00 | | 1.00E+00 | | 1.00E+00 | | 1.00E+00 | | 1.17E-02 | |  |
| GO:0009062 | fatty acid catabolic process | 5.22E-03 | 9.84E-01 | 1.00E+00 | | 1.00E+00 | | 1.00E+00 | | 1.00E+00 | | 1.17E-02 | |  |
| GO:0140694 | non-membrane-bounded organelle assembly | 5.34E-03 | 9.29E-01 | 1.00E+00 | | 1.00E+00 | | 1.00E+00 | | 1.00E+00 | | 1.93E-02 | |  |
| GO:0018107 | peptidyl-threonine phosphorylation | 5.34E-03 | 9.33E-01 | 1.00E+00 | | 1.00E+00 | | 1.00E+00 | | 1.00E+00 | | 1.90E-02 | |  |
| GO:0072528 | pyrimidine-containing compound biosynthetic process | 5.58E-03 | 9.74E-01 | 1.00E+00 | | 1.00E+00 | | 1.00E+00 | | 1.00E+00 | | 7.06E-02 | |  |
| GO:0009221 | pyrimidine deoxyribonucleotide biosynthetic process | 5.98E-03 | 1.00E+00 | 1.00E+00 | | 1.00E+00 | | 1.00E+00 | | 1.00E+00 | | 1.69E-02 | |  |
| GO:0009144 | purine nucleoside triphosphate metabolic process | 6.01E-03 | 9.29E-01 | 1.00E+00 | | 1.00E+00 | | 1.00E+00 | | 1.00E+00 | | 2.17E-02 | |  |
| GO:0044093 | positive regulation of molecular function | 6.12E-03 | 9.74E-01 | 1.00E+00 | | 7.43E-01 | | 1.00E+00 | | 1.00E+00 | | 2.65E-03 | |  |
| GO:0009219 | pyrimidine deoxyribonucleotide metabolic process | 6.24E-03 | 9.84E-01 | 1.00E+00 | | 1.00E+00 | | 1.00E+00 | | 1.00E+00 | | 4.98E-03 | |  |
| GO:0006221 | pyrimidine nucleotide biosynthetic process | 6.24E-03 | 9.84E-01 | 1.00E+00 | | 1.00E+00 | | 1.00E+00 | | 1.00E+00 | | 2.25E-02 | |  |
| GO:0009145 | purine nucleoside triphosphate biosynthetic process | 6.44E-03 | 9.33E-01 | 1.00E+00 | | 1.00E+00 | | 1.00E+00 | | 1.00E+00 | | 3.82E-02 | |  |
| GO:0006144 | purine nucleobase metabolic process | 6.62E-03 | 9.80E-01 | 1.00E+00 | | 1.00E+00 | | 1.00E+00 | | 1.00E+00 | | 2.21E-02 | |  |
| GO:0036257 | multivesicular body organization | 6.62E-03 | 9.90E-01 | 1.00E+00 | | 1.00E+00 | | 1.00E+00 | | 1.00E+00 | | 2.06E-02 | |  |
| GO:0009168 | purine ribonucleoside monophosphate biosynthetic process | 6.62E-03 | 9.93E-01 | 1.00E+00 | | 1.00E+00 | | 1.00E+00 | | 1.00E+00 | | 4.25E-03 | |  |
| GO:0009129 | pyrimidine nucleoside monophosphate metabolic process | 6.62E-03 | 9.93E-01 | 1.00E+00 | | 1.00E+00 | | 1.00E+00 | | 1.00E+00 | | 2.21E-02 | |  |
| GO:0006401 | RNA catabolic process | 6.72E-03 | 9.39E-01 | 1.00E+00 | | 1.00E+00 | | 1.00E+00 | | 1.00E+00 | | 2.08E-02 | |  |
| GO:1904375 | regulation of protein localization to cell periphery | 6.94E-03 | 9.74E-01 | 1.00E+00 | | 1.00E+00 | | 1.00E+00 | | 1.00E+00 | | 1.19E-02 | |  |
| GO:0097435 | supramolecular fiber organization | 6.98E-03 | 9.39E-01 | 1.00E+00 | | 6.13E-01 | | 1.00E+00 | | 1.00E+00 | | 2.32E-03 | |  |
| GO:0034250 | positive regulation of cellular amide metabolic process | 6.98E-03 | 9.29E-01 | 1.00E+00 | | 1.00E+00 | | 1.00E+00 | | 1.00E+00 | | 1.43E-02 | |  |
| GO:0016050 | vesicle organization | 6.98E-03 | 9.39E-01 | 1.00E+00 | | 1.00E+00 | | 1.00E+00 | | 1.00E+00 | | 8.40E-04 | |  |
| GO:0070918 | small regulatory ncRNA processing | 7.33E-03 | 9.86E-01 | 1.00E+00 | | 9.99E-01 | | 1.00E+00 | | 1.00E+00 | | 1.07E-02 | |  |
| GO:0009205 | purine ribonucleoside triphosphate metabolic process | 7.33E-03 | 9.29E-01 | 1.00E+00 | | 1.00E+00 | | 1.00E+00 | | 1.00E+00 | | 2.52E-02 | |  |
| GO:0060491 | regulation of cell projection assembly | 7.35E-03 | 9.80E-01 | 1.00E+00 | | 7.43E-01 | | 1.00E+00 | | 1.00E+00 | | 3.43E-02 | |  |
| GO:0006470 | protein dephosphorylation | 7.35E-03 | 9.74E-01 | 1.00E+00 | | 1.00E+00 | | 1.00E+00 | | 1.00E+00 | | 1.07E-02 | |  |
| GO:0006606 | protein import into nucleus | 7.82E-03 | 9.90E-01 | 1.00E+00 | | 1.00E+00 | | 1.00E+00 | | 1.00E+00 | | 3.75E-02 | |  |
| GO:0031331 | positive regulation of cellular catabolic process | 7.82E-03 | 9.29E-01 | 1.00E+00 | | 1.00E+00 | | 1.00E+00 | | 1.00E+00 | | 2.91E-03 | |  |
| GO:0006979 | response to oxidative stress | 7.87E-03 | 9.29E-01 | 1.00E+00 | | 8.00E-01 | | 1.00E+00 | | 1.00E+00 | | 1.58E-03 | |  |
| GO:0031400 | negative regulation of protein modification process | 7.90E-03 | 9.29E-01 | 1.00E+00 | | 9.29E-01 | | 1.00E+00 | | 1.00E+00 | | 1.41E-02 | |  |
| GO:0051493 | regulation of cytoskeleton organization | 7.90E-03 | 9.33E-01 | 1.00E+00 | | 9.84E-01 | | 1.00E+00 | | 1.00E+00 | | 2.06E-03 | |  |
| GO:0030865 | cortical cytoskeleton organization | 7.90E-03 | 9.92E-01 | 1.00E+00 | | 1.00E+00 | | 1.00E+00 | | 1.00E+00 | | 4.73E-03 | |  |
| GO:0062197 | cellular response to chemical stress | 8.24E-03 | 9.39E-01 | 1.00E+00 | | 6.68E-01 | | 1.00E+00 | | 1.00E+00 | | 3.36E-03 | |  |
| GO:0051276 | chromosome organization | 8.24E-03 | 9.29E-01 | 1.00E+00 | | 1.00E+00 | | 1.00E+00 | | 1.00E+00 | | 1.39E-02 | |  |
| GO:0072329 | monocarboxylic acid catabolic process | 8.40E-03 | 9.62E-01 | 1.00E+00 | | 1.00E+00 | | 1.00E+00 | | 1.00E+00 | | 2.52E-02 | |  |
| GO:0008277 | regulation of G protein-coupled receptor signaling pathway | 8.62E-03 | 9.86E-01 | 1.00E+00 | | 9.77E-01 | | 1.00E+00 | | 1.00E+00 | | 3.50E-02 | |  |
| GO:1903322 | positive regulation of protein modification by small protein conjugation or removal | 8.62E-03 | 9.33E-01 | 1.00E+00 | | 1.00E+00 | | 1.00E+00 | | 1.00E+00 | | 7.60E-03 | |  |
| GO:0009060 | aerobic respiration | 8.62E-03 | 9.39E-01 | 1.00E+00 | | 1.00E+00 | | 1.00E+00 | | 1.00E+00 | | 1.84E-01 | |  |
| GO:0045454 | cell redox homeostasis | 8.62E-03 | 9.92E-01 | 1.00E+00 | | 1.00E+00 | | 1.00E+00 | | 1.00E+00 | | 1.09E-02 | |  |
| GO:1902115 | regulation of organelle assembly | 8.64E-03 | 9.93E-01 | 1.00E+00 | | 8.52E-01 | | 1.00E+00 | | 1.00E+00 | | 1.39E-01 | |  |
| GO:0009199 | ribonucleoside triphosphate metabolic process | 8.79E-03 | 9.29E-01 | 1.00E+00 | | 1.00E+00 | | 1.00E+00 | | 1.00E+00 | | 3.08E-02 | |  |
| GO:0046939 | nucleotide phosphorylation | 9.16E-03 | 9.29E-01 | 1.00E+00 | | 1.00E+00 | | 1.00E+00 | | 1.00E+00 | | 1.95E-02 | |  |
| GO:0071826 | ribonucleoprotein complex subunit organization | 9.16E-03 | 9.29E-01 | 1.00E+00 | | 1.00E+00 | | 1.00E+00 | | 1.00E+00 | | 5.06E-02 | |  |
| GO:1903052 | positive regulation of proteolysis involved in protein catabolic process | 9.16E-03 | 9.62E-01 | 1.00E+00 | | 1.00E+00 | | 1.00E+00 | | 1.00E+00 | | 9.39E-03 | |  |
| GO:0051640 | organelle localization | 9.37E-03 | 9.29E-01 | 1.00E+00 | | 1.00E+00 | | 1.00E+00 | | 1.00E+00 | | 6.49E-03 | |  |
| GO:1903076 | regulation of protein localization to plasma membrane | 1.02E-02 | 9.84E-01 | 1.00E+00 | | 9.84E-01 | | 1.00E+00 | | 1.00E+00 | | 4.44E-02 | |  |
| GO:0098876 | vesicle-mediated transport to the plasma membrane | 1.03E-02 | 9.35E-01 | 1.00E+00 | | 1.00E+00 | | 1.00E+00 | | 1.00E+00 | | 3.54E-02 | |  |
| GO:0051656 | establishment of organelle localization | 1.05E-02 | 9.29E-01 | 1.00E+00 | | 1.00E+00 | | 1.00E+00 | | 1.00E+00 | | 9.38E-03 | |  |
| GO:0006605 | protein targeting | 1.15E-02 | 9.29E-01 | 1.00E+00 | | 1.00E+00 | | 1.00E+00 | | 1.00E+00 | | 1.00E-02 | |  |
| GO:0070527 | platelet aggregation | 1.19E-02 | 9.90E-01 | 1.00E+00 | | 9.84E-01 | | 1.00E+00 | | 1.00E+00 | | 1.42E-01 | |  |
| GO:0018105 | peptidyl-serine phosphorylation | 1.19E-02 | 9.29E-01 | 1.00E+00 | | 1.00E+00 | | 1.00E+00 | | 1.00E+00 | | 4.21E-03 | |  |
| GO:0009206 | purine ribonucleoside triphosphate biosynthetic process | 1.19E-02 | 9.33E-01 | 1.00E+00 | | 1.00E+00 | | 1.00E+00 | | 1.00E+00 | | 5.89E-02 | |  |
| GO:0006749 | glutathione metabolic process | 1.19E-02 | 9.74E-01 | 1.00E+00 | | 1.00E+00 | | 1.00E+00 | | 1.00E+00 | | 3.57E-03 | |  |
| GO:0016311 | dephosphorylation | 1.19E-02 | 9.74E-01 | 1.00E+00 | | 1.00E+00 | | 1.00E+00 | | 1.00E+00 | | 2.10E-02 | |  |
| GO:0038095 | Fc-epsilon receptor signaling pathway | 1.24E-02 | 9.97E-01 | 1.00E+00 | | 1.00E+00 | | 1.00E+00 | | 1.00E+00 | | 4.16E-02 | |  |
| GO:0120032 | regulation of plasma membrane bounded cell projection assembly | 1.24E-02 | 9.79E-01 | 1.00E+00 | | 7.38E-01 | | 1.00E+00 | | 1.00E+00 | | 4.73E-02 | |  |
| GO:0042306 | regulation of protein import into nucleus | 1.24E-02 | 9.74E-01 | 1.00E+00 | | 9.84E-01 | | 1.00E+00 | | 1.00E+00 | | 5.50E-02 | |  |
| GO:0000045 | autophagosome assembly | 1.24E-02 | 9.29E-01 | 1.00E+00 | | 1.00E+00 | | 1.00E+00 | | 1.00E+00 | | 2.30E-02 | |  |
| GO:0016482 | cytosolic transport | 1.24E-02 | 9.29E-01 | 1.00E+00 | | 1.00E+00 | | 1.00E+00 | | 1.00E+00 | | 2.57E-02 | |  |
| GO:1901800 | positive regulation of proteasomal protein catabolic process | 1.24E-02 | 9.39E-01 | 1.00E+00 | | 1.00E+00 | | 1.00E+00 | | 1.00E+00 | | 8.28E-03 | |  |
| GO:0061077 | chaperone-mediated protein folding | 1.37E-02 | 9.80E-01 | 1.00E+00 | | 9.14E-01 | | 1.00E+00 | | 1.00E+00 | | 1.39E-02 | |  |
| GO:0120031 | plasma membrane bounded cell projection assembly | 1.37E-02 | 9.80E-01 | 1.00E+00 | | 9.33E-01 | | 1.00E+00 | | 1.00E+00 | | 2.91E-03 | |  |
| GO:0034660 | ncRNA metabolic process | 1.37E-02 | 9.29E-01 | 1.00E+00 | | 1.00E+00 | | 1.00E+00 | | 1.00E+00 | | 1.42E-02 | |  |
| GO:0046039 | GTP metabolic process | 1.41E-02 | 9.39E-01 | 1.00E+00 | | 1.00E+00 | | 1.00E+00 | | 1.00E+00 | | 1.07E-02 | |  |
| GO:0071763 | nuclear membrane organization | 1.41E-02 | 9.39E-01 | 1.00E+00 | | 1.00E+00 | | 1.00E+00 | | 1.00E+00 | | 1.07E-02 | |  |
| GO:1903649 | regulation of cytoplasmic transport | 1.41E-02 | 9.84E-01 | 1.00E+00 | | 1.00E+00 | | 1.00E+00 | | 1.00E+00 | | 4.19E-02 | |  |
| GO:0018193 | peptidyl-amino acid modification | 1.44E-02 | 9.29E-01 | 1.00E+00 | | 9.57E-01 | | 1.00E+00 | | 1.00E+00 | | 7.14E-04 | |  |
| GO:0042307 | positive regulation of protein import into nucleus | 1.49E-02 | 9.84E-01 | 1.00E+00 | | 9.93E-01 | | 1.00E+00 | | 1.00E+00 | | 5.46E-02 | |  |
| GO:0051310 | metaphase plate congression | 1.49E-02 | 9.29E-01 | 1.00E+00 | | 1.00E+00 | | 1.00E+00 | | 1.00E+00 | | 1.35E-01 | |  |
| GO:2000060 | positive regulation of ubiquitin-dependent protein catabolic process | 1.49E-02 | 9.33E-01 | 1.00E+00 | | 1.00E+00 | | 1.00E+00 | | 1.00E+00 | | 1.17E-02 | |  |
| GO:0034030 | ribonucleoside bisphosphate biosynthetic process | 1.49E-02 | 9.33E-01 | 1.00E+00 | | 1.00E+00 | | 1.00E+00 | | 1.00E+00 | | 4.19E-02 | |  |
| GO:0034033 | purine nucleoside bisphosphate biosynthetic process | 1.49E-02 | 9.33E-01 | 1.00E+00 | | 1.00E+00 | | 1.00E+00 | | 1.00E+00 | | 4.19E-02 | |  |
| GO:0033866 | nucleoside bisphosphate biosynthetic process | 1.49E-02 | 9.33E-01 | 1.00E+00 | | 1.00E+00 | | 1.00E+00 | | 1.00E+00 | | 4.19E-02 | |  |
| GO:0099170 | postsynaptic modulation of chemical synaptic transmission | 1.49E-02 | 9.33E-01 | 1.00E+00 | | 1.00E+00 | | 1.00E+00 | | 1.00E+00 | | 1.45E-01 | |  |
| GO:0010833 | telomere maintenance via telomere lengthening | 1.49E-02 | 9.62E-01 | 1.00E+00 | | 1.00E+00 | | 1.00E+00 | | 1.00E+00 | | 1.95E-02 | |  |
| GO:0009166 | nucleotide catabolic process | 1.49E-02 | 9.62E-01 | 1.00E+00 | | 1.00E+00 | | 1.00E+00 | | 1.00E+00 | | 3.09E-02 | |  |
| GO:0034032 | purine nucleoside bisphosphate metabolic process | 1.49E-02 | 9.74E-01 | 1.00E+00 | | 1.00E+00 | | 1.00E+00 | | 1.00E+00 | | 3.94E-02 | |  |
| GO:0033865 | nucleoside bisphosphate metabolic process | 1.49E-02 | 9.74E-01 | 1.00E+00 | | 1.00E+00 | | 1.00E+00 | | 1.00E+00 | | 3.94E-02 | |  |
| GO:0033875 | ribonucleoside bisphosphate metabolic process | 1.49E-02 | 9.74E-01 | 1.00E+00 | | 1.00E+00 | | 1.00E+00 | | 1.00E+00 | | 3.94E-02 | |  |
| GO:0019068 | virion assembly | 1.49E-02 | 9.74E-01 | 1.00E+00 | | 1.00E+00 | | 1.00E+00 | | 1.00E+00 | | 4.19E-02 | |  |
| GO:0002183 | cytoplasmic translational initiation | 1.49E-02 | 9.84E-01 | 1.00E+00 | | 1.00E+00 | | 1.00E+00 | | 1.00E+00 | | 5.99E-03 | |  |
| GO:0032261 | purine nucleotide salvage | 1.49E-02 | 9.91E-01 | 1.00E+00 | | 1.00E+00 | | 1.00E+00 | | 1.00E+00 | | 9.61E-03 | |  |
| GO:0006515 | protein quality control for misfolded or incompletely synthesized proteins | 1.49E-02 | 1.00E+00 | 1.00E+00 | | 1.00E+00 | | 1.00E+00 | | 1.00E+00 | | 3.82E-02 | |  |
| GO:0006177 | GMP biosynthetic process | 1.49E-02 | 1.00E+00 | 1.00E+00 | | 1.00E+00 | | 1.00E+00 | | 1.00E+00 | | 3.82E-02 | |  |
| GO:0032204 | regulation of telomere maintenance | 1.49E-02 | 9.33E-01 | 1.00E+00 | | 1.00E+00 | | 1.00E+00 | | 1.00E+00 | | 2.57E-02 | |  |
| GO:2000641 | regulation of early endosome to late endosome transport | 1.55E-02 | 9.90E-01 | 1.00E+00 | | 9.72E-01 | | 1.00E+00 | | 1.00E+00 | | 4.19E-02 | |  |
| GO:0000413 | protein peptidyl-prolyl isomerization | 1.55E-02 | 1.00E+00 | 1.00E+00 | | 9.72E-01 | | 1.00E+00 | | 1.00E+00 | | 4.19E-02 | |  |
| GO:0046073 | dTMP metabolic process | 1.55E-02 | 9.90E-01 | 1.00E+00 | | 1.00E+00 | | 1.00E+00 | | 1.00E+00 | | 4.19E-02 | |  |
| GO:0009176 | pyrimidine deoxyribonucleoside monophosphate metabolic process | 1.55E-02 | 9.90E-01 | 1.00E+00 | | 1.00E+00 | | 1.00E+00 | | 1.00E+00 | | 4.19E-02 | |  |
| GO:0030705 | cytoskeleton-dependent intracellular transport | 1.58E-02 | 9.33E-01 | 1.00E+00 | | 1.00E+00 | | 1.00E+00 | | 1.00E+00 | | 9.39E-03 | |  |
| GO:0009895 | negative regulation of catabolic process | 1.58E-02 | 9.74E-01 | 1.00E+00 | | 1.00E+00 | | 1.00E+00 | | 1.00E+00 | | 3.06E-02 | |  |
| GO:0035966 | response to topologically incorrect protein | 1.63E-02 | 9.91E-01 | 1.00E+00 | | 9.57E-01 | | 1.00E+00 | | 1.00E+00 | | 1.07E-02 | |  |
| GO:0009201 | ribonucleoside triphosphate biosynthetic process | 1.63E-02 | 9.33E-01 | 1.00E+00 | | 1.00E+00 | | 1.00E+00 | | 1.00E+00 | | 8.16E-02 | |  |
| GO:0033673 | negative regulation of kinase activity | 1.66E-02 | 9.84E-01 | 1.00E+00 | | 9.99E-01 | | 1.00E+00 | | 1.00E+00 | | 4.08E-02 | |  |
| GO:0009132 | nucleoside diphosphate metabolic process | 1.66E-02 | 9.39E-01 | 1.00E+00 | | 1.00E+00 | | 1.00E+00 | | 1.00E+00 | | 4.19E-02 | |  |
| GO:0000910 | cytokinesis | 1.69E-02 | 9.29E-01 | 1.00E+00 | | 1.00E+00 | | 1.00E+00 | | 1.00E+00 | | 4.74E-03 | |  |
| GO:0010563 | negative regulation of phosphorus metabolic process | 1.69E-02 | 9.39E-01 | 1.00E+00 | | 8.88E-01 | | 1.00E+00 | | 1.00E+00 | | 3.43E-02 | |  |
| GO:0007080 | mitotic metaphase plate congression | 1.75E-02 | 9.29E-01 | 1.00E+00 | | 1.00E+00 | | 1.00E+00 | | 1.00E+00 | | 1.55E-01 | |  |
| GO:0009156 | ribonucleoside monophosphate biosynthetic process | 1.75E-02 | 9.80E-01 | 1.00E+00 | | 1.00E+00 | | 1.00E+00 | | 1.00E+00 | | 2.09E-02 | |  |
| GO:0000723 | telomere maintenance | 1.84E-02 | 9.29E-01 | 1.00E+00 | | 1.00E+00 | | 1.00E+00 | | 1.00E+00 | | 4.31E-02 | |  |
| GO:0051303 | establishment of chromosome localization | 1.87E-02 | 9.33E-01 | 1.00E+00 | | 1.00E+00 | | 1.00E+00 | | 1.00E+00 | | 1.56E-01 | |  |
| GO:0050000 | chromosome localization | 1.87E-02 | 9.33E-01 | 1.00E+00 | | 1.00E+00 | | 1.00E+00 | | 1.00E+00 | | 1.56E-01 | |  |
| GO:0031032 | actomyosin structure organization | 1.90E-02 | 9.74E-01 | 1.00E+00 | | 9.14E-01 | | 1.00E+00 | | 1.00E+00 | | 5.66E-02 | |  |
| GO:0034976 | response to endoplasmic reticulum stress | 1.94E-02 | 9.84E-01 | 1.00E+00 | | 1.00E+00 | | 1.00E+00 | | 1.00E+00 | | 2.06E-02 | |  |
| GO:0030010 | establishment of cell polarity | 1.96E-02 | 9.98E-01 | 1.00E+00 | | 9.93E-01 | | 1.00E+00 | | 1.00E+00 | | 7.32E-02 | |  |
| GO:0034470 | ncRNA processing | 1.96E-02 | 9.33E-01 | 1.00E+00 | | 1.00E+00 | | 1.00E+00 | | 1.00E+00 | | 1.24E-01 | |  |
| GO:0019395 | fatty acid oxidation | 2.01E-02 | 9.62E-01 | 1.00E+00 | | 1.00E+00 | | 1.00E+00 | | 1.00E+00 | | 5.14E-02 | |  |
| GO:0051338 | regulation of transferase activity | 2.06E-02 | 9.39E-01 | 1.00E+00 | | 8.52E-01 | | 1.00E+00 | | 1.00E+00 | | 6.40E-02 | |  |
| GO:0046395 | carboxylic acid catabolic process | 2.13E-02 | 9.33E-01 | 1.00E+00 | | 1.00E+00 | | 1.00E+00 | | 1.00E+00 | | 6.49E-03 | |  |
| GO:0006998 | nuclear envelope organization | 2.16E-02 | 9.33E-01 | 1.00E+00 | | 1.00E+00 | | 1.00E+00 | | 1.00E+00 | | 5.44E-03 | |  |
| GO:1901137 | carbohydrate derivative biosynthetic process | 2.19E-02 | 9.84E-01 | 1.00E+00 | | 1.00E+00 | | 1.00E+00 | | 1.00E+00 | | 3.26E-02 | |  |
| GO:1903829 | positive regulation of protein localization | 2.23E-02 | 9.29E-01 | 1.00E+00 | | 9.01E-01 | | 1.00E+00 | | 1.00E+00 | | 8.62E-03 | |  |
| GO:0010638 | positive regulation of organelle organization | 2.29E-02 | 9.29E-01 | 1.00E+00 | | 1.00E+00 | | 1.00E+00 | | 1.00E+00 | | 1.44E-03 | |  |
| GO:0031398 | positive regulation of protein ubiquitination | 2.31E-02 | 9.29E-01 | 1.00E+00 | | 1.00E+00 | | 1.00E+00 | | 1.00E+00 | | 1.60E-02 | |  |
| GO:1905475 | regulation of protein localization to membrane | 2.31E-02 | 9.74E-01 | 1.00E+00 | | 1.00E+00 | | 1.00E+00 | | 1.00E+00 | | 4.19E-02 | |  |
| GO:0045936 | negative regulation of phosphate metabolic process | 2.38E-02 | 9.35E-01 | 1.00E+00 | | 8.88E-01 | | 1.00E+00 | | 1.00E+00 | | 4.19E-02 | |  |
| GO:0045931 | positive regulation of mitotic cell cycle | 2.43E-02 | 9.29E-01 | 1.00E+00 | | 1.00E+00 | | 1.00E+00 | | 1.00E+00 | | 2.13E-01 | |  |
| GO:1902903 | regulation of supramolecular fiber organization | 2.59E-02 | 9.66E-01 | 1.00E+00 | | 8.59E-01 | | 1.00E+00 | | 1.00E+00 | | 4.04E-03 | |  |
| GO:0051056 | regulation of small GTPase mediated signal transduction | 2.60E-02 | 9.74E-01 | 1.00E+00 | | 9.93E-01 | | 1.00E+00 | | 1.00E+00 | | 5.46E-02 | |  |
| GO:0006986 | response to unfolded protein | 2.61E-02 | 9.90E-01 | 1.00E+00 | | 9.57E-01 | | 1.00E+00 | | 1.00E+00 | | 2.57E-02 | |  |
| GO:0097352 | autophagosome maturation | 2.61E-02 | 9.84E-01 | 1.00E+00 | | 1.00E+00 | | 1.00E+00 | | 1.00E+00 | | 7.75E-02 | |  |
| GO:0051298 | centrosome duplication | 2.61E-02 | 9.84E-01 | 1.00E+00 | | 1.00E+00 | | 1.00E+00 | | 1.00E+00 | | 1.88E-01 | |  |
| GO:0007215 | glutamate receptor signaling pathway | 2.61E-02 | 9.94E-01 | 1.00E+00 | | 1.00E+00 | | 1.00E+00 | | 1.00E+00 | | 7.75E-02 | |  |
| GO:0022613 | ribonucleoprotein complex biogenesis | 2.64E-02 | 9.29E-01 | 1.00E+00 | | 1.00E+00 | | 1.00E+00 | | 1.00E+00 | | 2.42E-01 | |  |
| GO:1902532 | negative regulation of intracellular signal transduction | 2.68E-02 | 9.62E-01 | 1.00E+00 | | 9.71E-01 | | 1.00E+00 | | 1.00E+00 | | 1.35E-01 | |  |
| GO:0022618 | ribonucleoprotein complex assembly | 2.68E-02 | 9.29E-01 | 1.00E+00 | | 1.00E+00 | | 1.00E+00 | | 1.00E+00 | | 1.06E-01 | |  |
| GO:1905037 | autophagosome organization | 2.68E-02 | 9.33E-01 | 1.00E+00 | | 1.00E+00 | | 1.00E+00 | | 1.00E+00 | | 4.75E-02 | |  |
| GO:0045732 | positive regulation of protein catabolic process | 2.68E-02 | 9.84E-01 | 1.00E+00 | | 1.00E+00 | | 1.00E+00 | | 1.00E+00 | | 4.17E-02 | |  |
| GO:0044772 | mitotic cell cycle phase transition | 2.72E-02 | 9.28E-01 | 1.00E+00 | | 1.00E+00 | | 1.00E+00 | | 1.00E+00 | | 7.22E-02 | |  |
| GO:0034440 | lipid oxidation | 2.78E-02 | 9.62E-01 | 1.00E+00 | | 1.00E+00 | | 1.00E+00 | | 1.00E+00 | | 7.16E-02 | |  |
| GO:0061640 | cytoskeleton-dependent cytokinesis | 2.90E-02 | 9.33E-01 | 1.00E+00 | | 1.00E+00 | | 1.00E+00 | | 1.00E+00 | | 1.34E-03 | |  |
| GO:0032436 | positive regulation of proteasomal ubiquitin-dependent protein catabolic process | 2.90E-02 | 9.33E-01 | 1.00E+00 | | 1.00E+00 | | 1.00E+00 | | 1.00E+00 | | 1.60E-02 | |  |
| GO:0006637 | acyl-CoA metabolic process | 2.90E-02 | 9.33E-01 | 1.00E+00 | | 1.00E+00 | | 1.00E+00 | | 1.00E+00 | | 4.19E-02 | |  |
| GO:0035383 | thioester metabolic process | 2.90E-02 | 9.33E-01 | 1.00E+00 | | 1.00E+00 | | 1.00E+00 | | 1.00E+00 | | 4.19E-02 | |  |
| GO:0007098 | centrosome cycle | 2.90E-02 | 9.33E-01 | 1.00E+00 | | 1.00E+00 | | 1.00E+00 | | 1.00E+00 | | 4.19E-02 | |  |
| GO:1902369 | negative regulation of RNA catabolic process | 2.90E-02 | 9.62E-01 | 1.00E+00 | | 1.00E+00 | | 1.00E+00 | | 1.00E+00 | | 4.19E-02 | |  |
| GO:0034599 | cellular response to oxidative stress | 2.91E-02 | 9.74E-01 | 1.00E+00 | | 5.92E-01 | | 1.00E+00 | | 1.00E+00 | | 1.39E-02 | |  |
| GO:0060966 | regulation of gene silencing by RNA | 2.91E-02 | 9.84E-01 | 1.00E+00 | | 9.84E-01 | | 1.00E+00 | | 1.00E+00 | | 3.59E-02 | |  |
| GO:0032206 | positive regulation of telomere maintenance | 2.91E-02 | 9.33E-01 | 1.00E+00 | | 1.00E+00 | | 1.00E+00 | | 1.00E+00 | | 3.59E-02 | |  |
| GO:0007004 | telomere maintenance via telomerase | 2.91E-02 | 9.62E-01 | 1.00E+00 | | 1.00E+00 | | 1.00E+00 | | 1.00E+00 | | 3.59E-02 | |  |
| GO:0006278 | RNA-templated DNA biosynthetic process | 2.91E-02 | 9.62E-01 | 1.00E+00 | | 1.00E+00 | | 1.00E+00 | | 1.00E+00 | | 3.59E-02 | |  |
| GO:0051248 | negative regulation of protein metabolic process | 2.94E-02 | 9.33E-01 | 1.00E+00 | | 5.01E-01 | | 1.00E+00 | | 1.00E+00 | | 4.19E-02 | |  |
| GO:0140014 | mitotic nuclear division | 3.00E-02 | 9.29E-01 | 1.00E+00 | | 1.00E+00 | | 1.00E+00 | | 1.00E+00 | | 2.55E-02 | |  |
| GO:0006403 | RNA localization | 3.00E-02 | 9.29E-01 | 1.00E+00 | | 1.00E+00 | | 1.00E+00 | | 1.00E+00 | | 4.32E-02 | |  |
| GO:1904358 | positive regulation of telomere maintenance via telomere lengthening | 3.00E-02 | 9.39E-01 | 1.00E+00 | | 1.00E+00 | | 1.00E+00 | | 1.00E+00 | | 2.21E-02 | |  |
| GO:0045070 | positive regulation of viral genome replication | 3.00E-02 | 9.93E-01 | 1.00E+00 | | 1.00E+00 | | 1.00E+00 | | 1.00E+00 | | 2.21E-02 | |  |
| GO:0051301 | cell division | 3.03E-02 | 7.56E-01 | 1.00E+00 | | 1.00E+00 | | 1.00E+00 | | 1.00E+00 | | 2.43E-03 | |  |
| GO:0018210 | peptidyl-threonine modification | 3.10E-02 | 9.33E-01 | 1.00E+00 | | 9.72E-01 | | 1.00E+00 | | 1.00E+00 | | 4.96E-02 | |  |
| GO:0044087 | regulation of cellular component biogenesis | 3.11E-02 | 9.84E-01 | 1.00E+00 | | 6.46E-01 | | 7.30E-01 | | 1.00E+00 | | 2.49E-02 | |  |
| GO:0016054 | organic acid catabolic process | 3.15E-02 | 9.29E-01 | 1.00E+00 | | 1.00E+00 | | 1.00E+00 | | 1.00E+00 | | 5.51E-03 | |  |
| GO:0046578 | regulation of Ras protein signal transduction | 3.31E-02 | 9.88E-01 | 1.00E+00 | | 9.37E-01 | | 1.00E+00 | | 1.00E+00 | | 1.34E-01 | |  |
| GO:0032200 | telomere organization | 3.44E-02 | 9.29E-01 | 1.00E+00 | | 1.00E+00 | | 1.00E+00 | | 1.00E+00 | | 3.94E-02 | |  |
| GO:0043631 | RNA polyadenylation | 3.44E-02 | 9.68E-01 | 1.00E+00 | | 1.00E+00 | | 1.00E+00 | | 1.00E+00 | | 8.24E-02 | |  |
| GO:0006378 | mRNA polyadenylation | 3.44E-02 | 9.68E-01 | 1.00E+00 | | 1.00E+00 | | 1.00E+00 | | 1.00E+00 | | 8.24E-02 | |  |
| GO:0106380 | purine ribonucleotide salvage | 3.44E-02 | 9.90E-01 | 1.00E+00 | | 1.00E+00 | | 1.00E+00 | | 1.00E+00 | | 2.06E-02 | |  |
| GO:0006418 | tRNA aminoacylation for protein translation | 3.44E-02 | 9.90E-01 | 1.00E+00 | | 1.00E+00 | | 1.00E+00 | | 1.00E+00 | | 8.24E-02 | |  |
| GO:0009218 | pyrimidine ribonucleotide metabolic process | 3.44E-02 | 9.90E-01 | 1.00E+00 | | 1.00E+00 | | 1.00E+00 | | 1.00E+00 | | 8.24E-02 | |  |
| GO:0043039 | tRNA aminoacylation | 3.44E-02 | 9.90E-01 | 1.00E+00 | | 1.00E+00 | | 1.00E+00 | | 1.00E+00 | | 8.24E-02 | |  |
| GO:0043038 | amino acid activation | 3.44E-02 | 9.90E-01 | 1.00E+00 | | 1.00E+00 | | 1.00E+00 | | 1.00E+00 | | 8.24E-02 | |  |
| GO:0031647 | regulation of protein stability | 3.61E-02 | 9.29E-01 | 1.00E+00 | | 1.00E+00 | | 1.00E+00 | | 1.00E+00 | | 7.67E-02 | |  |
| GO:0009154 | purine ribonucleotide catabolic process | 3.61E-02 | 9.39E-01 | 1.00E+00 | | 1.00E+00 | | 1.00E+00 | | 1.00E+00 | | 1.06E-01 | |  |
| GO:2001233 | regulation of apoptotic signaling pathway | 3.62E-02 | 9.62E-01 | 1.00E+00 | | 9.84E-01 | | 1.00E+00 | | 1.00E+00 | | 4.19E-02 | |  |
| GO:1901657 | glycosyl compound metabolic process | 3.65E-02 | 9.29E-01 | 1.00E+00 | | 1.00E+00 | | 1.00E+00 | | 1.00E+00 | | 9.39E-03 | |  |
| GO:0010243 | response to organonitrogen compound | 3.67E-02 | 9.39E-01 | 1.00E+00 | | 8.57E-01 | | 1.00E+00 | | 1.00E+00 | | 3.75E-02 | |  |
| GO:1902745 | positive regulation of lamellipodium organization | 3.67E-02 | 9.39E-01 | 1.00E+00 | | 9.58E-01 | | 1.00E+00 | | 1.00E+00 | | 8.24E-02 | |  |
| GO:0006970 | response to osmotic stress | 3.67E-02 | 9.29E-01 | 1.00E+00 | | 1.00E+00 | | 1.00E+00 | | 1.00E+00 | | 2.57E-02 | |  |
| GO:0009116 | nucleoside metabolic process | 3.67E-02 | 9.29E-01 | 1.00E+00 | | 1.00E+00 | | 1.00E+00 | | 1.00E+00 | | 3.09E-02 | |  |
| GO:1903008 | organelle disassembly | 3.67E-02 | 9.29E-01 | 1.00E+00 | | 1.00E+00 | | 1.00E+00 | | 1.00E+00 | | 5.10E-02 | |  |
| GO:0051225 | spindle assembly | 3.67E-02 | 9.33E-01 | 1.00E+00 | | 1.00E+00 | | 1.00E+00 | | 1.00E+00 | | 7.06E-02 | |  |
| GO:0010592 | positive regulation of lamellipodium assembly | 3.67E-02 | 9.33E-01 | 1.00E+00 | | 1.00E+00 | | 1.00E+00 | | 1.00E+00 | | 8.16E-02 | |  |
| GO:0035384 | thioester biosynthetic process | 3.67E-02 | 9.39E-01 | 1.00E+00 | | 1.00E+00 | | 1.00E+00 | | 1.00E+00 | | 8.24E-02 | |  |
| GO:0071616 | acyl-CoA biosynthetic process | 3.67E-02 | 9.39E-01 | 1.00E+00 | | 1.00E+00 | | 1.00E+00 | | 1.00E+00 | | 8.24E-02 | |  |
| GO:1901984 | negative regulation of protein acetylation | 3.67E-02 | 9.39E-01 | 1.00E+00 | | 1.00E+00 | | 1.00E+00 | | 1.00E+00 | | 8.24E-02 | |  |
| GO:0060397 | growth hormone receptor signaling pathway via JAK-STAT | 3.67E-02 | 9.80E-01 | 1.00E+00 | | 1.00E+00 | | 1.00E+00 | | 1.00E+00 | | 8.16E-02 | |  |
| GO:0009170 | purine deoxyribonucleoside monophosphate metabolic process | 3.67E-02 | 9.80E-01 | 1.00E+00 | | 1.00E+00 | | 1.00E+00 | | 1.00E+00 | | 8.16E-02 | |  |
| GO:0046755 | viral budding | 3.67E-02 | 9.80E-01 | 1.00E+00 | | 1.00E+00 | | 1.00E+00 | | 1.00E+00 | | 8.16E-02 | |  |
| GO:0007183 | SMAD protein complex assembly | 3.67E-02 | 9.80E-01 | 1.00E+00 | | 1.00E+00 | | 1.00E+00 | | 1.00E+00 | | 8.16E-02 | |  |
| GO:0031468 | nuclear membrane reassembly | 3.67E-02 | 9.86E-01 | 1.00E+00 | | 1.00E+00 | | 1.00E+00 | | 1.00E+00 | | 1.69E-02 | |  |
| GO:0009083 | branched-chain amino acid catabolic process | 3.67E-02 | 9.86E-01 | 1.00E+00 | | 1.00E+00 | | 1.00E+00 | | 1.00E+00 | | 8.24E-02 | |  |
| GO:0009200 | deoxyribonucleoside triphosphate metabolic process | 3.67E-02 | 1.00E+00 | 1.00E+00 | | 1.00E+00 | | 1.00E+00 | | 1.00E+00 | | 8.16E-02 | |  |
| GO:0045176 | apical protein localization | 3.67E-02 | 1.00E+00 | 1.00E+00 | | 1.00E+00 | | 1.00E+00 | | 1.00E+00 | | 8.16E-02 | |  |
| GO:0006099 | tricarboxylic acid cycle | 3.67E-02 | 1.00E+00 | 1.00E+00 | | 1.00E+00 | | 1.00E+00 | | 1.00E+00 | | 2.94E-01 | |  |
| GO:0031053 | primary miRNA processing | 3.67E-02 | 1.00E+00 | 1.00E+00 | | 1.00E+00 | | 1.00E+00 | | 1.00E+00 | | 8.16E-02 | |  |
| GO:0010564 | regulation of cell cycle process | 3.84E-02 | 9.29E-01 | 1.00E+00 | | 1.00E+00 | | 1.00E+00 | | 1.00E+00 | | 1.47E-01 | |  |
| GO:1904903 | ESCRT III complex disassembly | 3.84E-02 | 1.00E+00 | 1.00E+00 | | 1.00E+00 | | 1.00E+00 | | 1.00E+00 | | 8.24E-02 | |  |
| GO:1904896 | ESCRT complex disassembly | 3.84E-02 | 1.00E+00 | 1.00E+00 | | 1.00E+00 | | 1.00E+00 | | 1.00E+00 | | 8.24E-02 | |  |
| GO:0090435 | protein localization to nuclear envelope | 3.84E-02 | 1.00E+00 | 1.00E+00 | | 1.00E+00 | | 1.00E+00 | | 1.00E+00 | | 8.24E-02 | |  |
| GO:0009438 | methylglyoxal metabolic process | 3.84E-02 | 1.00E+00 | 1.00E+00 | | 1.00E+00 | | 1.00E+00 | | 1.00E+00 | | 8.24E-02 | |  |
| GO:0032264 | IMP salvage | 3.84E-02 | 1.00E+00 | 1.00E+00 | | 1.00E+00 | | 1.00E+00 | | 1.00E+00 | | 8.24E-02 | |  |
| GO:0034762 | regulation of transmembrane transport | 3.92E-02 | 9.84E-01 | 1.00E+00 | | 9.35E-01 | | 1.00E+00 | | 1.00E+00 | | 4.73E-02 | |  |
| GO:0031503 | protein-containing complex localization | 3.92E-02 | 9.66E-01 | 1.00E+00 | | 9.71E-01 | | 1.00E+00 | | 1.00E+00 | | 4.19E-02 | |  |
| GO:0032456 | endocytic recycling | 4.03E-02 | 9.33E-01 | 1.00E+00 | | 1.00E+00 | | 1.00E+00 | | 1.00E+00 | | 1.41E-01 | |  |
| GO:0070646 | protein modification by small protein removal | 4.03E-02 | 9.62E-01 | 1.00E+00 | | 1.00E+00 | | 1.00E+00 | | 1.00E+00 | | 2.57E-02 | |  |
| GO:0061245 | establishment or maintenance of bipolar cell polarity | 4.03E-02 | 9.91E-01 | 1.00E+00 | | 1.00E+00 | | 1.00E+00 | | 1.00E+00 | | 1.41E-01 | |  |
| GO:0035088 | establishment or maintenance of apical/basal cell polarity | 4.03E-02 | 9.91E-01 | 1.00E+00 | | 1.00E+00 | | 1.00E+00 | | 1.00E+00 | | 1.41E-01 | |  |
| GO:0016192 | vesicle-mediated transport | 4.10E-02 | 9.39E-01 | 1.00E+00 | | 9.14E-01 | | 1.00E+00 | | 1.00E+00 | | 8.16E-02 | |  |
| GO:0042398 | cellular modified amino acid biosynthetic process | 4.27E-02 | 9.62E-01 | 1.00E+00 | | 1.00E+00 | | 1.00E+00 | | 1.00E+00 | | 1.20E-01 | |  |
| GO:0006007 | glucose catabolic process | 4.27E-02 | 9.86E-01 | 1.00E+00 | | 1.00E+00 | | 1.00E+00 | | 1.00E+00 | | 4.16E-02 | |  |
| GO:0032970 | regulation of actin filament-based process | 4.30E-02 | 9.62E-01 | 1.00E+00 | | 7.16E-01 | | 1.00E+00 | | 1.00E+00 | | 2.62E-02 | |  |
| GO:0006974 | cellular response to DNA damage stimulus | 4.31E-02 | 9.29E-01 | 1.00E+00 | | 1.00E+00 | | 1.00E+00 | | 1.00E+00 | | 4.68E-02 | |  |
| GO:0048193 | Golgi vesicle transport | 4.32E-02 | 9.62E-01 | 1.00E+00 | | 1.00E+00 | | 1.00E+00 | | 1.00E+00 | | 3.43E-02 | |  |
| GO:0009261 | ribonucleotide catabolic process | 4.35E-02 | 9.33E-01 | 1.00E+00 | | 1.00E+00 | | 1.00E+00 | | 1.00E+00 | | 1.35E-01 | |  |
| GO:0010970 | transport along microtubule | 4.43E-02 | 9.29E-01 | 1.00E+00 | | 1.00E+00 | | 1.00E+00 | | 1.00E+00 | | 2.52E-02 | |  |
| GO:0061025 | membrane fusion | 4.66E-02 | 9.29E-01 | 1.00E+00 | | 1.00E+00 | | 1.00E+00 | | 1.00E+00 | | 1.74E-03 | |  |
| GO:0044770 | cell cycle phase transition | 4.71E-02 | 9.21E-01 | 1.00E+00 | | 1.00E+00 | | 1.00E+00 | | 1.00E+00 | | 1.56E-01 | |  |
| GO:0043254 | regulation of protein-containing complex assembly | 4.71E-02 | 9.29E-01 | 1.00E+00 | | 1.00E+00 | | 1.00E+00 | | 1.00E+00 | | 2.98E-04 | |  |
| GO:0034109 | homotypic cell-cell adhesion | 4.83E-02 | 9.90E-01 | 1.00E+00 | | 7.43E-01 | | 1.00E+00 | | 1.00E+00 | | 2.71E-01 | |  |
| GO:0006790 | sulfur compound metabolic process | 4.95E-02 | 9.29E-01 | 1.00E+00 | | 1.00E+00 | | 1.00E+00 | | 1.00E+00 | | 3.26E-03 | |  |
| GO:0018209 | peptidyl-serine modification | 5.18E-02 | 9.29E-01 | 1.00E+00 | | 1.00E+00 | | 1.00E+00 | | 1.00E+00 | | 1.21E-02 | |  |
| GO:0007005 | mitochondrion organization | 5.18E-02 | 9.29E-01 | 1.00E+00 | | 1.00E+00 | | 1.00E+00 | | 1.00E+00 | | 4.23E-02 | |  |
| GO:0043101 | purine-containing compound salvage | 5.24E-02 | 9.39E-01 | 1.00E+00 | | 1.00E+00 | | 1.00E+00 | | 1.00E+00 | | 1.07E-02 | |  |
| GO:0030866 | cortical actin cytoskeleton organization | 5.24E-02 | 9.84E-01 | 1.00E+00 | | 1.00E+00 | | 1.00E+00 | | 1.00E+00 | | 4.19E-02 | |  |
| GO:0046033 | AMP metabolic process | 5.24E-02 | 9.84E-01 | 1.00E+00 | | 1.00E+00 | | 1.00E+00 | | 1.00E+00 | | 4.19E-02 | |  |
| GO:0043173 | nucleotide salvage | 5.24E-02 | 9.84E-01 | 1.00E+00 | | 1.00E+00 | | 1.00E+00 | | 1.00E+00 | | 4.19E-02 | |  |
| GO:0006259 | DNA metabolic process | 5.41E-02 | 9.29E-01 | 1.00E+00 | | 1.00E+00 | | 1.00E+00 | | 1.00E+00 | | 3.12E-02 | |  |
| GO:0009266 | response to temperature stimulus | 5.50E-02 | 9.29E-01 | 1.00E+00 | | 8.52E-01 | | 1.00E+00 | | 1.00E+00 | | 4.76E-02 | |  |
| GO:0110053 | regulation of actin filament organization | 6.33E-02 | 9.62E-01 | 1.00E+00 | | 8.52E-01 | | 1.00E+00 | | 1.00E+00 | | 1.50E-02 | |  |
| GO:0032212 | positive regulation of telomere maintenance via telomerase | 6.33E-02 | 9.33E-01 | 1.00E+00 | | 1.00E+00 | | 1.00E+00 | | 1.00E+00 | | 4.19E-02 | |  |
| GO:0007176 | regulation of epidermal growth factor-activated receptor activity | 6.33E-02 | 9.74E-01 | 1.00E+00 | | 1.00E+00 | | 1.00E+00 | | 1.00E+00 | | 4.19E-02 | |  |
| GO:0071417 | cellular response to organonitrogen compound | 6.33E-02 | 9.33E-01 | 1.00E+00 | | 1.00E+00 | | 1.00E+00 | | 1.00E+00 | | 4.31E-02 | |  |
| GO:0006446 | regulation of translational initiation | 6.50E-02 | 9.29E-01 | 1.00E+00 | | 1.00E+00 | | 1.00E+00 | | 1.00E+00 | | 3.27E-02 | |  |
| GO:0016579 | protein deubiquitination | 6.50E-02 | 9.74E-01 | 1.00E+00 | | 1.00E+00 | | 1.00E+00 | | 1.00E+00 | | 3.27E-02 | |  |
| GO:0061024 | membrane organization | 6.72E-02 | 9.33E-01 | 1.00E+00 | | 9.84E-01 | | 1.00E+00 | | 1.00E+00 | | 1.25E-02 | |  |
| GO:0010659 | cardiac muscle cell apoptotic process | 6.72E-02 | 9.33E-01 | 1.00E+00 | | 1.00E+00 | | 1.00E+00 | | 1.00E+00 | | 1.07E-02 | |  |
| GO:0045862 | positive regulation of proteolysis | 6.78E-02 | 9.39E-01 | 1.00E+00 | | 8.52E-01 | | 1.00E+00 | | 1.00E+00 | | 6.13E-03 | |  |
| GO:0032956 | regulation of actin cytoskeleton organization | 6.79E-02 | 9.35E-01 | 1.00E+00 | | 8.11E-01 | | 1.00E+00 | | 1.00E+00 | | 3.20E-02 | |  |
| GO:1901135 | carbohydrate derivative metabolic process | 6.80E-02 | 9.62E-01 | 1.00E+00 | | 1.00E+00 | | 1.00E+00 | | 1.00E+00 | | 2.20E-02 | |  |
| GO:0010658 | striated muscle cell apoptotic process | 7.00E-02 | 9.33E-01 | 1.00E+00 | | 9.84E-01 | | 1.00E+00 | | 1.00E+00 | | 1.39E-02 | |  |
| GO:0032271 | regulation of protein polymerization | 7.37E-02 | 9.77E-01 | 1.00E+00 | | 9.84E-01 | | 1.00E+00 | | 1.00E+00 | | 2.52E-02 | |  |
| GO:0046040 | IMP metabolic process | 7.37E-02 | 9.90E-01 | 1.00E+00 | | 1.00E+00 | | 1.00E+00 | | 1.00E+00 | | 4.19E-02 | |  |
| GO:1904951 | positive regulation of establishment of protein localization | 8.00E-02 | 9.33E-01 | 1.00E+00 | | 9.17E-01 | | 1.00E+00 | | 1.00E+00 | | 4.19E-02 | |  |
| GO:0048259 | regulation of receptor-mediated endocytosis | 8.37E-02 | 9.29E-01 | 1.00E+00 | | 9.58E-01 | | 1.00E+00 | | 1.00E+00 | | 5.80E-03 | |  |
| GO:0061512 | protein localization to cilium | 8.37E-02 | 9.84E-01 | 1.00E+00 | | 1.00E+00 | | 1.00E+00 | | 1.00E+00 | | 3.82E-02 | |  |
| GO:0051648 | vesicle localization | 8.65E-02 | 9.29E-01 | 1.00E+00 | | 1.00E+00 | | 1.00E+00 | | 1.00E+00 | | 9.39E-03 | |  |
| GO:0043648 | dicarboxylic acid metabolic process | 8.65E-02 | 9.29E-01 | 1.00E+00 | | 1.00E+00 | | 1.00E+00 | | 1.00E+00 | | 1.55E-02 | |  |
| GO:0051261 | protein depolymerization | 8.65E-02 | 9.76E-01 | 1.00E+00 | | 1.00E+00 | | 1.00E+00 | | 1.00E+00 | | 1.55E-02 | |  |
| GO:0000281 | mitotic cytokinesis | 8.79E-02 | 9.29E-01 | 1.00E+00 | | 1.00E+00 | | 1.00E+00 | | 1.00E+00 | | 5.37E-03 | |  |
| GO:1905897 | regulation of response to endoplasmic reticulum stress | 8.79E-02 | 9.68E-01 | 1.00E+00 | | 1.00E+00 | | 1.00E+00 | | 1.00E+00 | | 4.31E-02 | |  |
| GO:1902965 | regulation of protein localization to early endosome | 8.79E-02 | 9.74E-01 | 1.00E+00 | | 1.00E+00 | | 1.00E+00 | | 1.00E+00 | | 3.72E-02 | |  |
| GO:1902966 | positive regulation of protein localization to early endosome | 8.79E-02 | 9.74E-01 | 1.00E+00 | | 1.00E+00 | | 1.00E+00 | | 1.00E+00 | | 3.72E-02 | |  |
| GO:1905666 | regulation of protein localization to endosome | 8.79E-02 | 9.74E-01 | 1.00E+00 | | 1.00E+00 | | 1.00E+00 | | 1.00E+00 | | 3.72E-02 | |  |
| GO:1905668 | positive regulation of protein localization to endosome | 8.79E-02 | 9.74E-01 | 1.00E+00 | | 1.00E+00 | | 1.00E+00 | | 1.00E+00 | | 3.72E-02 | |  |
| GO:0051650 | establishment of vesicle localization | 8.89E-02 | 9.29E-01 | 1.00E+00 | | 1.00E+00 | | 1.00E+00 | | 1.00E+00 | | 1.07E-02 | |  |
| GO:0051258 | protein polymerization | 9.99E-02 | 9.33E-01 | 1.00E+00 | | 9.58E-01 | | 1.00E+00 | | 1.00E+00 | | 2.75E-02 | |  |
| GO:0031334 | positive regulation of protein-containing complex assembly | 1.20E-01 | 9.80E-01 | 1.00E+00 | | 9.84E-01 | | 1.00E+00 | | 1.00E+00 | | 3.20E-02 | |  |
| GO:0043085 | positive regulation of catalytic activity | 1.34E-01 | 9.39E-01 | 1.00E+00 | | 6.68E-01 | | 1.00E+00 | | 1.00E+00 | | 4.19E-02 | |  |
| GO:0006520 | cellular amino acid metabolic process | 1.34E-01 | 9.28E-01 | 1.00E+00 | | 1.00E+00 | | 1.00E+00 | | 1.00E+00 | | 2.06E-02 | |  |
| GO:1990089 | response to nerve growth factor | 1.37E-01 | 9.84E-01 | 1.00E+00 | | 5.92E-01 | | 1.00E+00 | | 1.00E+00 | | 4.36E-02 | |  |
| GO:0030832 | regulation of actin filament length | 1.63E-01 | 9.47E-01 | 1.00E+00 | | 9.02E-01 | | 1.00E+00 | | 1.00E+00 | | 3.94E-02 | |  |
| GO:0008064 | regulation of actin polymerization or depolymerization | 1.63E-01 | 9.47E-01 | 1.00E+00 | | 9.02E-01 | | 1.00E+00 | | 1.00E+00 | | 3.94E-02 | |  |
| GO:0010558 | negative regulation of macromolecule biosynthetic process | 1.68E-01 | 9.29E-01 | 1.00E+00 | | 1.00E+00 | | 1.00E+00 | | 1.00E+00 | | 3.94E-02 | |  |
| GO:0010665 | regulation of cardiac muscle cell apoptotic process | 1.70E-01 | 9.29E-01 | 1.00E+00 | | 1.00E+00 | | 1.00E+00 | | 1.00E+00 | | 3.09E-02 | |  |
| GO:0010662 | regulation of striated muscle cell apoptotic process | 1.71E-01 | 9.33E-01 | 1.00E+00 | | 9.65E-01 | | 1.00E+00 | | 1.00E+00 | | 3.82E-02 | |  |
| GO:0043066 | negative regulation of apoptotic process | 2.05E-01 | 9.29E-01 | 1.00E+00 | | 9.13E-01 | | 1.00E+00 | | 1.00E+00 | | 3.18E-02 | |  |
| GO:0008154 | actin polymerization or depolymerization | 2.05E-01 | 9.33E-01 | 1.00E+00 | | 9.84E-01 | | 1.00E+00 | | 1.00E+00 | | 4.44E-02 | |  |
| GO:2000377 | regulation of reactive oxygen species metabolic process | 2.16E-01 | 9.29E-01 | 1.00E+00 | | 1.00E+00 | | 1.00E+00 | | 1.00E+00 | | 2.06E-02 | |  |
| GO:0060548 | negative regulation of cell death | 2.21E-01 | 9.29E-01 | 1.00E+00 | | 7.43E-01 | | 1.00E+00 | | 1.00E+00 | | 2.99E-02 | |  |
| GO:0043281 | regulation of cysteine-type endopeptidase activity involved in apoptotic process | 2.25E-01 | 9.29E-01 | 1.00E+00 | | 9.19E-01 | | 1.00E+00 | | 1.00E+00 | | 2.77E-02 | |  |
| GO:0090174 | organelle membrane fusion | 2.25E-01 | 9.29E-01 | 1.00E+00 | | 1.00E+00 | | 1.00E+00 | | 1.00E+00 | | 3.50E-02 | |  |
| GO:0006906 | vesicle fusion | 2.25E-01 | 9.29E-01 | 1.00E+00 | | 1.00E+00 | | 1.00E+00 | | 1.00E+00 | | 3.50E-02 | |  |
| GO:0034063 | stress granule assembly | 2.37E-01 | 9.29E-01 | 1.00E+00 | | 1.00E+00 | | 1.00E+00 | | 1.00E+00 | | 4.19E-02 | |  |
| GO:0043069 | negative regulation of programmed cell death | 2.55E-01 | 9.29E-01 | 1.00E+00 | | 9.14E-01 | | 1.00E+00 | | 1.00E+00 | | 3.94E-02 | |  |
| GO:0002090 | regulation of receptor internalization | 2.78E-01 | 9.29E-01 | 1.00E+00 | | 9.14E-01 | | 1.00E+00 | | 1.00E+00 | | 1.35E-02 | |  |
| GO:0060271 | cilium assembly | 2.83E-01 | 9.62E-01 | 1.00E+00 | | 1.00E+00 | | 1.00E+00 | | 1.00E+00 | | 1.66E-02 | |  |
| GO:0031333 | negative regulation of protein-containing complex assembly | 3.51E-01 | 9.29E-01 | 1.00E+00 | | 9.14E-01 | | 1.00E+00 | | 1.00E+00 | | 3.50E-02 | |  |
| GO:0044782 | cilium organization | 4.11E-01 | 9.33E-01 | 1.00E+00 | | 1.00E+00 | | 1.00E+00 | | 1.00E+00 | | 3.75E-02 | |  |
| GO:0000165 | MAPK cascade | 6.16E-01 | 1.00E+00 | 1.00E+00 | | 3.18E-02 | | 8.80E-01 | | 1.00E+00 | | 7.07E-01 | |  |
| GO:0031344 | regulation of cell projection organization | 7.12E-01 | 9.93E-01 | 1.00E+00 | | 4.62E-03 | | 2.29E-01 | | 1.00E+00 | | 9.12E-01 | |  |
| GO:0120035 | regulation of plasma membrane bounded cell projection organization | 7.12E-01 | 9.91E-01 | 1.00E+00 | | 4.69E-03 | | 2.34E-01 | | 1.00E+00 | | 9.08E-01 | |  |
| GO:0034446 | substrate adhesion-dependent cell spreading | 7.12E-01 | 9.39E-01 | 1.00E+00 | | 8.90E-03 | | 9.01E-01 | | 1.00E+00 | | 8.52E-01 | |  |
| GO:0048729 | tissue morphogenesis | 7.69E-01 | 9.94E-01 | 1.00E+00 | | 2.01E-02 | | 2.88E-01 | | 1.00E+00 | | 9.12E-01 | |  |
| GO:0060429 | epithelium development | 7.73E-01 | 9.39E-01 | 1.00E+00 | | 7.69E-03 | | 5.84E-01 | | 1.00E+00 | | 9.12E-01 | |  |
| GO:0000904 | cell morphogenesis involved in differentiation | 8.48E-01 | 1.00E+00 | 1.00E+00 | | 9.03E-09 | | 8.10E-05 | | 1.00E+00 | | 9.21E-01 | |  |
| GO:0000902 | cell morphogenesis | 8.48E-01 | 9.99E-01 | 1.00E+00 | | 7.84E-08 | | 1.91E-04 | | 1.00E+00 | | 9.12E-01 | |  |
| GO:0031175 | neuron projection development | 8.48E-01 | 1.00E+00 | 1.00E+00 | | 1.98E-06 | | 8.55E-04 | | 1.00E+00 | | 9.12E-01 | |  |
| GO:0030335 | positive regulation of cell migration | 8.48E-01 | 9.80E-01 | 1.00E+00 | | 3.15E-06 | | 2.97E-01 | | 1.00E+00 | | 9.12E-01 | |  |
| GO:0048666 | neuron development | 8.48E-01 | 9.98E-01 | 1.00E+00 | | 9.19E-06 | | 7.05E-03 | | 1.00E+00 | | 9.08E-01 | |  |
| GO:0070848 | response to growth factor | 8.48E-01 | 9.84E-01 | 1.00E+00 | | 1.28E-03 | | 2.14E-01 | | 1.00E+00 | | 9.12E-01 | |  |
| GO:0010810 | regulation of cell-substrate adhesion | 8.48E-01 | 9.52E-01 | 1.00E+00 | | 2.81E-03 | | 4.65E-01 | | 1.00E+00 | | 7.30E-01 | |  |
| GO:0060322 | head development | 8.48E-01 | 9.93E-01 | 1.00E+00 | | 2.88E-03 | | 2.46E-02 | | 1.00E+00 | | 9.12E-01 | |  |
| GO:0071363 | cellular response to growth factor stimulus | 8.48E-01 | 9.86E-01 | 1.00E+00 | | 4.24E-03 | | 3.18E-01 | | 1.00E+00 | | 9.12E-01 | |  |
| GO:0031346 | positive regulation of cell projection organization | 8.48E-01 | 9.90E-01 | 1.00E+00 | | 4.62E-03 | | 1.38E-01 | | 1.00E+00 | | 9.12E-01 | |  |
| GO:0007420 | brain development | 8.48E-01 | 9.90E-01 | 1.00E+00 | | 6.10E-03 | | 4.29E-02 | | 1.00E+00 | | 9.12E-01 | |  |
| GO:0010811 | positive regulation of cell-substrate adhesion | 8.48E-01 | 9.33E-01 | 1.00E+00 | | 1.34E-02 | | 7.43E-01 | | 1.00E+00 | | 9.12E-01 | |  |
| GO:0051336 | regulation of hydrolase activity | 8.48E-01 | 9.44E-01 | 1.00E+00 | | 4.60E-02 | | 1.00E+00 | | 1.00E+00 | | 8.52E-01 | |  |
| GO:0061307 | cardiac neural crest cell differentiation involved in heart development | 8.54E-01 | 1.00E+00 | 1.00E+00 | | 4.79E-02 | | 3.89E-01 | | 1.00E+00 | | 9.40E-01 | |  |
| GO:0061308 | cardiac neural crest cell development involved in heart development | 8.54E-01 | 1.00E+00 | 1.00E+00 | | 4.79E-02 | | 3.89E-01 | | 1.00E+00 | | 9.40E-01 | |  |
| GO:0001558 | regulation of cell growth | 8.55E-01 | 9.90E-01 | 1.00E+00 | | 4.86E-02 | | 2.55E-01 | | 1.00E+00 | | 9.40E-01 | |  |
| GO:0031589 | cell-substrate adhesion | 9.06E-01 | 9.84E-01 | 1.00E+00 | | 6.45E-05 | | 7.58E-02 | | 1.00E+00 | | 9.12E-01 | |  |
| GO:0009611 | response to wounding | 9.06E-01 | 9.84E-01 | 1.00E+00 | | 1.75E-03 | | 1.23E-01 | | 1.00E+00 | | 9.40E-01 | |  |
| GO:0034097 | response to cytokine | 9.06E-01 | 9.99E-01 | 1.00E+00 | | 2.15E-03 | | 1.71E-01 | | 1.00E+00 | | 9.93E-01 | |  |
| GO:0030900 | forebrain development | 9.06E-01 | 9.90E-01 | 1.00E+00 | | 1.38E-02 | | 1.02E-01 | | 1.00E+00 | | 9.69E-01 | |  |
| GO:0007417 | central nervous system development | 9.06E-01 | 9.79E-01 | 1.00E+00 | | 1.58E-02 | | 1.91E-02 | | 1.00E+00 | | 9.12E-01 | |  |
| GO:0042063 | gliogenesis | 9.06E-01 | 9.94E-01 | 1.00E+00 | | 2.02E-01 | | 3.58E-02 | | 1.00E+00 | | 9.12E-01 | |  |
| GO:0051983 | regulation of chromosome segregation | 9.06E-01 | 1.94E-02 | 1.00E+00 | | 1.00E+00 | | 1.00E+00 | | 3.68E-01 | | 9.12E-01 | |  |
| GO:0043408 | regulation of MAPK cascade | 9.06E-01 | 1.00E+00 | 1.00E+00 | | 5.73E-03 | | 5.78E-01 | | 1.00E+00 | | 9.19E-01 | |  |
| GO:0071345 | cellular response to cytokine stimulus | 9.08E-01 | 9.99E-01 | 1.00E+00 | | 1.35E-02 | | 1.83E-01 | | 1.00E+00 | | 9.93E-01 | |  |
| GO:2000147 | positive regulation of cell motility | 9.21E-01 | 9.80E-01 | 1.00E+00 | | 3.15E-06 | | 2.47E-01 | | 1.00E+00 | | 9.12E-01 | |  |
| GO:1902533 | positive regulation of intracellular signal transduction | 9.21E-01 | 9.90E-01 | 1.00E+00 | | 4.44E-02 | | 9.84E-01 | | 1.00E+00 | | 9.40E-01 | |  |
| GO:0034612 | response to tumor necrosis factor | 9.32E-01 | 9.91E-01 | 1.00E+00 | | 1.55E-02 | | 2.12E-01 | | 1.00E+00 | | 9.56E-01 | |  |
| GO:0071356 | cellular response to tumor necrosis factor | 9.36E-01 | 9.97E-01 | 1.00E+00 | | 1.32E-02 | | 1.83E-01 | | 1.00E+00 | | 9.72E-01 | |  |
| GO:0010975 | regulation of neuron projection development | 9.47E-01 | 9.94E-01 | 1.00E+00 | | 6.11E-04 | | 2.41E-02 | | 1.00E+00 | | 9.72E-01 | |  |
| GO:0016049 | cell growth | 9.47E-01 | 9.91E-01 | 1.00E+00 | | 3.80E-03 | | 1.41E-01 | | 1.00E+00 | | 9.45E-01 | |  |
| GO:0048858 | cell projection morphogenesis | 9.59E-01 | 1.00E+00 | 1.00E+00 | | 2.12E-07 | | 3.12E-07 | | 1.00E+00 | | 9.93E-01 | |  |
| GO:0032990 | cell part morphogenesis | 9.59E-01 | 1.00E+00 | 1.00E+00 | | 2.77E-07 | | 4.54E-07 | | 1.00E+00 | | 9.81E-01 | |  |
| GO:0048667 | cell morphogenesis involved in neuron differentiation | 9.59E-01 | 1.00E+00 | 1.00E+00 | | 2.77E-07 | | 1.36E-06 | | 1.00E+00 | | 1.00E+00 | |  |
| GO:0040017 | positive regulation of locomotion | 9.59E-01 | 9.86E-01 | 1.00E+00 | | 6.08E-07 | | 1.67E-01 | | 1.00E+00 | | 9.40E-01 | |  |
| GO:0048589 | developmental growth | 9.59E-01 | 9.91E-01 | 1.00E+00 | | 7.55E-04 | | 2.60E-02 | | 1.00E+00 | | 9.73E-01 | |  |
| GO:0060284 | regulation of cell development | 9.59E-01 | 9.90E-01 | 1.00E+00 | | 9.18E-04 | | 3.82E-02 | | 1.00E+00 | | 9.72E-01 | |  |
| GO:0048013 | ephrin receptor signaling pathway | 9.59E-01 | 9.90E-01 | 1.00E+00 | | 1.54E-02 | | 4.29E-02 | | 1.00E+00 | | 9.40E-01 | |  |
| GO:0120039 | plasma membrane bounded cell projection morphogenesis | 9.63E-01 | 1.00E+00 | 1.00E+00 | | 1.70E-07 | | 1.87E-07 | | 1.00E+00 | | 9.93E-01 | |  |
| GO:0048812 | neuron projection morphogenesis | 9.63E-01 | 1.00E+00 | 1.00E+00 | | 1.87E-07 | | 3.81E-07 | | 1.00E+00 | | 9.93E-01 | |  |
| GO:0032989 | cellular component morphogenesis | 9.63E-01 | 1.00E+00 | 1.00E+00 | | 2.52E-06 | | 3.81E-07 | | 1.00E+00 | | 9.93E-01 | |  |
| GO:0048588 | developmental cell growth | 9.63E-01 | 9.98E-01 | 1.00E+00 | | 2.24E-03 | | 1.83E-01 | | 1.00E+00 | | 9.40E-01 | |  |
| GO:0050900 | leukocyte migration | 9.63E-01 | 9.90E-01 | 1.00E+00 | | 8.93E-03 | | 2.52E-01 | | 1.00E+00 | | 9.93E-01 | |  |
| GO:0034330 | cell junction organization | 9.63E-01 | 1.00E+00 | 1.00E+00 | | 1.50E-02 | | 4.32E-04 | | 1.00E+00 | | 1.00E+00 | |  |
| GO:0021954 | central nervous system neuron development | 9.63E-01 | 9.74E-01 | 1.00E+00 | | 4.86E-02 | | 4.30E-01 | | 1.00E+00 | | 9.55E-01 | |  |
| GO:0010717 | regulation of epithelial to mesenchymal transition | 9.63E-01 | 9.98E-01 | 1.00E+00 | | 9.06E-02 | | 4.67E-02 | | 1.00E+00 | | 9.93E-01 | |  |
| GO:0052547 | regulation of peptidase activity | 9.71E-01 | 9.39E-01 | 1.00E+00 | | 2.74E-02 | | 1.00E+00 | | 1.00E+00 | | 9.12E-01 | |  |
| GO:1990868 | response to chemokine | 9.78E-01 | 9.94E-01 | 1.00E+00 | | 3.70E-03 | | 4.44E-01 | | 1.00E+00 | | 9.93E-01 | |  |
| GO:1990869 | cellular response to chemokine | 9.78E-01 | 9.94E-01 | 1.00E+00 | | 3.70E-03 | | 4.44E-01 | | 1.00E+00 | | 9.93E-01 | |  |
| GO:0030593 | neutrophil chemotaxis | 9.83E-01 | 9.90E-01 | 1.00E+00 | | 1.79E-02 | | 5.63E-01 | | 1.00E+00 | | 1.00E+00 | |  |
| GO:0040007 | growth | 9.84E-01 | 9.84E-01 | 1.00E+00 | | 3.09E-03 | | 4.20E-02 | | 1.00E+00 | | 9.93E-01 | |  |
| GO:0071621 | granulocyte chemotaxis | 9.86E-01 | 9.90E-01 | 1.00E+00 | | 4.26E-03 | | 5.78E-01 | | 1.00E+00 | | 9.97E-01 | |  |
| GO:0070098 | chemokine-mediated signaling pathway | 9.86E-01 | 9.92E-01 | 1.00E+00 | | 1.22E-02 | | 5.89E-01 | | 1.00E+00 | | 1.00E+00 | |  |
| GO:0007610 | behavior | 9.86E-01 | 9.91E-01 | 1.00E+00 | | 1.50E-01 | | 1.41E-02 | | 1.00E+00 | | 1.00E+00 | |  |
| GO:1990266 | neutrophil migration | 9.90E-01 | 9.90E-01 | 1.00E+00 | | 5.35E-03 | | 5.45E-01 | | 1.00E+00 | | 1.00E+00 | |  |
| GO:0060485 | mesenchyme development | 9.90E-01 | 1.00E+00 | 1.00E+00 | | 2.94E-02 | | 4.68E-03 | | 1.00E+00 | | 9.93E-01 | |  |
| GO:0031345 | negative regulation of cell projection organization | 9.90E-01 | 9.74E-01 | 1.00E+00 | | 4.20E-02 | | 3.26E-01 | | 1.00E+00 | | 1.00E+00 | |  |
| GO:0048762 | mesenchymal cell differentiation | 9.90E-01 | 1.00E+00 | 1.00E+00 | | 8.10E-02 | | 5.72E-03 | | 1.00E+00 | | 1.00E+00 | |  |
| GO:0010812 | negative regulation of cell-substrate adhesion | 9.94E-01 | 9.80E-01 | 1.00E+00 | | 2.44E-02 | | 5.79E-01 | | 1.00E+00 | | 9.40E-01 | |  |
| GO:0072359 | circulatory system development | 9.96E-01 | 9.86E-01 | 1.00E+00 | | 3.79E-06 | | 4.15E-03 | | 1.00E+00 | | 9.93E-01 | |  |
| GO:0034329 | cell junction assembly | 9.99E-01 | 1.00E+00 | 1.00E+00 | | 9.06E-02 | | 2.65E-04 | | 1.00E+00 | | 1.00E+00 | |  |
| GO:0007155 | cell adhesion | 1.00E+00 | 9.98E-01 | 1.00E+00 | | 2.38E-18 | | 1.99E-12 | | 1.00E+00 | | 1.00E+00 | |  |
| GO:0040011 | locomotion | 1.00E+00 | 9.90E-01 | 1.00E+00 | | 2.12E-13 | | 8.80E-09 | | 1.00E+00 | | 1.00E+00 | |  |
| GO:0042330 | taxis | 1.00E+00 | 9.95E-01 | 1.00E+00 | | 6.25E-13 | | 2.97E-08 | | 1.00E+00 | | 1.00E+00 | |  |
| GO:0006935 | chemotaxis | 1.00E+00 | 9.95E-01 | 1.00E+00 | | 6.25E-13 | | 2.97E-08 | | 1.00E+00 | | 1.00E+00 | |  |
| GO:0097485 | neuron projection guidance | 1.00E+00 | 9.99E-01 | 1.00E+00 | | 1.80E-09 | | 2.63E-10 | | 1.00E+00 | | 1.00E+00 | |  |
| GO:0007411 | axon guidance | 1.00E+00 | 9.99E-01 | 1.00E+00 | | 1.80E-09 | | 2.63E-10 | | 1.00E+00 | | 1.00E+00 | |  |
| GO:0061564 | axon development | 1.00E+00 | 1.00E+00 | 1.00E+00 | | 2.16E-08 | | 4.08E-08 | | 1.00E+00 | | 1.00E+00 | |  |
| GO:0007409 | axonogenesis | 1.00E+00 | 1.00E+00 | 1.00E+00 | | 2.64E-08 | | 2.23E-08 | | 1.00E+00 | | 1.00E+00 | |  |
| GO:0040012 | regulation of locomotion | 1.00E+00 | 9.84E-01 | 1.00E+00 | | 2.64E-08 | | 1.47E-04 | | 1.00E+00 | | 1.00E+00 | |  |
| GO:0030334 | regulation of cell migration | 1.00E+00 | 9.84E-01 | 1.00E+00 | | 3.52E-08 | | 6.56E-04 | | 1.00E+00 | | 9.91E-01 | |  |
| GO:0051241 | negative regulation of multicellular organismal process | 1.00E+00 | 9.33E-01 | 1.00E+00 | | 4.62E-08 | | 1.74E-04 | | 1.00E+00 | | 1.00E+00 | |  |
| GO:2000145 | regulation of cell motility | 1.00E+00 | 9.86E-01 | 1.00E+00 | | 5.14E-08 | | 3.98E-04 | | 1.00E+00 | | 9.93E-01 | |  |
| GO:0030198 | extracellular matrix organization | 1.00E+00 | 9.94E-01 | 1.00E+00 | | 2.10E-06 | | 1.24E-07 | | 1.00E+00 | | 1.00E+00 | |  |
| GO:0043062 | extracellular structure organization | 1.00E+00 | 9.94E-01 | 1.00E+00 | | 2.10E-06 | | 1.24E-07 | | 1.00E+00 | | 1.00E+00 | |  |
| GO:0001525 | angiogenesis | 1.00E+00 | 9.94E-01 | 1.00E+00 | | 2.10E-06 | | 4.45E-04 | | 1.00E+00 | | 9.93E-01 | |  |
| GO:0045229 | external encapsulating structure organization | 1.00E+00 | 9.94E-01 | 1.00E+00 | | 2.52E-06 | | 8.81E-08 | | 1.00E+00 | | 1.00E+00 | |  |
| GO:0022603 | regulation of anatomical structure morphogenesis | 1.00E+00 | 9.95E-01 | 1.00E+00 | | 3.16E-06 | | 3.33E-03 | | 1.00E+00 | | 9.89E-01 | |  |
| GO:0001944 | vasculature development | 1.00E+00 | 9.90E-01 | 1.00E+00 | | 3.73E-06 | | 4.58E-04 | | 1.00E+00 | | 9.93E-01 | |  |
| GO:0001568 | blood vessel development | 1.00E+00 | 9.93E-01 | 1.00E+00 | | 1.60E-05 | | 4.59E-04 | | 1.00E+00 | | 9.93E-01 | |  |
| GO:0048514 | blood vessel morphogenesis | 1.00E+00 | 9.90E-01 | 1.00E+00 | | 3.00E-05 | | 7.74E-04 | | 1.00E+00 | | 1.00E+00 | |  |
| GO:0051960 | regulation of nervous system development | 1.00E+00 | 9.94E-01 | 1.00E+00 | | 3.37E-05 | | 4.98E-06 | | 1.00E+00 | | 1.00E+00 | |  |
| GO:0030155 | regulation of cell adhesion | 1.00E+00 | 9.90E-01 | 1.00E+00 | | 4.41E-05 | | 1.40E-03 | | 1.00E+00 | | 9.75E-01 | |  |
| GO:0051240 | positive regulation of multicellular organismal process | 1.00E+00 | 9.80E-01 | 1.00E+00 | | 4.56E-05 | | 8.55E-04 | | 1.00E+00 | | 1.00E+00 | |  |
| GO:0007167 | enzyme-linked receptor protein signaling pathway | 1.00E+00 | 9.80E-01 | 1.00E+00 | | 4.65E-05 | | 1.31E-02 | | 1.00E+00 | | 9.91E-01 | |  |
| GO:0070372 | regulation of ERK1 and ERK2 cascade | 1.00E+00 | 1.00E+00 | 1.00E+00 | | 8.46E-05 | | 2.03E-02 | | 1.00E+00 | | 1.00E+00 | |  |
| GO:0035295 | tube development | 1.00E+00 | 9.84E-01 | 1.00E+00 | | 8.46E-05 | | 3.47E-02 | | 1.00E+00 | | 9.84E-01 | |  |
| GO:0032102 | negative regulation of response to external stimulus | 1.00E+00 | 9.62E-01 | 1.00E+00 | | 9.25E-05 | | 1.23E-03 | | 1.00E+00 | | 1.00E+00 | |  |
| GO:0002040 | sprouting angiogenesis | 1.00E+00 | 9.92E-01 | 1.00E+00 | | 9.25E-05 | | 5.61E-03 | | 1.00E+00 | | 1.00E+00 | |  |
| GO:2000026 | regulation of multicellular organismal development | 1.00E+00 | 9.92E-01 | 1.00E+00 | | 1.04E-04 | | 8.02E-06 | | 1.00E+00 | | 1.00E+00 | |  |
| GO:0008284 | positive regulation of cell population proliferation | 1.00E+00 | 9.95E-01 | 1.00E+00 | | 1.33E-04 | | 6.88E-03 | | 1.00E+00 | | 9.93E-01 | |  |
| GO:0048771 | tissue remodeling | 1.00E+00 | 9.94E-01 | 1.00E+00 | | 1.43E-04 | | 1.43E-04 | | 1.00E+00 | | 1.00E+00 | |  |
| GO:0090287 | regulation of cellular response to growth factor stimulus | 1.00E+00 | 9.84E-01 | 1.00E+00 | | 1.72E-04 | | 9.08E-03 | | 1.00E+00 | | 1.00E+00 | |  |
| GO:0048646 | anatomical structure formation involved in morphogenesis | 1.00E+00 | 9.98E-01 | 1.00E+00 | | 1.75E-04 | | 7.74E-04 | | 1.00E+00 | | 9.93E-01 | |  |
| GO:0070371 | ERK1 and ERK2 cascade | 1.00E+00 | 1.00E+00 | 1.00E+00 | | 1.92E-04 | | 4.29E-02 | | 1.00E+00 | | 1.00E+00 | |  |
| GO:0035239 | tube morphogenesis | 1.00E+00 | 9.90E-01 | 1.00E+00 | | 2.67E-04 | | 2.60E-02 | | 1.00E+00 | | 9.55E-01 | |  |
| GO:0001817 | regulation of cytokine production | 1.00E+00 | 9.98E-01 | 1.00E+00 | | 2.73E-04 | | 8.89E-02 | | 1.00E+00 | | 1.00E+00 | |  |
| GO:0050770 | regulation of axonogenesis | 1.00E+00 | 9.94E-01 | 1.00E+00 | | 3.44E-04 | | 5.87E-04 | | 1.00E+00 | | 1.00E+00 | |  |
| GO:0098609 | cell-cell adhesion | 1.00E+00 | 9.90E-01 | 1.00E+00 | | 3.80E-04 | | 1.10E-03 | | 1.00E+00 | | 1.00E+00 | |  |
| GO:0050771 | negative regulation of axonogenesis | 1.00E+00 | 1.00E+00 | 1.00E+00 | | 3.90E-04 | | 1.16E-03 | | 1.00E+00 | | 1.00E+00 | |  |
| GO:0006954 | inflammatory response | 1.00E+00 | 9.95E-01 | 1.00E+00 | | 3.90E-04 | | 1.23E-03 | | 1.00E+00 | | 1.00E+00 | |  |
| GO:0022617 | extracellular matrix disassembly | 1.00E+00 | 9.84E-01 | 1.00E+00 | | 4.27E-04 | | 3.36E-02 | | 1.00E+00 | | 1.00E+00 | |  |
| GO:0050919 | negative chemotaxis | 1.00E+00 | 9.94E-01 | 1.00E+00 | | 4.65E-04 | | 4.15E-03 | | 1.00E+00 | | 1.00E+00 | |  |
| GO:0060326 | cell chemotaxis | 1.00E+00 | 9.84E-01 | 1.00E+00 | | 4.78E-04 | | 1.49E-01 | | 1.00E+00 | | 1.00E+00 | |  |
| GO:0030509 | BMP signaling pathway | 1.00E+00 | 9.94E-01 | 1.00E+00 | | 5.85E-04 | | 1.25E-02 | | 1.00E+00 | | 1.00E+00 | |  |
| GO:0001816 | cytokine production | 1.00E+00 | 9.94E-01 | 1.00E+00 | | 7.15E-04 | | 8.76E-02 | | 1.00E+00 | | 1.00E+00 | |  |
| GO:0060560 | developmental growth involved in morphogenesis | 1.00E+00 | 9.90E-01 | 1.00E+00 | | 7.21E-04 | | 4.76E-02 | | 1.00E+00 | | 9.72E-01 | |  |
| GO:0009887 | animal organ morphogenesis | 1.00E+00 | 9.33E-01 | 1.00E+00 | | 7.96E-04 | | 4.58E-03 | | 1.00E+00 | | 1.00E+00 | |  |
| GO:0070374 | positive regulation of ERK1 and ERK2 cascade | 1.00E+00 | 9.92E-01 | 1.00E+00 | | 9.15E-04 | | 1.45E-01 | | 1.00E+00 | | 1.00E+00 | |  |
| GO:0050920 | regulation of chemotaxis | 1.00E+00 | 9.62E-01 | 1.00E+00 | | 9.98E-04 | | 3.21E-01 | | 1.00E+00 | | 1.00E+00 | |  |
| GO:0031290 | retinal ganglion cell axon guidance | 1.00E+00 | 9.84E-01 | 1.00E+00 | | 1.07E-03 | | 1.22E-03 | | 1.00E+00 | | 1.00E+00 | |  |
| GO:0097530 | granulocyte migration | 1.00E+00 | 9.90E-01 | 1.00E+00 | | 1.19E-03 | | 4.85E-01 | | 1.00E+00 | | 1.00E+00 | |  |
| GO:0050767 | regulation of neurogenesis | 1.00E+00 | 9.86E-01 | 1.00E+00 | | 1.25E-03 | | 9.90E-04 | | 1.00E+00 | | 1.00E+00 | |  |
| GO:0060309 | elastin catabolic process | 1.00E+00 | 1.00E+00 | 1.00E+00 | | 1.34E-03 | | 1.38E-01 | | 1.00E+00 | | 1.00E+00 | |  |
| GO:0048678 | response to axon injury | 1.00E+00 | 9.62E-01 | 1.00E+00 | | 1.42E-03 | | 3.89E-01 | | 1.00E+00 | | 1.00E+00 | |  |
| GO:0007162 | negative regulation of cell adhesion | 1.00E+00 | 9.94E-01 | 1.00E+00 | | 1.50E-03 | | 2.13E-03 | | 1.00E+00 | | 1.00E+00 | |  |
| GO:0090092 | regulation of transmembrane receptor protein serine/threonine kinase signaling pathway | 1.00E+00 | 9.84E-01 | 1.00E+00 | | 1.50E-03 | | 2.64E-02 | | 1.00E+00 | | 1.00E+00 | |  |
| GO:0030517 | negative regulation of axon extension | 1.00E+00 | 9.93E-01 | 1.00E+00 | | 1.65E-03 | | 2.87E-03 | | 1.00E+00 | | 1.00E+00 | |  |
| GO:0007565 | female pregnancy | 1.00E+00 | 9.39E-01 | 1.00E+00 | | 1.76E-03 | | 1.37E-01 | | 1.00E+00 | | 1.00E+00 | |  |
| GO:0030510 | regulation of BMP signaling pathway | 1.00E+00 | 9.91E-01 | 1.00E+00 | | 1.87E-03 | | 7.15E-03 | | 1.00E+00 | | 1.00E+00 | |  |
| GO:0044706 | multi-multicellular organism process | 1.00E+00 | 9.39E-01 | 1.00E+00 | | 1.87E-03 | | 4.47E-02 | | 1.00E+00 | | 1.00E+00 | |  |
| GO:0030574 | collagen catabolic process | 1.00E+00 | 9.84E-01 | 1.00E+00 | | 2.07E-03 | | 7.06E-02 | | 1.00E+00 | | 1.00E+00 | |  |
| GO:0044703 | multi-organism reproductive process | 1.00E+00 | 9.33E-01 | 1.00E+00 | | 2.08E-03 | | 7.54E-02 | | 1.00E+00 | | 1.00E+00 | |  |
| GO:0097529 | myeloid leukocyte migration | 1.00E+00 | 9.80E-01 | 1.00E+00 | | 2.08E-03 | | 4.52E-01 | | 1.00E+00 | | 1.00E+00 | |  |
| GO:0008038 | neuron recognition | 1.00E+00 | 1.00E+00 | 1.00E+00 | | 2.10E-03 | | 2.51E-04 | | 1.00E+00 | | 1.00E+00 | |  |
| GO:0050922 | negative regulation of chemotaxis | 1.00E+00 | 9.90E-01 | 1.00E+00 | | 2.10E-03 | | 2.33E-03 | | 1.00E+00 | | 1.00E+00 | |  |
| GO:0071772 | response to BMP | 1.00E+00 | 9.94E-01 | 1.00E+00 | | 2.15E-03 | | 6.68E-03 | | 1.00E+00 | | 1.00E+00 | |  |
| GO:0071773 | cellular response to BMP stimulus | 1.00E+00 | 9.94E-01 | 1.00E+00 | | 2.15E-03 | | 6.68E-03 | | 1.00E+00 | | 1.00E+00 | |  |
| GO:0032963 | collagen metabolic process | 1.00E+00 | 9.39E-01 | 1.00E+00 | | 2.15E-03 | | 8.25E-03 | | 1.00E+00 | | 1.00E+00 | |  |
| GO:0007566 | embryo implantation | 1.00E+00 | 9.66E-01 | 1.00E+00 | | 2.15E-03 | | 4.60E-01 | | 1.00E+00 | | 1.00E+00 | |  |
| GO:0035272 | exocrine system development | 1.00E+00 | 1.00E+00 | 1.00E+00 | | 2.22E-03 | | 1.19E-01 | | 1.00E+00 | | 1.00E+00 | |  |
| GO:0001818 | negative regulation of cytokine production | 1.00E+00 | 9.98E-01 | 1.00E+00 | | 2.46E-03 | | 2.10E-02 | | 1.00E+00 | | 1.00E+00 | |  |
| GO:1901890 | positive regulation of cell junction assembly | 1.00E+00 | 9.98E-01 | 1.00E+00 | | 2.61E-03 | | 4.59E-04 | | 1.00E+00 | | 1.00E+00 | |  |
| GO:0030595 | leukocyte chemotaxis | 1.00E+00 | 9.77E-01 | 1.00E+00 | | 3.09E-03 | | 4.44E-01 | | 1.00E+00 | | 1.00E+00 | |  |
| GO:0032101 | regulation of response to external stimulus | 1.00E+00 | 9.35E-01 | 1.00E+00 | | 3.15E-03 | | 1.54E-01 | | 1.00E+00 | | 1.00E+00 | |  |
| GO:0009967 | positive regulation of signal transduction | 1.00E+00 | 9.90E-01 | 1.00E+00 | | 3.42E-03 | | 6.94E-01 | | 1.00E+00 | | 9.93E-01 | |  |
| GO:0001819 | positive regulation of cytokine production | 1.00E+00 | 9.90E-01 | 1.00E+00 | | 3.50E-03 | | 2.07E-01 | | 1.00E+00 | | 1.00E+00 | |  |
| GO:0010977 | negative regulation of neuron projection development | 1.00E+00 | 9.86E-01 | 1.00E+00 | | 3.70E-03 | | 1.00E-01 | | 1.00E+00 | | 1.00E+00 | |  |
| GO:0098742 | cell-cell adhesion via plasma-membrane adhesion molecules | 1.00E+00 | 9.33E-01 | 1.00E+00 | | 3.98E-03 | | 9.15E-06 | | 1.00E+00 | | 1.00E+00 | |  |
| GO:0051094 | positive regulation of developmental process | 1.00E+00 | 9.29E-01 | 1.00E+00 | | 4.08E-03 | | 1.02E-02 | | 1.00E+00 | | 1.00E+00 | |  |
| GO:0050768 | negative regulation of neurogenesis | 1.00E+00 | 9.84E-01 | 1.00E+00 | | 4.47E-03 | | 1.34E-02 | | 1.00E+00 | | 1.00E+00 | |  |
| GO:0007423 | sensory organ development | 1.00E+00 | 9.90E-01 | 1.00E+00 | | 4.62E-03 | | 3.19E-04 | | 1.00E+00 | | 1.00E+00 | |  |
| GO:0070570 | regulation of neuron projection regeneration | 1.00E+00 | 1.00E+00 | 1.00E+00 | | 4.62E-03 | | 2.06E-01 | | 1.00E+00 | | 1.00E+00 | |  |
| GO:0001764 | neuron migration | 1.00E+00 | 9.74E-01 | 1.00E+00 | | 4.64E-03 | | 1.50E-02 | | 1.00E+00 | | 9.93E-01 | |  |
| GO:0061309 | cardiac neural crest cell development involved in outflow tract morphogenesis | 1.00E+00 | 1.00E+00 | 1.00E+00 | | 4.76E-03 | | 7.05E-02 | | 1.00E+00 | | 1.00E+00 | |  |
| GO:1903054 | negative regulation of extracellular matrix organization | 1.00E+00 | 9.91E-01 | 1.00E+00 | | 4.98E-03 | | 1.72E-01 | | 1.00E+00 | | 1.00E+00 | |  |
| GO:0048880 | sensory system development | 1.00E+00 | 9.74E-01 | 1.00E+00 | | 5.25E-03 | | 1.47E-04 | | 1.00E+00 | | 1.00E+00 | |  |
| GO:0060310 | regulation of elastin catabolic process | 1.00E+00 | 1.00E+00 | 1.00E+00 | | 5.81E-03 | | 5.97E-01 | | 1.00E+00 | | 1.00E+00 | |  |
| GO:0060311 | negative regulation of elastin catabolic process | 1.00E+00 | 1.00E+00 | 1.00E+00 | | 5.81E-03 | | 5.97E-01 | | 1.00E+00 | | 1.00E+00 | |  |
| GO:0060313 | negative regulation of blood vessel remodeling | 1.00E+00 | 1.00E+00 | 1.00E+00 | | 5.81E-03 | | 5.97E-01 | | 1.00E+00 | | 1.00E+00 | |  |
| GO:0010711 | negative regulation of collagen catabolic process | 1.00E+00 | 1.00E+00 | 1.00E+00 | | 5.81E-03 | | 5.97E-01 | | 1.00E+00 | | 1.00E+00 | |  |
| GO:0061387 | regulation of extent of cell growth | 1.00E+00 | 9.94E-01 | 1.00E+00 | | 5.89E-03 | | 4.10E-02 | | 1.00E+00 | | 1.00E+00 | |  |
| GO:1902284 | neuron projection extension involved in neuron projection guidance | 1.00E+00 | 9.68E-01 | 1.00E+00 | | 6.24E-03 | | 1.44E-01 | | 1.00E+00 | | 1.00E+00 | |  |
| GO:0048846 | axon extension involved in axon guidance | 1.00E+00 | 9.68E-01 | 1.00E+00 | | 6.24E-03 | | 1.44E-01 | | 1.00E+00 | | 1.00E+00 | |  |
| GO:0030514 | negative regulation of BMP signaling pathway | 1.00E+00 | 9.80E-01 | 1.00E+00 | | 6.95E-03 | | 2.42E-01 | | 1.00E+00 | | 1.00E+00 | |  |
| GO:0010721 | negative regulation of cell development | 1.00E+00 | 9.62E-01 | 1.00E+00 | | 7.38E-03 | | 2.70E-02 | | 1.00E+00 | | 1.00E+00 | |  |
| GO:0150063 | visual system development | 1.00E+00 | 9.62E-01 | 1.00E+00 | | 7.69E-03 | | 9.30E-04 | | 1.00E+00 | | 1.00E+00 | |  |
| GO:0001654 | eye development | 1.00E+00 | 9.62E-01 | 1.00E+00 | | 7.69E-03 | | 9.30E-04 | | 1.00E+00 | | 1.00E+00 | |  |
| GO:0051541 | elastin metabolic process | 1.00E+00 | 9.90E-01 | 1.00E+00 | | 8.05E-03 | | 3.89E-01 | | 1.00E+00 | | 1.00E+00 | |  |
| GO:0045597 | positive regulation of cell differentiation | 1.00E+00 | 9.29E-01 | 1.00E+00 | | 8.24E-03 | | 2.08E-01 | | 1.00E+00 | | 9.93E-01 | |  |
| GO:0031102 | neuron projection regeneration | 1.00E+00 | 9.62E-01 | 1.00E+00 | | 8.68E-03 | | 4.35E-01 | | 1.00E+00 | | 1.00E+00 | |  |
| GO:0007431 | salivary gland development | 1.00E+00 | 1.00E+00 | 1.00E+00 | | 8.72E-03 | | 4.90E-01 | | 1.00E+00 | | 1.00E+00 | |  |
| GO:0051961 | negative regulation of nervous system development | 1.00E+00 | 9.86E-01 | 1.00E+00 | | 8.90E-03 | | 1.15E-02 | | 1.00E+00 | | 1.00E+00 | |  |
| GO:0009617 | response to bacterium | 1.00E+00 | 9.74E-01 | 1.00E+00 | | 8.90E-03 | | 1.54E-02 | | 1.00E+00 | | 1.00E+00 | |  |
| GO:0050918 | positive chemotaxis | 1.00E+00 | 9.80E-01 | 1.00E+00 | | 8.90E-03 | | 6.31E-01 | | 1.00E+00 | | 1.00E+00 | |  |
| GO:0050772 | positive regulation of axonogenesis | 1.00E+00 | 9.84E-01 | 1.00E+00 | | 9.16E-03 | | 2.95E-02 | | 1.00E+00 | | 1.00E+00 | |  |
| GO:0034103 | regulation of tissue remodeling | 1.00E+00 | 9.94E-01 | 1.00E+00 | | 9.87E-03 | | 6.68E-03 | | 1.00E+00 | | 1.00E+00 | |  |
| GO:0090101 | negative regulation of transmembrane receptor protein serine/threonine kinase signaling pathway | 1.00E+00 | 9.84E-01 | 1.00E+00 | | 1.04E-02 | | 1.38E-01 | | 1.00E+00 | | 9.93E-01 | |  |
| GO:0050673 | epithelial cell proliferation | 1.00E+00 | 9.29E-01 | 1.00E+00 | | 1.06E-02 | | 1.83E-01 | | 1.00E+00 | | 1.00E+00 | |  |
| GO:0048679 | regulation of axon regeneration | 1.00E+00 | 1.00E+00 | 1.00E+00 | | 1.08E-02 | | 2.65E-01 | | 1.00E+00 | | 1.00E+00 | |  |
| GO:0110148 | biomineralization | 1.00E+00 | 9.62E-01 | 1.00E+00 | | 1.09E-02 | | 1.23E-01 | | 1.00E+00 | | 1.00E+00 | |  |
| GO:0051962 | positive regulation of nervous system development | 1.00E+00 | 9.91E-01 | 1.00E+00 | | 1.11E-02 | | 1.47E-04 | | 1.00E+00 | | 1.00E+00 | |  |
| GO:0106030 | neuron projection fasciculation | 1.00E+00 | 1.00E+00 | 1.00E+00 | | 1.11E-02 | | 1.51E-02 | | 1.00E+00 | | 1.00E+00 | |  |
| GO:0007413 | axonal fasciculation | 1.00E+00 | 1.00E+00 | 1.00E+00 | | 1.11E-02 | | 1.51E-02 | | 1.00E+00 | | 1.00E+00 | |  |
| GO:0050921 | positive regulation of chemotaxis | 1.00E+00 | 9.62E-01 | 1.00E+00 | | 1.20E-02 | | 1.00E+00 | | 1.00E+00 | | 9.83E-01 | |  |
| GO:0043152 | induction of bacterial agglutination | 1.00E+00 | 9.84E-01 | 1.00E+00 | | 1.24E-02 | | 3.89E-01 | | 1.00E+00 | | 1.00E+00 | |  |
| GO:0033993 | response to lipid | 1.00E+00 | 9.84E-01 | 1.00E+00 | | 1.38E-02 | | 2.10E-02 | | 1.00E+00 | | 1.00E+00 | |  |
| GO:0071526 | semaphorin-plexin signaling pathway | 1.00E+00 | 9.84E-01 | 1.00E+00 | | 1.47E-02 | | 4.48E-02 | | 1.00E+00 | | 1.00E+00 | |  |
| GO:0050678 | regulation of epithelial cell proliferation | 1.00E+00 | 9.29E-01 | 1.00E+00 | | 1.50E-02 | | 4.01E-02 | | 1.00E+00 | | 1.00E+00 | |  |
| GO:0010466 | negative regulation of peptidase activity | 1.00E+00 | 9.39E-01 | 1.00E+00 | | 1.55E-02 | | 8.07E-01 | | 1.00E+00 | | 1.00E+00 | |  |
| GO:0010951 | negative regulation of endopeptidase activity | 1.00E+00 | 9.40E-01 | 1.00E+00 | | 1.56E-02 | | 9.94E-01 | | 1.00E+00 | | 1.00E+00 | |  |
| GO:0099560 | synaptic membrane adhesion | 1.00E+00 | 9.96E-01 | 1.00E+00 | | 1.58E-02 | | 2.80E-02 | | 1.00E+00 | | 1.00E+00 | |  |
| GO:0002043 | blood vessel endothelial cell proliferation involved in sprouting angiogenesis | 1.00E+00 | 9.39E-01 | 1.00E+00 | | 1.58E-02 | | 1.82E-01 | | 1.00E+00 | | 9.40E-01 | |  |
| GO:0031214 | biomineral tissue development | 1.00E+00 | 9.39E-01 | 1.00E+00 | | 1.58E-02 | | 1.83E-01 | | 1.00E+00 | | 1.00E+00 | |  |
| GO:0030516 | regulation of axon extension | 1.00E+00 | 9.90E-01 | 1.00E+00 | | 1.61E-02 | | 3.40E-02 | | 1.00E+00 | | 1.00E+00 | |  |
| GO:0007178 | transmembrane receptor protein serine/threonine kinase signaling pathway | 1.00E+00 | 9.68E-01 | 1.00E+00 | | 1.61E-02 | | 1.70E-01 | | 1.00E+00 | | 1.00E+00 | |  |
| GO:0008037 | cell recognition | 1.00E+00 | 9.94E-01 | 1.00E+00 | | 1.65E-02 | | 1.16E-03 | | 1.00E+00 | | 1.00E+00 | |  |
| GO:0043410 | positive regulation of MAPK cascade | 1.00E+00 | 9.98E-01 | 1.00E+00 | | 1.74E-02 | | 5.84E-01 | | 1.00E+00 | | 1.00E+00 | |  |
| GO:1990138 | neuron projection extension | 1.00E+00 | 9.84E-01 | 1.00E+00 | | 1.75E-02 | | 6.03E-02 | | 1.00E+00 | | 9.55E-01 | |  |
| GO:0003253 | cardiac neural crest cell migration involved in outflow tract morphogenesis | 1.00E+00 | 1.00E+00 | 1.00E+00 | | 1.75E-02 | | 1.38E-01 | | 1.00E+00 | | 1.00E+00 | |  |
| GO:0033564 | anterior/posterior axon guidance | 1.00E+00 | 1.00E+00 | 1.00E+00 | | 1.75E-02 | | 1.38E-01 | | 1.00E+00 | | 1.00E+00 | |  |
| GO:0003415 | chondrocyte hypertrophy | 1.00E+00 | 9.74E-01 | 1.00E+00 | | 1.75E-02 | | 7.86E-01 | | 1.00E+00 | | 1.00E+00 | |  |
| GO:0003002 | regionalization | 1.00E+00 | 9.62E-01 | 1.00E+00 | | 1.91E-02 | | 3.47E-02 | | 1.00E+00 | | 1.00E+00 | |  |
| GO:0090288 | negative regulation of cellular response to growth factor stimulus | 1.00E+00 | 9.29E-01 | 1.00E+00 | | 1.94E-02 | | 5.94E-01 | | 1.00E+00 | | 1.00E+00 | |  |
| GO:0019730 | antimicrobial humoral response | 1.00E+00 | 1.00E+00 | 1.00E+00 | | 1.95E-02 | | 2.86E-01 | | 1.00E+00 | | 1.00E+00 | |  |
| GO:1903034 | regulation of response to wounding | 1.00E+00 | 9.36E-01 | 1.00E+00 | | 2.01E-02 | | 1.59E-01 | | 1.00E+00 | | 1.00E+00 | |  |
| GO:1901888 | regulation of cell junction assembly | 1.00E+00 | 1.00E+00 | 1.00E+00 | | 2.04E-02 | | 1.47E-05 | | 1.00E+00 | | 1.00E+00 | |  |
| GO:0048675 | axon extension | 1.00E+00 | 9.91E-01 | 1.00E+00 | | 2.11E-02 | | 7.92E-02 | | 1.00E+00 | | 1.00E+00 | |  |
| GO:0048843 | negative regulation of axon extension involved in axon guidance | 1.00E+00 | 9.90E-01 | 1.00E+00 | | 2.24E-02 | | 7.18E-02 | | 1.00E+00 | | 1.00E+00 | |  |
| GO:0009991 | response to extracellular stimulus | 1.00E+00 | 9.29E-01 | 1.00E+00 | | 2.28E-02 | | 7.83E-01 | | 1.00E+00 | | 9.72E-01 | |  |
| GO:0090027 | negative regulation of monocyte chemotaxis | 1.00E+00 | 1.00E+00 | 1.00E+00 | | 2.46E-02 | | 4.44E-01 | | 1.00E+00 | | 1.00E+00 | |  |
| GO:1905310 | regulation of cardiac neural crest cell migration involved in outflow tract morphogenesis | 1.00E+00 | 1.00E+00 | 1.00E+00 | | 2.46E-02 | | 4.44E-01 | | 1.00E+00 | | 1.00E+00 | |  |
| GO:0044278 | cell wall disruption in another organism | 1.00E+00 | 1.00E+00 | 1.00E+00 | | 2.46E-02 | | 4.44E-01 | | 1.00E+00 | | 1.00E+00 | |  |
| GO:0048247 | lymphocyte chemotaxis | 1.00E+00 | 9.90E-01 | 1.00E+00 | | 2.50E-02 | | 5.84E-01 | | 1.00E+00 | | 1.00E+00 | |  |
| GO:0009953 | dorsal/ventral pattern formation | 1.00E+00 | 9.86E-01 | 1.00E+00 | | 2.61E-02 | | 6.68E-02 | | 1.00E+00 | | 9.93E-01 | |  |
| GO:0071674 | mononuclear cell migration | 1.00E+00 | 9.90E-01 | 1.00E+00 | | 2.68E-02 | | 3.63E-01 | | 1.00E+00 | | 1.00E+00 | |  |
| GO:0001503 | ossification | 1.00E+00 | 9.33E-01 | 1.00E+00 | | 2.76E-02 | | 2.42E-01 | | 1.00E+00 | | 1.00E+00 | |  |
| GO:0051346 | negative regulation of hydrolase activity | 1.00E+00 | 9.29E-01 | 1.00E+00 | | 2.78E-02 | | 1.00E+00 | | 1.00E+00 | | 1.00E+00 | |  |
| GO:0051965 | positive regulation of synapse assembly | 1.00E+00 | 9.99E-01 | 1.00E+00 | | 2.89E-02 | | 1.70E-04 | | 1.00E+00 | | 1.00E+00 | |  |
| GO:0034104 | negative regulation of tissue remodeling | 1.00E+00 | 9.98E-01 | 1.00E+00 | | 3.07E-02 | | 3.01E-01 | | 1.00E+00 | | 1.00E+00 | |  |
| GO:0010628 | positive regulation of gene expression | 1.00E+00 | 9.90E-01 | 1.00E+00 | | 3.39E-02 | | 2.47E-01 | | 1.00E+00 | | 1.00E+00 | |  |
| GO:0051093 | negative regulation of developmental process | 1.00E+00 | 9.55E-01 | 1.00E+00 | | 3.50E-02 | | 5.72E-03 | | 1.00E+00 | | 1.00E+00 | |  |
| GO:0040013 | negative regulation of locomotion | 1.00E+00 | 9.35E-01 | 1.00E+00 | | 3.63E-02 | | 4.58E-03 | | 1.00E+00 | | 9.93E-01 | |  |
| GO:0048640 | negative regulation of developmental growth | 1.00E+00 | 9.94E-01 | 1.00E+00 | | 3.78E-02 | | 6.34E-02 | | 1.00E+00 | | 1.00E+00 | |  |
| GO:0019221 | cytokine-mediated signaling pathway | 1.00E+00 | 1.00E+00 | 1.00E+00 | | 3.87E-02 | | 1.74E-02 | | 1.00E+00 | | 1.00E+00 | |  |
| GO:0045861 | negative regulation of proteolysis | 1.00E+00 | 9.29E-01 | 1.00E+00 | | 3.87E-02 | | 8.70E-01 | | 1.00E+00 | | 9.93E-01 | |  |
| GO:0043542 | endothelial cell migration | 1.00E+00 | 9.33E-01 | 1.00E+00 | | 3.88E-02 | | 3.62E-01 | | 1.00E+00 | | 9.12E-01 | |  |
| GO:0001974 | blood vessel remodeling | 1.00E+00 | 9.90E-01 | 1.00E+00 | | 4.07E-02 | | 3.73E-02 | | 1.00E+00 | | 1.00E+00 | |  |
| GO:0007631 | feeding behavior | 1.00E+00 | 9.94E-01 | 1.00E+00 | | 4.09E-02 | | 7.21E-02 | | 1.00E+00 | | 1.00E+00 | |  |
| GO:0030308 | negative regulation of cell growth | 1.00E+00 | 9.74E-01 | 1.00E+00 | | 4.09E-02 | | 4.13E-01 | | 1.00E+00 | | 1.00E+00 | |  |
| GO:1903556 | negative regulation of tumor necrosis factor superfamily cytokine production | 1.00E+00 | 9.62E-01 | 1.00E+00 | | 4.33E-02 | | 4.58E-01 | | 1.00E+00 | | 1.00E+00 | |  |
| GO:0031667 | response to nutrient levels | 1.00E+00 | 9.29E-01 | 1.00E+00 | | 4.33E-02 | | 7.86E-01 | | 1.00E+00 | | 9.82E-01 | |  |
| GO:0098542 | defense response to other organism | 1.00E+00 | 9.44E-01 | 1.00E+00 | | 4.36E-02 | | 1.91E-01 | | 1.00E+00 | | 1.00E+00 | |  |
| GO:0045743 | positive regulation of fibroblast growth factor receptor signaling pathway | 1.00E+00 | 9.80E-01 | 1.00E+00 | | 4.36E-02 | | 2.42E-01 | | 1.00E+00 | | 1.00E+00 | |  |
| GO:0050679 | positive regulation of epithelial cell proliferation | 1.00E+00 | 9.39E-01 | 1.00E+00 | | 4.36E-02 | | 2.50E-01 | | 1.00E+00 | | 1.00E+00 | |  |
| GO:1903975 | regulation of glial cell migration | 1.00E+00 | 9.80E-01 | 1.00E+00 | | 4.59E-02 | | 5.63E-01 | | 1.00E+00 | | 1.00E+00 | |  |
| GO:0048562 | embryonic organ morphogenesis | 1.00E+00 | 9.29E-01 | 1.00E+00 | | 4.60E-02 | | 2.08E-01 | | 1.00E+00 | | 1.00E+00 | |  |
| GO:0045785 | positive regulation of cell adhesion | 1.00E+00 | 9.79E-01 | 1.00E+00 | | 4.64E-02 | | 9.94E-02 | | 1.00E+00 | | 9.93E-01 | |  |
| GO:0007389 | pattern specification process | 1.00E+00 | 9.62E-01 | 1.00E+00 | | 4.79E-02 | | 4.29E-02 | | 1.00E+00 | | 1.00E+00 | |  |
| GO:0050829 | defense response to Gram-negative bacterium | 1.00E+00 | 9.39E-01 | 1.00E+00 | | 4.79E-02 | | 5.94E-01 | | 1.00E+00 | | 1.00E+00 | |  |
| GO:1903555 | regulation of tumor necrosis factor superfamily cytokine production | 1.00E+00 | 9.33E-01 | 1.00E+00 | | 4.80E-02 | | 1.00E+00 | | 1.00E+00 | | 1.00E+00 | |  |
| GO:0050680 | negative regulation of epithelial cell proliferation | 1.00E+00 | 9.33E-01 | 1.00E+00 | | 5.27E-02 | | 3.66E-02 | | 1.00E+00 | | 1.00E+00 | |  |
| GO:0021953 | central nervous system neuron differentiation | 1.00E+00 | 9.80E-01 | 1.00E+00 | | 5.43E-02 | | 4.01E-02 | | 1.00E+00 | | 1.00E+00 | |  |
| GO:0001667 | ameboidal-type cell migration | 1.00E+00 | 9.90E-01 | 1.00E+00 | | 5.84E-02 | | 3.90E-02 | | 1.00E+00 | | 9.42E-01 | |  |
| GO:0051963 | regulation of synapse assembly | 1.00E+00 | 9.98E-01 | 1.00E+00 | | 6.36E-02 | | 1.04E-05 | | 1.00E+00 | | 1.00E+00 | |  |
| GO:0050808 | synapse organization | 1.00E+00 | 9.99E-01 | 1.00E+00 | | 6.36E-02 | | 6.30E-04 | | 1.00E+00 | | 1.00E+00 | |  |
| GO:0007157 | heterophilic cell-cell adhesion via plasma membrane cell adhesion molecules | 1.00E+00 | 9.68E-01 | 1.00E+00 | | 6.36E-02 | | 6.68E-03 | | 1.00E+00 | | 1.00E+00 | |  |
| GO:0042742 | defense response to bacterium | 1.00E+00 | 9.86E-01 | 2.76E-01 | | 6.95E-02 | | 3.19E-02 | | 1.00E+00 | | 1.00E+00 | |  |
| GO:0045670 | regulation of osteoclast differentiation | 1.00E+00 | 9.84E-01 | 1.00E+00 | | 7.40E-02 | | 7.56E-03 | | 1.00E+00 | | 1.00E+00 | |  |
| GO:0021782 | glial cell development | 1.00E+00 | 9.91E-01 | 1.00E+00 | | 7.59E-02 | | 1.67E-03 | | 1.00E+00 | | 1.00E+00 | |  |
| GO:0090497 | mesenchymal cell migration | 1.00E+00 | 1.00E+00 | 1.00E+00 | | 8.57E-02 | | 5.18E-04 | | 1.00E+00 | | 1.00E+00 | |  |
| GO:0061138 | morphogenesis of a branching epithelium | 1.00E+00 | 9.90E-01 | 1.00E+00 | | 8.57E-02 | | 1.96E-02 | | 1.00E+00 | | 1.00E+00 | |  |
| GO:0021602 | cranial nerve morphogenesis | 1.00E+00 | 9.94E-01 | 1.00E+00 | | 9.06E-02 | | 6.68E-03 | | 1.00E+00 | | 1.00E+00 | |  |
| GO:0085029 | extracellular matrix assembly | 1.00E+00 | 1.00E+00 | 1.00E+00 | | 9.70E-02 | | 4.15E-03 | | 1.00E+00 | | 1.00E+00 | |  |
| GO:0002683 | negative regulation of immune system process | 1.00E+00 | 9.94E-01 | 1.00E+00 | | 9.70E-02 | | 2.75E-02 | | 1.00E+00 | | 1.00E+00 | |  |
| GO:0014002 | astrocyte development | 1.00E+00 | 9.90E-01 | 1.00E+00 | | 9.70E-02 | | 4.48E-02 | | 1.00E+00 | | 1.00E+00 | |  |
| GO:0007416 | synapse assembly | 1.00E+00 | 9.98E-01 | 1.00E+00 | | 1.18E-01 | | 2.64E-05 | | 1.00E+00 | | 1.00E+00 | |  |
| GO:0001763 | morphogenesis of a branching structure | 1.00E+00 | 9.93E-01 | 1.00E+00 | | 1.23E-01 | | 2.26E-02 | | 1.00E+00 | | 1.00E+00 | |  |
| GO:0001755 | neural crest cell migration | 1.00E+00 | 1.00E+00 | 1.00E+00 | | 1.37E-01 | | 8.55E-04 | | 1.00E+00 | | 1.00E+00 | |  |
| GO:0030316 | osteoclast differentiation | 1.00E+00 | 9.91E-01 | 1.00E+00 | | 1.37E-01 | | 1.71E-02 | | 1.00E+00 | | 1.00E+00 | |  |
| GO:0060312 | regulation of blood vessel remodeling | 1.00E+00 | 9.98E-01 | 1.00E+00 | | 1.53E-01 | | 3.76E-02 | | 1.00E+00 | | 1.00E+00 | |  |
| GO:0043010 | camera-type eye development | 1.00E+00 | 9.33E-01 | 1.00E+00 | | 1.68E-01 | | 2.86E-02 | | 1.00E+00 | | 1.00E+00 | |  |
| GO:0002250 | adaptive immune response | 1.00E+00 | 9.90E-01 | 1.59E-02 | | 1.86E-01 | | 1.39E-03 | | 1.00E+00 | | 1.00E+00 | |  |
| GO:0042552 | myelination | 1.00E+00 | 9.86E-01 | 1.00E+00 | | 1.86E-01 | | 1.54E-02 | | 1.00E+00 | | 1.00E+00 | |  |
| GO:0008366 | axon ensheathment | 1.00E+00 | 9.86E-01 | 1.00E+00 | | 1.86E-01 | | 1.54E-02 | | 1.00E+00 | | 1.00E+00 | |  |
| GO:0007272 | ensheathment of neurons | 1.00E+00 | 9.86E-01 | 1.00E+00 | | 1.86E-01 | | 1.54E-02 | | 1.00E+00 | | 1.00E+00 | |  |
| GO:0002460 | adaptive immune response based on somatic recombination of immune receptors built from immunoglobulin superfamily domains | 1.00E+00 | 9.94E-01 | 8.72E-02 | | 1.86E-01 | | 2.64E-02 | | 1.00E+00 | | 1.00E+00 | |  |
| GO:0014033 | neural crest cell differentiation | 1.00E+00 | 1.00E+00 | 1.00E+00 | | 1.95E-01 | | 1.11E-02 | | 1.00E+00 | | 1.00E+00 | |  |
| GO:1905606 | regulation of presynapse assembly | 1.00E+00 | 9.99E-01 | 1.00E+00 | | 2.12E-01 | | 1.71E-02 | | 1.00E+00 | | 1.00E+00 | |  |
| GO:0014032 | neural crest cell development | 1.00E+00 | 1.00E+00 | 1.00E+00 | | 2.18E-01 | | 1.01E-02 | | 1.00E+00 | | 1.00E+00 | |  |
| GO:0099174 | regulation of presynapse organization | 1.00E+00 | 9.99E-01 | 1.00E+00 | | 2.54E-01 | | 1.01E-02 | | 1.00E+00 | | 1.00E+00 | |  |
| GO:0050806 | positive regulation of synaptic transmission | 1.00E+00 | 1.00E+00 | 1.00E+00 | | 2.57E-01 | | 1.82E-02 | | 1.00E+00 | | 1.00E+00 | |  |
| GO:0042130 | negative regulation of T cell proliferation | 1.00E+00 | 1.00E+00 | 1.00E+00 | | 2.75E-01 | | 5.61E-03 | | 1.00E+00 | | 1.00E+00 | |  |
| GO:0007156 | homophilic cell adhesion via plasma membrane adhesion molecules | 1.00E+00 | 9.29E-01 | 1.00E+00 | | 2.80E-01 | | 3.84E-03 | | 1.00E+00 | | 1.00E+00 | |  |
| GO:0045671 | negative regulation of osteoclast differentiation | 1.00E+00 | 9.76E-01 | 1.00E+00 | | 2.80E-01 | | 2.93E-02 | | 1.00E+00 | | 1.00E+00 | |  |
| GO:0030336 | negative regulation of cell migration | 1.00E+00 | 9.29E-01 | 1.00E+00 | | 2.80E-01 | | 4.29E-02 | | 1.00E+00 | | 9.40E-01 | |  |
| GO:0071676 | negative regulation of mononuclear cell migration | 1.00E+00 | 1.00E+00 | 1.00E+00 | | 2.80E-01 | | 4.80E-02 | | 1.00E+00 | | 1.00E+00 | |  |
| GO:0048864 | stem cell development | 1.00E+00 | 1.00E+00 | 1.00E+00 | | 2.83E-01 | | 3.47E-02 | | 1.00E+00 | | 1.00E+00 | |  |
| GO:0050807 | regulation of synapse organization | 1.00E+00 | 9.99E-01 | 1.00E+00 | | 3.04E-01 | | 1.15E-03 | | 1.00E+00 | | 1.00E+00 | |  |
| GO:0099054 | presynapse assembly | 1.00E+00 | 1.00E+00 | 1.00E+00 | | 3.29E-01 | | 4.15E-03 | | 1.00E+00 | | 1.00E+00 | |  |
| GO:0050803 | regulation of synapse structure or activity | 1.00E+00 | 9.99E-01 | 1.00E+00 | | 3.58E-01 | | 7.74E-04 | | 1.00E+00 | | 1.00E+00 | |  |
| GO:0099172 | presynapse organization | 1.00E+00 | 9.98E-01 | 1.00E+00 | | 3.58E-01 | | 6.68E-03 | | 1.00E+00 | | 1.00E+00 | |  |
| GO:0050672 | negative regulation of lymphocyte proliferation | 1.00E+00 | 1.00E+00 | 1.00E+00 | | 3.80E-01 | | 3.67E-02 | | 1.00E+00 | | 1.00E+00 | |  |
| GO:0070664 | negative regulation of leukocyte proliferation | 1.00E+00 | 1.00E+00 | 1.00E+00 | | 3.98E-01 | | 1.54E-02 | | 1.00E+00 | | 1.00E+00 | |  |
| GO:0002252 | immune effector process | 1.00E+00 | 9.93E-01 | 1.59E-02 | | 3.98E-01 | | 9.82E-02 | | 1.00E+00 | | 1.00E+00 | |  |
| GO:0032945 | negative regulation of mononuclear cell proliferation | 1.00E+00 | 1.00E+00 | 1.00E+00 | | 3.98E-01 | | 2.64E-02 | | 1.00E+00 | | 1.00E+00 | |  |
| GO:0030002 | cellular anion homeostasis | 1.00E+00 | 9.90E-01 | 1.00E+00 | | 4.10E-01 | | 3.67E-02 | | 1.00E+00 | | 1.00E+00 | |  |
| GO:0055083 | monovalent inorganic anion homeostasis | 1.00E+00 | 9.90E-01 | 1.00E+00 | | 4.10E-01 | | 3.67E-02 | | 1.00E+00 | | 1.00E+00 | |  |
| GO:0002686 | negative regulation of leukocyte migration | 1.00E+00 | 9.90E-01 | 1.00E+00 | | 4.30E-01 | | 2.80E-02 | | 1.00E+00 | | 1.00E+00 | |  |
| GO:0009100 | glycoprotein metabolic process | 1.00E+00 | 9.68E-01 | 1.00E+00 | | 4.71E-01 | | 3.43E-02 | | 1.00E+00 | | 1.00E+00 | |  |
| GO:0002449 | lymphocyte mediated immunity | 1.00E+00 | 9.94E-01 | 1.59E-02 | | 4.71E-01 | | 5.38E-02 | | 1.00E+00 | | 1.00E+00 | |  |
| GO:0048754 | branching morphogenesis of an epithelial tube | 1.00E+00 | 9.84E-01 | 1.00E+00 | | 5.01E-01 | | 4.09E-02 | | 1.00E+00 | | 1.00E+00 | |  |
| GO:0021675 | nerve development | 1.00E+00 | 9.90E-01 | 1.00E+00 | | 5.58E-01 | | 5.96E-03 | | 1.00E+00 | | 1.00E+00 | |  |
| GO:0002695 | negative regulation of leukocyte activation | 1.00E+00 | 9.94E-01 | 1.00E+00 | | 5.67E-01 | | 3.27E-02 | | 1.00E+00 | | 1.00E+00 | |  |
| GO:0050868 | negative regulation of T cell activation | 1.00E+00 | 9.97E-01 | 1.00E+00 | | 5.85E-01 | | 4.08E-02 | | 1.00E+00 | | 1.00E+00 | |  |
| GO:0048521 | negative regulation of behavior | 1.00E+00 | 1.00E+00 | 1.00E+00 | | 5.92E-01 | | 4.82E-03 | | 1.00E+00 | | 1.00E+00 | |  |
| GO:0002855 | regulation of natural killer cell mediated immune response to tumor cell | 1.00E+00 | 9.94E-01 | 1.00E+00 | | 5.92E-01 | | 1.36E-02 | | 1.00E+00 | | 1.00E+00 | |  |
| GO:0002858 | regulation of natural killer cell mediated cytotoxicity directed against tumor cell target | 1.00E+00 | 9.94E-01 | 1.00E+00 | | 5.92E-01 | | 1.36E-02 | | 1.00E+00 | | 1.00E+00 | |  |
| GO:0002423 | natural killer cell mediated immune response to tumor cell | 1.00E+00 | 9.94E-01 | 1.00E+00 | | 5.92E-01 | | 1.36E-02 | | 1.00E+00 | | 1.00E+00 | |  |
| GO:0002420 | natural killer cell mediated cytotoxicity directed against tumor cell target | 1.00E+00 | 9.94E-01 | 1.00E+00 | | 5.92E-01 | | 1.36E-02 | | 1.00E+00 | | 1.00E+00 | |  |
| GO:2000252 | negative regulation of feeding behavior | 1.00E+00 | 1.00E+00 | 1.00E+00 | | 5.92E-01 | | 1.36E-02 | | 1.00E+00 | | 1.00E+00 | |  |
| GO:2000523 | regulation of T cell costimulation | 1.00E+00 | 9.90E-01 | 1.00E+00 | | 5.92E-01 | | 3.67E-02 | | 1.00E+00 | | 1.00E+00 | |  |
| GO:0060259 | regulation of feeding behavior | 1.00E+00 | 1.00E+00 | 1.00E+00 | | 5.92E-01 | | 4.10E-02 | | 1.00E+00 | | 1.00E+00 | |  |
| GO:0070593 | dendrite self-avoidance | 1.00E+00 | 1.00E+00 | 1.00E+00 | | 6.57E-01 | | 1.84E-02 | | 1.00E+00 | | 1.00E+00 | |  |
| GO:0002700 | regulation of production of molecular mediator of immune response | 1.00E+00 | 9.90E-01 | 6.61E-01 | | 6.68E-01 | | 4.84E-02 | | 1.00E+00 | | 1.00E+00 | |  |
| GO:1990834 | response to odorant | 1.00E+00 | 1.00E+00 | 1.00E+00 | | 7.43E-01 | | 3.37E-02 | | 1.00E+00 | | 1.00E+00 | |  |
| GO:0001957 | intramembranous ossification | 1.00E+00 | 1.00E+00 | 1.00E+00 | | 7.43E-01 | | 3.37E-02 | | 1.00E+00 | | 1.00E+00 | |  |
| GO:0036072 | direct ossification | 1.00E+00 | 1.00E+00 | 1.00E+00 | | 7.43E-01 | | 3.37E-02 | | 1.00E+00 | | 1.00E+00 | |  |
| GO:0006956 | complement activation | 1.00E+00 | 9.39E-01 | 1.01E-01 | | 7.79E-01 | | 1.00E+00 | | 4.86E-02 | | 1.00E+00 | |  |
| GO:0006958 | complement activation, classical pathway | 1.00E+00 | 9.85E-01 | 2.51E-02 | | 8.70E-01 | | 1.00E+00 | | 6.47E-02 | | 1.00E+00 | |  |
| GO:0002455 | humoral immune response mediated by circulating immunoglobulin | 1.00E+00 | 9.90E-01 | 1.59E-02 | | 8.88E-01 | | 1.00E+00 | | 1.08E-01 | | 1.00E+00 | |  |
| GO:0033045 | regulation of sister chromatid segregation | 1.00E+00 | 4.32E-02 | 1.00E+00 | | 1.00E+00 | | 1.00E+00 | | 1.97E-01 | | 9.93E-01 | |  |

### **Supplemental Table 6: B-H p-values of cellular component associations with protein subsets**

| ID | Name | Intercept.A | Intercept.B | Intercept.C | Intercept.D | Slope.A | Slope.B | Slope.C |
| --- | --- | --- | --- | --- | --- | --- | --- | --- |
| GO:0005759 | mitochondrial matrix | 5.62E-10 | 8.83E-01 | 1.00E+00 | 1.00E+00 | 1.00E+00 | 1.00E+00 | 1.51E-10 |
| GO:0015630 | microtubule cytoskeleton | 6.43E-10 | 7.53E-01 | 1.00E+00 | 1.00E+00 | 1.00E+00 | 1.00E+00 | 4.06E-11 |
| GO:1990904 | ribonucleoprotein complex | 6.43E-10 | 9.19E-01 | 1.00E+00 | 1.00E+00 | 1.00E+00 | 1.00E+00 | 8.90E-09 |
| GO:0099080 | supramolecular complex | 7.45E-10 | 9.19E-01 | 1.00E+00 | 1.00E+00 | 1.00E+00 | 1.00E+00 | 7.53E-09 |
| GO:0031967 | organelle envelope | 4.94E-09 | 9.38E-01 | 1.00E+00 | 1.00E+00 | 1.00E+00 | 1.00E+00 | 2.93E-09 |
| GO:0031975 | envelope | 4.94E-09 | 9.38E-01 | 1.00E+00 | 1.00E+00 | 1.00E+00 | 1.00E+00 | 2.93E-09 |
| GO:0043209 | myelin sheath | 6.17E-09 | 1.00E+00 | 1.00E+00 | 1.00E+00 | 1.00E+00 | 1.00E+00 | 7.53E-09 |
| GO:0140535 | intracellular protein-containing complex | 6.17E-09 | 8.83E-01 | 1.00E+00 | 1.00E+00 | 1.00E+00 | 9.82E-01 | 5.92E-07 |
| GO:1902494 | catalytic complex | 4.19E-08 | 3.88E-01 | 1.00E+00 | 1.00E+00 | 1.00E+00 | 5.58E-01 | 1.94E-06 |
| GO:0099513 | polymeric cytoskeletal fiber | 1.06E-07 | 9.19E-01 | 1.00E+00 | 1.00E+00 | 1.00E+00 | 1.00E+00 | 1.87E-06 |
| GO:0036464 | cytoplasmic ribonucleoprotein granule | 9.92E-07 | 9.19E-01 | 1.00E+00 | 1.00E+00 | 1.00E+00 | 1.00E+00 | 9.00E-06 |
| GO:0035770 | ribonucleoprotein granule | 1.59E-06 | 8.86E-01 | 1.00E+00 | 1.00E+00 | 1.00E+00 | 1.00E+00 | 4.66E-06 |
| GO:0005740 | mitochondrial envelope | 3.86E-06 | 9.19E-01 | 1.00E+00 | 1.00E+00 | 1.00E+00 | 1.00E+00 | 2.09E-05 |
| GO:0045202 | synapse | 3.88E-06 | 1.00E+00 | 1.00E+00 | 8.06E-01 | 1.00E+00 | 1.00E+00 | 7.53E-05 |
| GO:0030055 | cell-substrate junction | 1.21E-05 | 1.00E+00 | 1.00E+00 | 8.09E-01 | 1.00E+00 | 1.00E+00 | 3.83E-05 |
| GO:0005925 | focal adhesion | 1.21E-05 | 1.00E+00 | 1.00E+00 | 8.09E-01 | 1.00E+00 | 1.00E+00 | 3.83E-05 |
| GO:0098794 | postsynapse | 1.21E-05 | 1.00E+00 | 1.00E+00 | 1.00E+00 | 1.00E+00 | 1.00E+00 | 8.96E-05 |
| GO:0005815 | microtubule organizing center | 1.46E-05 | 7.53E-01 | 1.00E+00 | 1.00E+00 | 1.00E+00 | 1.00E+00 | 4.19E-06 |
| GO:0099512 | supramolecular fiber | 1.46E-05 | 1.00E+00 | 1.00E+00 | 9.74E-01 | 1.00E+00 | 1.00E+00 | 3.83E-05 |
| GO:0031966 | mitochondrial membrane | 1.93E-05 | 8.86E-01 | 1.00E+00 | 1.00E+00 | 1.00E+00 | 1.00E+00 | 5.90E-05 |
| GO:0099081 | supramolecular polymer | 2.09E-05 | 1.00E+00 | 1.00E+00 | 9.96E-01 | 1.00E+00 | 1.00E+00 | 5.01E-05 |
| GO:0048471 | perinuclear region of cytoplasm | 2.76E-05 | 1.00E+00 | 1.00E+00 | 8.87E-01 | 1.00E+00 | 1.00E+00 | 1.51E-04 |
| GO:0140513 | nuclear protein-containing complex | 7.11E-05 | 2.59E-01 | 1.00E+00 | 1.00E+00 | 1.00E+00 | 5.54E-01 | 8.96E-05 |
| GO:0010494 | cytoplasmic stress granule | 8.37E-05 | 9.19E-01 | 1.00E+00 | 1.00E+00 | 1.00E+00 | 1.00E+00 | 2.01E-04 |
| GO:0098978 | glutamatergic synapse | 1.31E-04 | 1.00E+00 | 1.00E+00 | 5.80E-01 | 1.00E+00 | 1.00E+00 | 2.07E-03 |
| GO:0005813 | centrosome | 1.35E-04 | 8.83E-01 | 1.00E+00 | 1.00E+00 | 1.00E+00 | 1.00E+00 | 5.01E-05 |
| GO:0005874 | microtubule | 1.80E-04 | 8.86E-01 | 1.00E+00 | 1.00E+00 | 1.00E+00 | 1.00E+00 | 1.17E-04 |
| GO:1990234 | transferase complex | 1.86E-04 | 7.53E-01 | 1.00E+00 | 1.00E+00 | 1.00E+00 | 5.54E-01 | 1.47E-02 |
| GO:0030496 | midbody | 2.70E-04 | 1.00E+00 | 1.00E+00 | 1.00E+00 | 1.00E+00 | 1.00E+00 | 9.81E-05 |
| GO:0009898 | cytoplasmic side of plasma membrane | 4.09E-04 | 1.00E+00 | 1.00E+00 | 1.00E+00 | 1.00E+00 | 1.00E+00 | 1.51E-04 |
| GO:0005777 | peroxisome | 4.09E-04 | 1.00E+00 | 1.00E+00 | 1.00E+00 | 1.00E+00 | 1.00E+00 | 2.01E-04 |
| GO:0042579 | microbody | 4.09E-04 | 1.00E+00 | 1.00E+00 | 1.00E+00 | 1.00E+00 | 1.00E+00 | 2.01E-04 |
| GO:0005884 | actin filament | 7.27E-04 | 1.00E+00 | 1.00E+00 | 1.00E+00 | 1.00E+00 | 1.00E+00 | 6.80E-03 |
| GO:0098562 | cytoplasmic side of membrane | 7.64E-04 | 1.00E+00 | 1.00E+00 | 1.00E+00 | 1.00E+00 | 1.00E+00 | 4.92E-04 |
| GO:0009295 | nucleoid | 9.06E-04 | 1.00E+00 | 1.00E+00 | 1.00E+00 | 1.00E+00 | 1.00E+00 | 3.32E-03 |
| GO:0042645 | mitochondrial nucleoid | 9.06E-04 | 1.00E+00 | 1.00E+00 | 1.00E+00 | 1.00E+00 | 1.00E+00 | 3.32E-03 |
| GO:0032838 | plasma membrane bounded cell projection cytoplasm | 1.13E-03 | 9.19E-01 | 1.00E+00 | 1.00E+00 | 1.00E+00 | 1.00E+00 | 7.63E-04 |
| GO:0019866 | organelle inner membrane | 1.36E-03 | 9.37E-01 | 1.00E+00 | 1.00E+00 | 1.00E+00 | 1.00E+00 | 4.56E-04 |
| GO:0005730 | nucleolus | 1.86E-03 | 9.30E-01 | 1.00E+00 | 1.00E+00 | 1.00E+00 | 1.00E+00 | 1.57E-02 |
| GO:0015629 | actin cytoskeleton | 1.86E-03 | 1.00E+00 | 1.00E+00 | 9.87E-01 | 1.00E+00 | 1.00E+00 | 9.63E-03 |
| GO:0030027 | lamellipodium | 1.90E-03 | 1.00E+00 | 1.00E+00 | 1.00E+00 | 1.00E+00 | 1.00E+00 | 6.09E-04 |
| GO:0005643 | nuclear pore | 2.26E-03 | 1.00E+00 | 1.00E+00 | 1.00E+00 | 1.00E+00 | 1.00E+00 | 9.42E-04 |
| GO:1990124 | messenger ribonucleoprotein complex | 2.73E-03 | 1.00E+00 | 1.00E+00 | 1.00E+00 | 1.00E+00 | 1.00E+00 | 4.77E-02 |
| GO:0070161 | anchoring junction | 2.83E-03 | 1.00E+00 | 1.00E+00 | 4.45E-01 | 1.00E+00 | 1.00E+00 | 5.08E-03 |
| GO:0005635 | nuclear envelope | 3.02E-03 | 9.85E-01 | 1.00E+00 | 1.00E+00 | 1.00E+00 | 1.00E+00 | 2.66E-04 |
| GO:0022627 | cytosolic small ribosomal subunit | 3.02E-03 | 1.00E+00 | 1.00E+00 | 1.00E+00 | 1.00E+00 | 1.00E+00 | 4.77E-02 |
| GO:0031252 | cell leading edge | 3.39E-03 | 9.19E-01 | 1.00E+00 | 1.00E+00 | 1.00E+00 | 1.00E+00 | 2.05E-04 |
| GO:0005743 | mitochondrial inner membrane | 3.39E-03 | 9.38E-01 | 1.00E+00 | 1.00E+00 | 1.00E+00 | 1.00E+00 | 3.07E-03 |
| GO:0031901 | early endosome membrane | 3.47E-03 | 1.00E+00 | 1.00E+00 | 1.00E+00 | 1.00E+00 | 1.00E+00 | 4.17E-02 |
| GO:0012506 | vesicle membrane | 3.47E-03 | 1.00E+00 | 1.00E+00 | 1.00E+00 | 1.00E+00 | 1.00E+00 | 6.88E-02 |
| GO:0031234 | extrinsic component of cytoplasmic side of plasma membrane | 3.47E-03 | 1.00E+00 | 1.00E+00 | 1.00E+00 | 1.00E+00 | 1.00E+00 | 9.08E-03 |
| GO:0099568 | cytoplasmic region | 3.52E-03 | 8.86E-01 | 1.00E+00 | 1.00E+00 | 1.00E+00 | 1.00E+00 | 7.82E-04 |
| GO:0043197 | dendritic spine | 3.62E-03 | 1.00E+00 | 1.00E+00 | 1.00E+00 | 1.00E+00 | 1.00E+00 | 2.49E-02 |
| GO:0032153 | cell division site | 5.55E-03 | 1.00E+00 | 1.00E+00 | 1.00E+00 | 1.00E+00 | 1.00E+00 | 9.08E-03 |
| GO:0032154 | cleavage furrow | 5.55E-03 | 1.00E+00 | 1.00E+00 | 1.00E+00 | 1.00E+00 | 1.00E+00 | 9.08E-03 |
| GO:0030659 | cytoplasmic vesicle membrane | 5.55E-03 | 1.00E+00 | 1.00E+00 | 1.00E+00 | 1.00E+00 | 1.00E+00 | 1.10E-01 |
| GO:0005769 | early endosome | 5.95E-03 | 9.19E-01 | 1.00E+00 | 1.00E+00 | 1.00E+00 | 1.00E+00 | 6.52E-02 |
| GO:0005819 | spindle | 6.28E-03 | 2.59E-01 | 1.00E+00 | 1.00E+00 | 1.00E+00 | 1.00E+00 | 6.92E-04 |
| GO:0044309 | neuron spine | 6.28E-03 | 9.98E-01 | 1.00E+00 | 1.00E+00 | 1.00E+00 | 1.00E+00 | 4.17E-02 |
| GO:0005776 | autophagosome | 6.73E-03 | 1.00E+00 | 1.00E+00 | 1.00E+00 | 1.00E+00 | 1.00E+00 | 1.64E-02 |
| GO:0015935 | small ribosomal subunit | 6.95E-03 | 1.00E+00 | 1.00E+00 | 1.00E+00 | 1.00E+00 | 1.00E+00 | 8.57E-02 |
| GO:0030118 | clathrin coat | 7.24E-03 | 1.00E+00 | 1.00E+00 | 1.00E+00 | 1.00E+00 | 1.00E+00 | 2.41E-02 |
| GO:0001726 | ruffle | 8.14E-03 | 8.83E-01 | 1.00E+00 | 1.00E+00 | 1.00E+00 | 1.00E+00 | 1.90E-02 |
| GO:0031984 | organelle subcompartment | 8.23E-03 | 1.00E+00 | 1.00E+00 | 1.00E+00 | 1.00E+00 | 1.00E+00 | 2.49E-02 |
| GO:0000932 | P-body | 8.23E-03 | 1.00E+00 | 1.00E+00 | 1.00E+00 | 1.00E+00 | 1.00E+00 | 3.79E-02 |
| GO:0042470 | melanosome | 9.52E-03 | 1.00E+00 | 1.00E+00 | 9.29E-01 | 1.00E+00 | 1.00E+00 | 4.72E-02 |
| GO:0048770 | pigment granule | 9.52E-03 | 1.00E+00 | 1.00E+00 | 9.29E-01 | 1.00E+00 | 1.00E+00 | 4.72E-02 |
| GO:0101031 | chaperone complex | 1.00E-02 | 9.19E-01 | 1.00E+00 | 1.00E+00 | 1.00E+00 | 1.00E+00 | 4.21E-02 |
| GO:0042175 | nuclear outer membrane-endoplasmic reticulum membrane network | 1.06E-02 | 1.00E+00 | 1.00E+00 | 1.00E+00 | 1.00E+00 | 1.00E+00 | 6.22E-02 |
| GO:0031970 | organelle envelope lumen | 1.10E-02 | 1.00E+00 | 1.00E+00 | 1.00E+00 | 1.00E+00 | 1.00E+00 | 1.20E-02 |
| GO:0010008 | endosome membrane | 1.23E-02 | 9.38E-01 | 1.00E+00 | 1.00E+00 | 1.00E+00 | 1.00E+00 | 9.40E-02 |
| GO:0005758 | mitochondrial intermembrane space | 1.25E-02 | 1.00E+00 | 1.00E+00 | 1.00E+00 | 1.00E+00 | 1.00E+00 | 1.02E-02 |
| GO:0036477 | somatodendritic compartment | 1.31E-02 | 1.00E+00 | 1.00E+00 | 1.98E-01 | 1.00E+00 | 1.00E+00 | 1.73E-01 |
| GO:0005938 | cell cortex | 1.41E-02 | 1.00E+00 | 1.00E+00 | 1.00E+00 | 1.00E+00 | 1.00E+00 | 3.93E-03 |
| GO:0090543 | Flemming body | 1.43E-02 | 1.00E+00 | 1.00E+00 | 1.00E+00 | 1.00E+00 | 1.00E+00 | 4.72E-02 |
| GO:0120111 | neuron projection cytoplasm | 1.53E-02 | 8.41E-01 | 1.00E+00 | 1.00E+00 | 1.00E+00 | 1.00E+00 | 6.80E-03 |
| GO:0061695 | transferase complex, transferring phosphorus-containing groups | 1.53E-02 | 9.38E-01 | 1.00E+00 | 1.00E+00 | 1.00E+00 | 1.00E+00 | 1.87E-01 |
| GO:0098827 | endoplasmic reticulum subcompartment | 1.61E-02 | 1.00E+00 | 1.00E+00 | 1.00E+00 | 1.00E+00 | 1.00E+00 | 8.57E-02 |
| GO:0097447 | dendritic tree | 1.61E-02 | 1.00E+00 | 1.00E+00 | 7.52E-01 | 1.00E+00 | 1.00E+00 | 1.85E-01 |
| GO:0030425 | dendrite | 1.61E-02 | 1.00E+00 | 1.00E+00 | 7.52E-01 | 1.00E+00 | 1.00E+00 | 1.85E-01 |
| GO:0048475 | coated membrane | 1.63E-02 | 1.00E+00 | 1.00E+00 | 1.00E+00 | 1.00E+00 | 1.00E+00 | 2.12E-02 |
| GO:0030117 | membrane coat | 1.63E-02 | 1.00E+00 | 1.00E+00 | 1.00E+00 | 1.00E+00 | 1.00E+00 | 2.12E-02 |
| GO:0044391 | ribosomal subunit | 1.63E-02 | 1.00E+00 | 1.00E+00 | 1.00E+00 | 1.00E+00 | 1.00E+00 | 2.88E-02 |
| GO:0016442 | RISC complex | 1.63E-02 | 1.00E+00 | 1.00E+00 | 1.00E+00 | 1.00E+00 | 1.00E+00 | 4.72E-02 |
| GO:0031332 | RNAi effector complex | 1.63E-02 | 1.00E+00 | 1.00E+00 | 1.00E+00 | 1.00E+00 | 1.00E+00 | 4.72E-02 |
| GO:0030125 | clathrin vesicle coat | 1.63E-02 | 1.00E+00 | 1.00E+00 | 1.00E+00 | 1.00E+00 | 1.00E+00 | 4.72E-02 |
| GO:0031074 | nucleocytoplasmic transport complex | 1.63E-02 | 1.00E+00 | 1.00E+00 | 1.00E+00 | 1.00E+00 | 1.00E+00 | 4.72E-02 |
| GO:0031527 | filopodium membrane | 1.63E-02 | 1.00E+00 | 1.00E+00 | 1.00E+00 | 1.00E+00 | 1.00E+00 | 4.77E-02 |
| GO:0031143 | pseudopodium | 1.63E-02 | 1.00E+00 | 1.00E+00 | 1.00E+00 | 1.00E+00 | 1.00E+00 | 4.77E-02 |
| GO:0031941 | filamentous actin | 1.63E-02 | 1.00E+00 | 1.00E+00 | 1.00E+00 | 1.00E+00 | 1.00E+00 | 4.77E-02 |
| GO:0005768 | endosome | 1.85E-02 | 8.86E-01 | 1.00E+00 | 1.00E+00 | 1.00E+00 | 1.00E+00 | 9.00E-02 |
| GO:0005844 | polysome | 1.90E-02 | 9.19E-01 | 1.00E+00 | 1.00E+00 | 1.00E+00 | 1.00E+00 | 5.42E-03 |
| GO:0005840 | ribosome | 1.90E-02 | 8.15E-01 | 1.00E+00 | 1.00E+00 | 1.00E+00 | 1.00E+00 | 1.02E-02 |
| GO:0022626 | cytosolic ribosome | 1.90E-02 | 9.98E-01 | 1.00E+00 | 1.00E+00 | 1.00E+00 | 1.00E+00 | 6.69E-02 |
| GO:0098793 | presynapse | 1.93E-02 | 1.00E+00 | 1.00E+00 | 6.84E-01 | 1.00E+00 | 1.00E+00 | 5.23E-02 |
| GO:0099522 | cytosolic region | 1.93E-02 | 1.00E+00 | 1.00E+00 | 1.00E+00 | 1.00E+00 | 1.00E+00 | 7.88E-02 |
| GO:0070382 | exocytic vesicle | 1.98E-02 | 1.00E+00 | 1.00E+00 | 1.00E+00 | 1.00E+00 | 1.00E+00 | 6.79E-02 |
| GO:0032587 | ruffle membrane | 2.03E-02 | 7.53E-01 | 1.00E+00 | 1.00E+00 | 1.00E+00 | 1.00E+00 | 3.73E-02 |
| GO:0031968 | organelle outer membrane | 2.15E-02 | 8.86E-01 | 1.00E+00 | 1.00E+00 | 1.00E+00 | 1.00E+00 | 5.91E-02 |
| GO:0019867 | outer membrane | 2.15E-02 | 8.86E-01 | 1.00E+00 | 1.00E+00 | 1.00E+00 | 1.00E+00 | 5.91E-02 |
| GO:0005789 | endoplasmic reticulum membrane | 2.43E-02 | 1.00E+00 | 1.00E+00 | 1.00E+00 | 1.00E+00 | 1.00E+00 | 1.16E-01 |
| GO:0099091 | postsynaptic specialization, intracellular component | 2.44E-02 | 1.00E+00 | 1.00E+00 | 1.00E+00 | 1.00E+00 | 1.00E+00 | 9.00E-02 |
| GO:0030018 | Z disc | 2.77E-02 | 1.00E+00 | 1.00E+00 | 1.00E+00 | 1.00E+00 | 1.00E+00 | 4.80E-02 |
| GO:0099572 | postsynaptic specialization | 3.09E-02 | 1.00E+00 | 1.00E+00 | 8.52E-01 | 1.00E+00 | 1.00E+00 | 5.79E-02 |
| GO:0000502 | proteasome complex | 3.13E-02 | 1.00E+00 | 1.00E+00 | 1.00E+00 | 1.00E+00 | 1.00E+00 | 3.72E-02 |
| GO:0098798 | mitochondrial protein-containing complex | 3.46E-02 | 9.19E-01 | 1.00E+00 | 1.00E+00 | 1.00E+00 | 1.00E+00 | 8.95E-02 |
| GO:0030175 | filopodium | 3.49E-02 | 1.00E+00 | 1.00E+00 | 5.80E-01 | 1.00E+00 | 1.00E+00 | 5.04E-02 |
| GO:0014069 | postsynaptic density | 3.49E-02 | 1.00E+00 | 1.00E+00 | 7.15E-01 | 1.00E+00 | 1.00E+00 | 5.59E-02 |
| GO:1902554 | serine/threonine protein kinase complex | 3.49E-02 | 8.94E-01 | 1.00E+00 | 1.00E+00 | 1.00E+00 | 1.00E+00 | 2.34E-01 |
| GO:1902911 | protein kinase complex | 3.54E-02 | 9.38E-01 | 1.00E+00 | 1.00E+00 | 1.00E+00 | 1.00E+00 | 2.48E-01 |
| GO:0140534 | endoplasmic reticulum protein-containing complex | 4.04E-02 | 1.00E+00 | 1.00E+00 | 1.00E+00 | 1.00E+00 | 1.00E+00 | 5.64E-03 |
| GO:0032839 | dendrite cytoplasm | 4.04E-02 | 8.89E-01 | 1.00E+00 | 1.00E+00 | 1.00E+00 | 1.00E+00 | 4.72E-02 |
| GO:0000922 | spindle pole | 4.23E-02 | 4.93E-01 | 1.00E+00 | 1.00E+00 | 1.00E+00 | 1.00E+00 | 4.72E-02 |
| GO:0005741 | mitochondrial outer membrane | 4.31E-02 | 9.19E-01 | 1.00E+00 | 1.00E+00 | 1.00E+00 | 1.00E+00 | 9.40E-02 |
| GO:0071141 | SMAD protein complex | 4.31E-02 | 1.00E+00 | 1.00E+00 | 1.00E+00 | 1.00E+00 | 1.00E+00 | 9.40E-02 |
| GO:0042564 | NLS-dependent protein nuclear import complex | 4.31E-02 | 1.00E+00 | 1.00E+00 | 1.00E+00 | 1.00E+00 | 1.00E+00 | 9.40E-02 |
| GO:0016281 | eukaryotic translation initiation factor 4F complex | 4.31E-02 | 1.00E+00 | 1.00E+00 | 1.00E+00 | 1.00E+00 | 1.00E+00 | 9.40E-02 |
| GO:0005681 | spliceosomal complex | 4.41E-02 | 8.86E-01 | 1.00E+00 | 1.00E+00 | 1.00E+00 | 1.00E+00 | 2.72E-01 |
| GO:0005782 | peroxisomal matrix | 4.89E-02 | 1.00E+00 | 1.00E+00 | 1.00E+00 | 1.00E+00 | 1.00E+00 | 2.12E-02 |
| GO:0031907 | microbody lumen | 4.89E-02 | 1.00E+00 | 1.00E+00 | 1.00E+00 | 1.00E+00 | 1.00E+00 | 2.12E-02 |
| GO:0071144 | heteromeric SMAD protein complex | 4.96E-02 | 1.00E+00 | 1.00E+00 | 1.00E+00 | 1.00E+00 | 1.00E+00 | 1.10E-01 |
| GO:1990391 | DNA repair complex | 4.96E-02 | 1.00E+00 | 1.00E+00 | 1.00E+00 | 1.00E+00 | 1.00E+00 | 1.10E-01 |
| GO:0030131 | clathrin adaptor complex | 4.96E-02 | 1.00E+00 | 1.00E+00 | 1.00E+00 | 1.00E+00 | 1.00E+00 | 1.10E-01 |
| GO:0030119 | AP-type membrane coat adaptor complex | 4.96E-02 | 1.00E+00 | 1.00E+00 | 1.00E+00 | 1.00E+00 | 1.00E+00 | 1.10E-01 |
| GO:0031227 | intrinsic component of endoplasmic reticulum membrane | 4.96E-02 | 1.00E+00 | 1.00E+00 | 1.00E+00 | 1.00E+00 | 1.00E+00 | 1.85E-01 |
| GO:0005774 | vacuolar membrane | 5.42E-02 | 9.38E-01 | 1.00E+00 | 1.00E+00 | 1.00E+00 | 1.00E+00 | 4.72E-02 |
| GO:0000421 | autophagosome membrane | 6.08E-02 | 1.00E+00 | 1.00E+00 | 1.00E+00 | 1.00E+00 | 1.00E+00 | 4.77E-02 |
| GO:0030424 | axon | 2.79E-01 | 1.00E+00 | 1.00E+00 | 2.76E-03 | 7.89E-02 | 1.00E+00 | 4.45E-01 |
| GO:0099503 | secretory vesicle | 3.12E-01 | 1.00E+00 | 1.00E+00 | 2.52E-02 | 1.00E+00 | 1.00E+00 | 5.57E-01 |
| GO:0030426 | growth cone | 3.27E-01 | 1.00E+00 | 1.00E+00 | 2.83E-02 | 1.00E+00 | 1.00E+00 | 2.41E-01 |
| GO:0030427 | site of polarized growth | 3.27E-01 | 1.00E+00 | 1.00E+00 | 2.83E-02 | 1.00E+00 | 1.00E+00 | 2.41E-01 |
| GO:0030141 | secretory granule | 6.00E-01 | 1.00E+00 | 1.00E+00 | 6.53E-03 | 2.92E-01 | 1.00E+00 | 7.52E-01 |
| GO:0000785 | chromatin | 6.69E-01 | 4.81E-02 | 1.00E+00 | 1.00E+00 | 1.00E+00 | 2.67E-01 | 1.87E-01 |
| GO:0044295 | axonal growth cone | 8.02E-01 | 1.00E+00 | 1.00E+00 | 3.40E-02 | 4.63E-01 | 1.00E+00 | 7.83E-01 |
| GO:0033116 | endoplasmic reticulum-Golgi intermediate compartment membrane | 9.45E-01 | 1.00E+00 | 1.00E+00 | 1.88E-02 | 5.61E-01 | 1.00E+00 | 9.31E-01 |
| GO:0000791 | euchromatin | 9.59E-01 | 3.83E-02 | 1.00E+00 | 1.00E+00 | 1.00E+00 | 1.00E+00 | 6.21E-01 |
| GO:0005775 | vacuolar lumen | 9.95E-01 | 1.00E+00 | 1.00E+00 | 6.84E-01 | 1.94E-02 | 1.00E+00 | 9.56E-01 |
| GO:0098590 | plasma membrane region | 9.95E-01 | 9.19E-01 | 1.00E+00 | 1.12E-01 | 2.19E-02 | 1.00E+00 | 9.90E-01 |
| GO:0098637 | protein complex involved in cell-matrix adhesion | 1.00E+00 | 1.00E+00 | 1.00E+00 | 2.64E-02 | 6.10E-01 | 1.00E+00 | 9.89E-01 |
| GO:0072562 | blood microparticle | 1.00E+00 | 7.53E-01 | 1.00E+00 | 1.00E+00 | 1.00E+00 | 5.01E-05 | 1.00E+00 |
| GO:0031226 | intrinsic component of plasma membrane | 1.00E+00 | 1.00E+00 | 1.01E-01 | 2.76E-15 | 8.59E-22 | 1.00E+00 | 1.00E+00 |
| GO:0009986 | cell surface | 1.00E+00 | 1.00E+00 | 1.55E-01 | 9.94E-19 | 4.87E-20 | 1.00E+00 | 1.00E+00 |
| GO:0005887 | integral component of plasma membrane | 1.00E+00 | 1.00E+00 | 1.04E-01 | 1.71E-12 | 5.43E-19 | 1.00E+00 | 1.00E+00 |
| GO:0030312 | external encapsulating structure | 1.00E+00 | 1.00E+00 | 1.00E+00 | 2.89E-25 | 3.57E-17 | 1.00E+00 | 1.00E+00 |
| GO:0031012 | extracellular matrix | 1.00E+00 | 1.00E+00 | 1.00E+00 | 2.89E-25 | 3.57E-17 | 1.00E+00 | 1.00E+00 |
| GO:0062023 | collagen-containing extracellular matrix | 1.00E+00 | 1.00E+00 | 1.00E+00 | 1.50E-21 | 2.59E-14 | 1.00E+00 | 1.00E+00 |
| GO:0009897 | external side of plasma membrane | 1.00E+00 | 1.00E+00 | 1.55E-01 | 9.46E-08 | 2.21E-11 | 1.00E+00 | 1.00E+00 |
| GO:0043235 | receptor complex | 1.00E+00 | 1.00E+00 | 1.00E+00 | 9.34E-07 | 1.44E-07 | 1.00E+00 | 1.00E+00 |
| GO:0031225 | anchored component of membrane | 1.00E+00 | 1.00E+00 | 1.00E+00 | 1.34E-04 | 1.82E-06 | 1.00E+00 | 1.00E+00 |
| GO:0005604 | basement membrane | 1.00E+00 | 1.00E+00 | 1.00E+00 | 8.71E-07 | 1.64E-05 | 1.00E+00 | 1.00E+00 |
| GO:0098552 | side of membrane | 1.00E+00 | 1.00E+00 | 1.00E+00 | 2.52E-04 | 3.62E-05 | 1.00E+00 | 1.00E+00 |
| GO:0043202 | lysosomal lumen | 1.00E+00 | 1.00E+00 | 1.00E+00 | 6.48E-01 | 3.07E-04 | 1.00E+00 | 1.00E+00 |
| GO:0099240 | intrinsic component of synaptic membrane | 1.00E+00 | 1.00E+00 | 1.00E+00 | 4.66E-04 | 5.37E-04 | 1.00E+00 | 1.00E+00 |
| GO:0005796 | Golgi lumen | 1.00E+00 | 9.19E-01 | 1.00E+00 | 7.58E-02 | 2.71E-03 | 1.00E+00 | 1.00E+00 |
| GO:0098936 | intrinsic component of postsynaptic membrane | 1.00E+00 | 1.00E+00 | 1.00E+00 | 1.09E-02 | 3.45E-03 | 1.00E+00 | 1.00E+00 |
| GO:0099055 | integral component of postsynaptic membrane | 1.00E+00 | 1.00E+00 | 1.00E+00 | 1.09E-02 | 3.47E-03 | 1.00E+00 | 1.00E+00 |
| GO:0097060 | synaptic membrane | 1.00E+00 | 1.00E+00 | 1.00E+00 | 1.31E-02 | 3.84E-03 | 1.00E+00 | 1.00E+00 |
| GO:0099699 | integral component of synaptic membrane | 1.00E+00 | 1.00E+00 | 1.00E+00 | 1.55E-03 | 4.45E-03 | 1.00E+00 | 1.00E+00 |
| GO:0099060 | integral component of postsynaptic specialization membrane | 1.00E+00 | 1.00E+00 | 1.00E+00 | 1.78E-01 | 5.72E-03 | 1.00E+00 | 1.00E+00 |
| GO:0099061 | integral component of postsynaptic density membrane | 1.00E+00 | 1.00E+00 | 1.00E+00 | 1.23E-01 | 9.36E-03 | 1.00E+00 | 1.00E+00 |
| GO:0098982 | GABA-ergic synapse | 1.00E+00 | 1.00E+00 | 8.15E-01 | 5.97E-01 | 1.02E-02 | 1.00E+00 | 1.00E+00 |
| GO:0005788 | endoplasmic reticulum lumen | 1.00E+00 | 1.00E+00 | 1.00E+00 | 5.65E-05 | 1.17E-02 | 1.00E+00 | 1.00E+00 |
| GO:0031228 | intrinsic component of Golgi membrane | 1.00E+00 | 1.00E+00 | 5.72E-01 | 9.49E-01 | 1.17E-02 | 1.00E+00 | 1.00E+00 |
| GO:0005581 | collagen trimer | 1.00E+00 | 1.00E+00 | 1.00E+00 | 2.10E-01 | 1.25E-02 | 1.00E+00 | 1.00E+00 |
| GO:0098839 | postsynaptic density membrane | 1.00E+00 | 1.00E+00 | 1.00E+00 | 1.23E-01 | 1.54E-02 | 1.00E+00 | 1.00E+00 |
| GO:0098948 | intrinsic component of postsynaptic specialization membrane | 1.00E+00 | 1.00E+00 | 1.00E+00 | 2.10E-01 | 1.54E-02 | 1.00E+00 | 1.00E+00 |
| GO:0045211 | postsynaptic membrane | 1.00E+00 | 1.00E+00 | 1.00E+00 | 2.53E-01 | 1.54E-02 | 1.00E+00 | 1.00E+00 |
| GO:0099634 | postsynaptic specialization membrane | 1.00E+00 | 1.00E+00 | 1.00E+00 | 2.13E-01 | 1.75E-02 | 1.00E+00 | 1.00E+00 |
| GO:0046658 | anchored component of plasma membrane | 1.00E+00 | 1.00E+00 | 1.00E+00 | 2.56E-02 | 2.19E-02 | 1.00E+00 | 1.00E+00 |
| GO:0098889 | intrinsic component of presynaptic membrane | 1.00E+00 | 1.00E+00 | 1.00E+00 | 2.56E-02 | 2.49E-02 | 1.00E+00 | 1.00E+00 |
| GO:0099146 | intrinsic component of postsynaptic density membrane | 1.00E+00 | 1.00E+00 | 1.00E+00 | 1.71E-01 | 2.49E-02 | 1.00E+00 | 1.00E+00 |
| GO:0044298 | cell body membrane | 1.00E+00 | 1.00E+00 | 1.00E+00 | 1.88E-02 | 2.54E-02 | 1.00E+00 | 1.00E+00 |
| GO:0030173 | integral component of Golgi membrane | 1.00E+00 | 1.00E+00 | 1.00E+00 | 8.03E-01 | 3.21E-02 | 1.00E+00 | 1.00E+00 |
| GO:0098644 | complex of collagen trimers | 1.00E+00 | 1.00E+00 | 1.00E+00 | 6.84E-01 | 3.91E-02 | 1.00E+00 | 1.00E+00 |
| GO:0031233 | intrinsic component of external side of plasma membrane | 1.00E+00 | 1.00E+00 | 1.00E+00 | 2.42E-01 | 3.97E-02 | 1.00E+00 | 1.00E+00 |
| GO:0060076 | excitatory synapse | 1.00E+00 | 1.00E+00 | 1.00E+00 | 5.73E-01 | 3.97E-02 | 1.00E+00 | 1.00E+00 |
| GO:0032997 | Fc receptor complex | 1.00E+00 | 1.00E+00 | 1.00E+00 | 8.14E-01 | 3.97E-02 | 1.00E+00 | 1.00E+00 |
| GO:0045178 | basal part of cell | 1.00E+00 | 9.19E-01 | 1.00E+00 | 9.74E-01 | 3.97E-02 | 1.00E+00 | 1.00E+00 |
| GO:0032809 | neuronal cell body membrane | 1.00E+00 | 1.00E+00 | 1.00E+00 | 4.95E-02 | 4.92E-02 | 1.00E+00 | 1.00E+00 |
| GO:0043083 | synaptic cleft | 1.00E+00 | 9.19E-01 | 1.00E+00 | 1.78E-01 | 4.92E-02 | 1.00E+00 | 1.00E+00 |
| GO:0042734 | presynaptic membrane | 1.00E+00 | 1.00E+00 | 1.00E+00 | 2.33E-02 | 1.39E-01 | 1.00E+00 | 1.00E+00 |
| GO:0070820 | tertiary granule | 1.00E+00 | 1.00E+00 | 1.00E+00 | 1.97E-02 | 2.28E-01 | 1.00E+00 | 1.00E+00 |
| GO:0043256 | laminin complex | 1.00E+00 | 1.00E+00 | 1.00E+00 | 1.31E-02 | 3.32E-01 | 1.00E+00 | 1.00E+00 |
| GO:0046696 | lipopolysaccharide receptor complex | 1.00E+00 | 1.00E+00 | 1.00E+00 | 1.82E-02 | 1.00E+00 | 1.00E+00 | 1.00E+00 |
| GO:0048787 | presynaptic active zone membrane | 1.00E+00 | 1.00E+00 | 1.00E+00 | 2.71E-02 | 1.00E+00 | 1.00E+00 | 1.00E+00 |

### **Supplemental Table 7: Baseline characteristics according to clusters based on baseline values**

| Characteristic | Overall, N = 382^*^ | 1, N = 149^*^ | 2, N = 110^*^ | 3, N = 123^*^ | p-value^†^ |
| --- | --- | --- | --- | --- | --- |
| **Demographics** |  |  |  |  |  |
| Age at baseline visit (years) | 64 (56, 72) | 70 (64, 77) | 64 (56, 71) | 57 (47, 64) | **<0.001** |
| Men | 278 (73%) | 111 (74%) | 81 (74%) | 86 (70%) | 0.7 |
| Ethnicity: Caucasian | 351 (93%) | 144 (98%) | 101 (93%) | 106 (86%) | **0.001** |
| **Features of HF** |  |  |  |  |  |
| Duration (years) | 4.2 (1.6, 9.5) | 3.6 (0.9, 7.9) | 5.1 (2.2, 11.2) | 4.1 (1.4, 10.0) | **0.032** |
| NYHA class I or II | 276 (73%) | 104 (70%) | 77 (70%) | 95 (79%) | 0.2 |
| Systolic ejection fraction (%) | 30 (23, 36) | 31 (25, 39) | 26 (20, 31) | 28 (22, 32) | **0.003** |
| **Clinical characteristics** |  |  |  |  |  |
| BMI (kg/m2) | 26.5 (24.0, 30.1) | 26.3 (24.4, 29.2) | 25.7 (23.6, 30.4) | 27.4 (24.0, 30.7) | 0.4 |
| eGFR CKD-EPI (mL/min/1.73m2) | 58 (42, 77) | 53 (38, 73) | 59 (44, 78) | 72 (57, 86) | **0.001** |
| Systolic blood pressure (mmHg) | 114 (100, 130) | 122 (110, 138) | 110 (100, 128) | 110 (92, 120) | **<0.001** |
| Diastolic blood pressure (mmHg) | 70 (60, 78) | 72 (64, 80) | 70 (60, 77) | 70 (60, 75) | **0.003** |
| **Biomarker level** |  |  |  |  |  |
| Nt-proBNP (pmol/L) | 133 (46, 274) | 141 (57, 282) | 194 (70, 338) | 75 (20, 182) | **0.005** |
| Hs-Troponin T (ng/L) | 18 (9, 33) | 19 (11, 37) | 18 (10, 32) | 11 (6, 23) | **0.004** |
| CRP (mg/L) | 2.2 (0.9, 4.9) | 1.6 (0.8, 3.5) | 2.9 (1.1, 6.7) | 3.1 (1.4, 5.4) | **0.008** |
| **Etiology of HF** |  |  |  |  |  |
| Ischemic heart disease | 166 (43%) | 76 (51%) | 48 (44%) | 42 (34%) | **0.020** |
| Hypertension | 33 (8.6%) | 26 (17%) | 6 (5.5%) | 1 (0.8%) | **<0.001** |
| Secondary to valvular heart disease | 12 (3.1%) | 4 (2.7%) | 5 (4.5%) | 3 (2.4%) | 0.6 |
| Cardiomyopathy | 122 (32%) | 25 (17%) | 39 (35%) | 58 (47%) | **<0.001** |
| Hypertrophic (HCM) | 15 (3.9%) | 4 (2.7%) | 6 (5.5%) | 5 (4.1%) | 0.5 |
| Dilated (DCM) | 97 (25%) | 19 (13%) | 27 (25%) | 51 (41%) | **<0.001** |
| Restrictive | 0 (0%) | 0 (0%) | 0 (0%) | 0 (0%) | >0.9 |
| Arrhytmogenic right ventricular (ARVC) | 1 (0.3%) | 0 (0%) | 1 (0.9%) | 0 (0%) | 0.3 |
| Non compaction cardiomyopathy | 4 (1.0%) | 0 (0%) | 2 (1.8%) | 2 (1.6%) | 0.2 |
| Unclassified | 7 (1.8%) | 2 (1.3%) | 3 (2.7%) | 2 (1.6%) | 0.7 |
| Unknown | 27 (7.1%) | 14 (9.4%) | 6 (5.5%) | 7 (5.7%) | 0.4 |
| **Medical history** |  |  |  |  |  |
| Myocardial Infarction | 145 (38%) | 60 (41%) | 42 (39%) | 43 (35%) | 0.6 |
| PCI | 126 (33%) | 52 (35%) | 43 (39%) | 31 (25%) | 0.065 |
| CABG | 54 (14%) | 27 (18%) | 16 (15%) | 11 (8.9%) | 0.10 |
| atrial fibrillation | 137 (36%) | 64 (44%) | 47 (43%) | 26 (21%) | **<0.001** |
| other arrhythmia | 151 (40%) | 38 (26%) | 54 (50%) | 59 (48%) | **<0.001** |
| pacemaker implantation | 85 (23%) | 21 (15%) | 28 (26%) | 36 (29%) | **0.021** |
| ICD implantation | 254 (66%) | 83 (56%) | 79 (72%) | 92 (75%) | **0.002** |
| CRT | 113 (30%) | 43 (29%) | 28 (25%) | 42 (34%) | 0.3 |
| stroke (CVA/TIA) | 48 (13%) | 24 (16%) | 12 (11%) | 12 (9.8%) | 0.3 |
| chronic renal failure | 181 (48%) | 79 (54%) | 58 (53%) | 44 (36%) | **0.006** |
| Diabetes Mellitus | 98 (26%) | 41 (28%) | 31 (28%) | 26 (21%) | 0.4 |
| Known hypercholesterolemia | 160 (43%) | 56 (38%) | 47 (44%) | 57 (48%) | 0.3 |
| Hypertension | 166 (44%) | 65 (45%) | 48 (44%) | 53 (43%) | >0.9 |
| **Intoxication** |  |  |  |  |  |
| Smoking: Ever | 271 (71%) | 117 (79%) | 69 (63%) | 85 (70%) | **0.025** |
| Smoking: Current | 343 (90%) | 136 (91%) | 98 (90%) | 109 (89%) | 0.9 |
| **Medication** |  |  |  |  |  |
| Ace Inhibitor | 258 (68%) | 93 (63%) | 78 (71%) | 87 (71%) | 0.3 |
| Angiotensin II receptor blockers | 107 (28%) | 49 (33%) | 30 (27%) | 28 (23%) | 0.2 |
| Aldosteron antagonists | 293 (77%) | 106 (71%) | 84 (76%) | 103 (84%) | **0.050** |
| Diuretics other | 5 (1.3%) | 1 (0.7%) | 2 (1.8%) | 2 (1.6%) | 0.6 |
| Beta blockers | 350 (92%) | 134 (91%) | 99 (90%) | 117 (95%) | 0.3 |
| Aspirin | 77 (20%) | 31 (21%) | 25 (23%) | 21 (17%) | 0.5 |
| ^*^Median (IQR); n (%) | | | | | |
| ^†^Kruskal-Wallis rank sum test; Pearson's Chi-squared test; Fisher's exact test  BMI= Body mass index, PCI= Percutaneous coronary intervention, CABG= Coronary artery bypass surgery, ICD= implantable cardioverter-defibrillator, CRT= Cardiac resynchronisation therapy | | | | | |

### **Supplemental Table 8: Survival analysis using clusters based on the baseline values**

|  | Univariate | | Adjusted for Age, Sex and eGFR (CKD-EPI) | | Adjusted for relevant  Clinical Variables* | | Adjusted for relevant Clinical Variables* and NT-proBNP | |
| --- | --- | --- | --- | --- | --- | --- | --- | --- |
| **Characteristic** | **HR (95% CI)**^1^ | **p-value** | **HR (95% CI)**^1^ | **p-value** | **HR (95% CI)**^1^ | **p-value** | **HR (95% CI)**^1^ | **p-value** |
| subphenotype |  |  |  |  |  |  |  |  |
| 1 | — |  | — |  | — |  | — |  |
| 2 | 1.41 (0.89-2.23) | 0.15 | 1.69 (1.05-2.71) | **0.031** | 1.48 (0.88-2.48) | 0.14 | 1.51 (0.89-2.57) | 0.13 |
| 3 | 1.52 (0.98-2.38) | 0.064 | 2.11 (1.25-3.55) | **0.005** | 1.75 (1.00-3.06) | 0.050 | 2.42 (1.36-4.32) | **0.003** |
| R² | 0.010 |  | 0.056 |  | 0.175 |  | 0.277 |  |
| AIC | 1,280 |  | 1,121 |  | 1,012 |  | 972 |  |
| BIC | 1,285 |  | 1,135 |  | 1,040 |  | 1,003 |  |
| c-index^†^ | 0.533 |  | 0.598 |  | 0.680 |  | 0.754 |  |
| HR = Hazard Ratio, CI=Confidence Interval, AIC= Akaike information criterion, BIC= Bayesian information criterion  * Age, Gender, eGFR (CKD-EPI), Systolic Blood Pressure, Duration of HF, NYHA class, history of atrial fibrillation, other arrhythmia and chronic renal failure  † Harrell’s bias correction | | | | | | | | |

### **Supplemental Table 9: Baseline characteristics according to clusters based on second measurements**

| **Characteristic** | **Overall**, N = 357^*^ | **1**, N = 106^*^ | **2**, N = 116^*^ | **3**, N = 135^*^ | **p-value**^†^ |
| --- | --- | --- | --- | --- | --- |
| **Demographics** |  |  |  |  |  |
| Age at baseline visit (years) | 64 (56, 72) | 70 (62, 78) | 64 (56, 72) | 60 (50, 68) | **<0.001** |
| Men | 278 (73%) | 82 (73%) | 86 (69%) | 110 (75%) | 0.5 |
| Ethnicity: Caucasian | 351 (93%) | 107 (97%) | 114 (92%) | 130 (90%) | 0.066 |
| **Features of HF** |  |  |  |  |  |
| Duration (years) | 4.2 (1.6, 9.5) | 3.6 (0.8, 7.9) | 5.4 (2.2, 10.1) | 4.2 (1.6, 9.4) | **0.040** |
| NYHA class I or II | 276 (73%) | 80 (71%) | 84 (68%) | 112 (78%) | 0.2 |
| Systolic ejection fraction (%) | 30 (23, 36) | 30 (23, 37) | 29 (23, 38) | 27 (22, 32) | 0.079 |
| **Clinical characteristics** |  |  |  |  |  |
| BMI (kg/m2) | 26.5 (24.0, 30.1) | 25.8 (23.8, 28.3) | 27.8 (24.6, 30.7) | 26.6 (23.7, 30.4) | **0.011** |
| eGFR CKD-EPI (mL/min/1.73m2) | 58 (42, 77) | 56 (41, 73) | 57 (41, 76) | 67 (48, 85) | **0.048** |
| Systolic blood pressure (mmHg) | 114 (100, 130) | 120 (106, 136) | 120 (102, 130) | 110 (97, 120) | **<0.001** |
| Diastolic blood pressure (mmHg) | 70 (60, 78) | 74 (62, 80) | 70 (60, 77) | 70 (60, 75) | **0.015** |
| **Biomarker level** |  |  |  |  |  |
| Nt-proBNP (pmol/L) | 133 (46, 274) | 141 (70, 289) | 153 (39, 312) | 112 (26, 206) | 0.12 |
| Hs-Troponin T (ng/L) | 18 (9, 33) | 18 (10, 31) | 20 (10, 41) | 14 (8, 30) | 0.13 |
| CRP (mg/L) | 2.2 (0.9, 4.9) | 2.0 (0.8, 3.8) | 2.3 (1.0, 5.2) | 2.7 (1.3, 5.2) | 0.3 |
| **Etiology of HF** |  |  |  |  |  |
| Ischemic heart disease | 166 (43%) | 51 (46%) | 50 (40%) | 65 (45%) | 0.7 |
| Hypertension | 33 (8.6%) | 19 (17%) | 11 (8.9%) | 3 (2.1%) | **<0.001** |
| Secondary to valvular heart disease | 12 (3.1%) | 4 (3.6%) | 5 (4.0%) | 3 (2.1%) | 0.6 |
| Cardiomyopathy | 122 (32%) | 26 (23%) | 43 (35%) | 53 (36%) | 0.060 |
| Hypertrophic (HCM) | 15 (3.9%) | 4 (3.6%) | 7 (5.6%) | 4 (2.7%) | 0.5 |
| Dilated (DCM) | 97 (25%) | 21 (19%) | 33 (27%) | 43 (29%) | 0.14 |
| Restrictive | 0 (0%) | 0 (0%) | 0 (0%) | 0 (0%) | >0.9 |
| Arrhytmogenic right ventricular (ARVC) | 1 (0.3%) | 0 (0%) | 1 (0.8%) | 0 (0%) | 0.6 |
| Non compaction cardiomyopathy | 4 (1.0%) | 0 (0%) | 0 (0%) | 4 (2.7%) | **0.039** |
| Unclassified | 7 (1.8%) | 1 (0.9%) | 2 (1.6%) | 4 (2.7%) | 0.6 |
| Unknown | 27 (7.1%) | 13 (12%) | 5 (4.0%) | 9 (6.2%) | 0.066 |
| **Medical history** |  |  |  |  |  |
| Myocardial Infarction | 145 (38%) | 40 (37%) | 45 (37%) | 60 (41%) | 0.7 |
| PCI | 126 (33%) | 33 (29%) | 36 (29%) | 57 (39%) | 0.14 |
| CABG | 54 (14%) | 21 (19%) | 16 (13%) | 17 (12%) | 0.2 |
| atrial fibrillation | 137 (36%) | 45 (41%) | 49 (40%) | 43 (30%) | 0.12 |
| other arrhythmia | 151 (40%) | 30 (27%) | 48 (39%) | 73 (50%) | **0.001** |
| pacemaker implantation | 85 (23%) | 13 (13%) | 29 (24%) | 43 (30%) | **0.006** |
| ICD implantation | 254 (66%) | 66 (59%) | 82 (66%) | 106 (73%) | 0.070 |
| CRT | 113 (30%) | 37 (33%) | 29 (24%) | 47 (32%) | 0.2 |
| stroke (CVA/TIA) | 48 (13%) | 18 (16%) | 15 (12%) | 15 (10%) | 0.4 |
| chronic renal failure | 181 (48%) | 58 (53%) | 68 (55%) | 55 (38%) | **0.009** |
| Diabetes Mellitus | 98 (26%) | 24 (21%) | 43 (35%) | 31 (21%) | **0.020** |
| Known hypercholesterolemia | 160 (43%) | 38 (35%) | 54 (45%) | 68 (48%) | 0.094 |
| Hypertension | 166 (44%) | 43 (39%) | 54 (44%) | 69 (48%) | 0.4 |
| **Intoxication** |  |  |  |  |  |
| Smoking: Ever | 271 (71%) | 83 (74%) | 84 (68%) | 104 (72%) | 0.5 |
| Smoking: Current | 343 (90%) | 104 (93%) | 111 (90%) | 128 (89%) | 0.5 |
| **Medication** |  |  |  |  |  |
| Ace Inhibitor | 258 (68%) | 74 (67%) | 86 (69%) | 98 (67%) | 0.9 |
| Angiotensin II receptor blockers | 107 (28%) | 31 (28%) | 36 (29%) | 40 (27%) | >0.9 |
| Aldosteron antagonists | 293 (77%) | 88 (79%) | 90 (73%) | 115 (79%) | 0.4 |
| Diuretics other | 5 (1.3%) | 0 (0%) | 2 (1.6%) | 3 (2.1%) | 0.4 |
| Beta blockers | 350 (92%) | 99 (89%) | 112 (90%) | 139 (95%) | 0.2 |
| Aspirin | 77 (20%) | 20 (18%) | 20 (16%) | 37 (25%) | 0.14 |
| ^*^Median (IQR); n (%) | | | | | |
| ^†^Kruskal-Wallis rank sum test; Pearson's Chi-squared test; Fisher's exact test  BMI= Body mass index, PCI= Percutaneous coronary intervention, CABG= Coronary artery bypass surgery, ICD= implantable cardioverter-defibrillator, CRT= Cardiac resynchronisation therapy | | | | | |

### **Supplemental Table 10: Survival analysis using clusters based on second measurements**

|  | | Univariate | | Adjusted for Age, Sex and eGFR (CKD-EPI) | | | Adjusted for relevant Clinical Variables* | | | Adjusted for relevant Clinical Variables* and NT-proBNP | | |  |  |
| --- | --- | --- | --- | --- | --- | --- | --- | --- | --- | --- | --- | --- | --- | --- |
| **Characteristic** | **HR (95% CI)** | | **p-value** | | **HR (95% CI)** | **p-value** | | **HR (95% CI)** | **p-value** | | **HR (95% CI)** | **p-value** | |  |
| subphenotype |  | |  | |  |  | |  |  | |  |  | |  |
| 1 | — | |  | | — |  | | — |  | | — |  | |  |
| 2 | 1.86 (1.16-3.00) | | **0.011** | | 2.24 (1.36-3.71) | **0.002** | | 2.07 (1.22-3.52) | **0.007** | | 2.24 (1.29-3.88) | **0.004** | |  |
| 3 | 1.27 (0.78-2.09) | | 0.3 | | 1.56 (0.90-2.70) | 0.11 | | 1.43 (0.81-2.54) | 0.2 | | 2.21 (1.24-3.96) | **0.007** | |  |
| R² | 0.019 | |  | | 0.061 |  | | 0.184 |  | | 0.310 |  | |  |
| AIC | 1,276 | |  | | 1,120 |  | | 1,008 |  | | 957 |  | |  |
| BIC | 1,282 | |  | | 1,133 |  | | 1,037 |  | | 988 |  | |  |
| c-index | 0.557 | |  | | 0.592 |  | | 0.687 |  | | 0.768 |  | |  |
| HR = Hazard Ratio, CI=Confidence Interval, AIC= Akaike information criterion, BIC= Bayesian information criterion  * Age, Gender, eGFR (CKD-EPI), Systolic Blood Pressure, Duration of HF, NYHA class, history of atrial fibrillation, other arrhythmia and chronic renal failure  † Harrell’s bias correction | | | | | | | | | | | | | | |

### **Supplemental Table 11: Baseline characteristics according to clusters based the last measurements before PEP or censoring**

| **Characteristic** | **Overall**, N = 382^*^ | **1**, N = 118^*^ | **2**, N = 100^v^ | **3**, N = 164^*^ | **p-value**^†^ |
| --- | --- | --- | --- | --- | --- |
| **Demographics** |  |  |  |  |  |
| Age at baseline visit (years) | 64 (56, 72) | 69 (63, 74) | 68 (58, 79) | 58 (49, 66) | **<0.001** |
| Men | 278 (73%) | 83 (70%) | 74 (74%) | 121 (74%) | 0.8 |
| Ethnicity: Caucasian | 351 (93%) | 114 (97%) | 91 (92%) | 146 (90%) | **0.044** |
| **Features of HF** |  |  |  |  |  |
| Duration (years) | 4.2 (1.6, 9.5) | 3.1 (0.8, 6.8) | 5.4 (2.7, 10.3) | 4.1 (1.6, 10.7) | **0.004** |
| NYHA class I or II | 276 (73%) | 85 (72%) | 61 (61%) | 130 (80%) | **0.003** |
| Systolic ejection fraction (%) | 30 (23, 36) | 30 (25, 37) | 25 (19, 34) | 28 (22, 36) | **0.033** |
| **Clinical characteristics** |  |  |  |  |  |
| BMI (kg/m2) | 26.5 (24.0, 30.1) | 26.2 (24.5, 29.3) | 25.4 (23.3, 28.7) | 27.4 (24.2, 30.9) | **0.036** |
| eGFR CKD-EPI (mL/min/1.73m2) | 58 (42, 77) | 57 (46, 74) | 49 (37, 71) | 69 (49, 85) | **0.004** |
| Systolic blood pressure (mmHg) | 114 (100, 130) | 122 (108, 134) | 112 (100, 128) | 110 (96, 120) | **<0.001** |
| Diastolic blood pressure (mmHg) | 70 (60, 78) | 74 (66, 80) | 68 (60, 75) | 70 (60, 77) | **<0.001** |
| **Biomarker level** |  |  |  |  |  |
| Nt-proBNP (pmol/L) | 133 (46, 274) | 97 (46, 204) | 237 (126, 417) | 112 (24, 204) | **<0.001** |
| Hs-Troponin T (ng/L) | 18 (9, 33) | 17 (10, 27) | 27 (14, 44) | 13 (8, 24) | **<0.001** |
| CRP (mg/L) | 2.2 (0.9, 4.9) | 2.0 (0.8, 3.5) | 2.8 (1.0, 6.5) | 2.5 (1.2, 5.1) | 0.2 |
| **Etiology of HF** |  |  |  |  |  |
| Ischemic heart disease | 166 (43%) | 56 (47%) | 43 (43%) | 67 (41%) | 0.5 |
| Hypertension | 33 (8.6%) | 18 (15%) | 12 (12%) | 3 (1.8%) | **<0.001** |
| Secondary to valvular heart disease | 12 (3.1%) | 4 (3.4%) | 5 (5.0%) | 3 (1.8%) | 0.3 |
| Cardiomyopathy | 122 (32%) | 26 (22%) | 31 (31%) | 65 (40%) | **0.007** |
| Hypertrophic (HCM) | 15 (3.9%) | 5 (4.2%) | 5 (5.0%) | 5 (3.0%) | 0.7 |
| Dilated (DCM) | 97 (25%) | 20 (17%) | 22 (22%) | 55 (34%) | **0.005** |
| Restrictive | 0 (0%) | 0 (0%) | 0 (0%) | 0 (0%) | >0.9 |
| Arrhytmogenic right ventricular (ARVC) | 1 (0.3%) | 0 (0%) | 1 (1.0%) | 0 (0%) | 0.3 |
| Non compaction cardiomyopathy | 4 (1.0%) | 0 (0%) | 2 (2.0%) | 2 (1.2%) | 0.3 |
| Unclassified | 7 (1.8%) | 1 (0.8%) | 2 (2.0%) | 4 (2.4%) | 0.6 |
| Unknown | 27 (7.1%) | 11 (9.3%) | 6 (6.0%) | 10 (6.1%) | 0.5 |
| **Medical history** |  |  |  |  |  |
| Myocardial Infarction | 145 (38%) | 45 (39%) | 35 (35%) | 65 (40%) | 0.8 |
| PCI | 126 (33%) | 35 (30%) | 29 (29%) | 62 (38%) | 0.2 |
| CABG | 54 (14%) | 21 (18%) | 17 (17%) | 16 (9.8%) | 0.10 |
| Atrial fibrillation | 137 (36%) | 44 (38%) | 49 (50%) | 44 (27%) | **<0.001** |
| Other arrhythmia | 151 (40%) | 27 (23%) | 41 (41%) | 83 (51%) | **<0.001** |
| Pacemaker implantation | 85 (23%) | 14 (13%) | 23 (24%) | 48 (29%) | **0.005** |
| ICD implantation | 254 (66%) | 72 (61%) | 63 (63%) | 119 (73%) | 0.089 |
| CRT | 113 (30%) | 39 (33%) | 29 (29%) | 45 (28%) | 0.6 |
| Stroke (CVA/TIA) | 48 (13%) | 22 (19%) | 13 (13%) | 13 (7.9%) | **0.023** |
| Chronic renal failure | 181 (48%) | 55 (47%) | 64 (64%) | 62 (38%) | **<0.001** |
| Diabetes Mellitus | 98 (26%) | 27 (23%) | 37 (37%) | 34 (21%) | **0.010** |
| Known hypercholesterolemia | 160 (43%) | 41 (36%) | 37 (38%) | 82 (51%) | **0.018** |
| Hypertension | 166 (44%) | 46 (40%) | 46 (46%) | 74 (45%) | 0.6 |
| **Intoxication** |  |  |  |  |  |
| Smoking: Ever | 271 (71%) | 86 (73%) | 69 (70%) | 116 (71%) | 0.9 |
| Smoking: Current | 343 (90%) | 104 (88%) | 91 (92%) | 148 (91%) | 0.6 |
| **Medication** |  |  |  |  |  |
| Ace Inhibitor | 258 (68%) | 76 (65%) | 69 (69%) | 113 (69%) | 0.7 |
| Angiotensin II receptor blockers | 107 (28%) | 38 (32%) | 27 (27%) | 42 (26%) | 0.5 |
| Aldosteron antagonists | 293 (77%) | 90 (76%) | 73 (73%) | 130 (79%) | 0.5 |
| Diuretics other | 5 (1.3%) | 0 (0%) | 0 (0%) | 5 (3.0%) | **0.046** |
| Beta blockers | 350 (92%) | 102 (87%) | 92 (92%) | 156 (95%) | 0.056 |
| Aspirin | 77 (20%) | 21 (18%) | 19 (19%) | 37 (23%) | 0.6 |
| ^*^Median (IQR); n (%) | | | | | |
| ^†^Kruskal-Wallis rank sum test; Pearson's Chi-squared test; Fisher's exact test  BMI= Body mass index, PCI= Percutaneous coronary intervention, CABG= Coronary artery bypass surgery, ICD= implantable cardioverter-defibrillator, CRT= Cardiac resynchronisation therapy | | | | | |

### **Supplemental Table 12: Survival analysis using clusters based on the last measurements before PEP or censoring**

|  | Univariate | | Adjusted for Age, Sex and eGFR (CKD-EPI) | | | Adjusted for relevant Clinical Variables* | | | Adjusted for relevant Clinical Variables* and NT-proBNP | | |  |
| --- | --- | --- | --- | --- | --- | --- | --- | --- | --- | --- | --- | --- |
| **Characteristic** | **HR (95% CI)** | **p-value** | | **HR (95% CI)** | **p-value** | | **HR (95% CI)** | **p-value** | | **HR (95% CI)** | **p-value** | |
| subphenotype |  |  | |  |  | |  |  | |  |  | |
| 1 | — |  | | — |  | | — |  | | — |  | |
| 2 | 2.64 (1.61-4.32) | **<0.001** | | 2.24 (1.35-3.73) | **0.002** | | 1.85 (1.07-3.20) | **0.028** | | 1.36 (0.78-2.35) | 0.3 | |
| 3 | 1.46 (0.89-2.40) | 0.14 | | 1.70 (1.00-2.89) | 0.051 | | 1.72 (0.96-3.08) | 0.067 | | 2.16 (1.20-3.90) | **0.010** | |
| R² | 0.042 |  | | 0.060 |  | | 0.178 |  | | 0.314 |  | |
| AIC | 1,267 |  | | 1,120 |  | | 1,010 |  | | 955 |  | |
| BIC | 1,273 |  | | 1,133 |  | | 1,039 |  | | 986 |  | |
| c-index^†^ | 0.603 |  | | 0.629 |  | | 0.711 |  | | 0.795 |  | |
| HR = Hazard Ratio, CI=Confidence Interval, AIC= Akaike information criterion, BIC= Bayesian information criterion  * Age, Gender, eGFR (CKD-EPI), Systolic Blood Pressure, Duration of HF, NYHA class, history of atrial fibrillation, other arrhythmia and chronic renal failure  † Harrell’s bias correction | | | | | | | | | | | |  |

## **Supplemental references**

1. Gold L, Ayers D, Bertino J, Bock C, Bock A, Brody EN, et al. Aptamer-Based Multiplexed Proteomic Technology for Biomarker Discovery. PLoS One. 2010;5(12):e15004.

2. Kim CH, Tworoger SS, Stampfer MJ, Dillon ST, Gu X, Sawyer SJ, et al. Stability and reproducibility of proteomic profiles measured with an aptamer-based platform. Sci Rep. 2018;8(1):1-10.

3. Candia J, Cheung F, Kotliarov Y, Fantoni G, Sellers B, Griesman T, et al. Assessment of variability in the SOMAscan assay. Sci Rep. 2017;7(1):14248.

4. Williams SA, Kivimaki M, Langenberg C, Hingorani AD, Casas JP, Bouchard C, et al. Plasma protein patterns as comprehensive indicators of health. Nature medicine. 2019;25(12):1851-7.

5. Allaoui M, Kherfi ML, Cheriet A, editors. Considerably Improving Clustering Algorithms Using UMAP Dimensionality Reduction Technique: A Comparative Study. International Conference on Image and Signal Processing; 2020: Springer.

6. McInnes L, Healy J, Saul N, Großberger L. UMAP: Uniform Manifold Approximation and Projection. Journal of Open Source Software. 2018;3(29):861.
